# Cytosine base modifications regulate DNA duplex stability and metabolism

**DOI:** 10.1093/nar/gkab509

**Published:** 2021-06-16

**Authors:** Cathia Rausch, Peng Zhang, Corella S Casas-Delucchi, Julia L Daiß, Christoph Engel, Gideon Coster, Florian D Hastert, Patrick Weber, M Cristina Cardoso

**Affiliations:** Cell Biology and Epigenetics, Department of Biology, Technical University of Darmstadt, 64287 Darmstadt, Germany; Cell Biology and Epigenetics, Department of Biology, Technical University of Darmstadt, 64287 Darmstadt, Germany; Center for Tissue Engineering and Stem Cell Research, Guizhou Medical University, Guiyang, Guizhou 550004, China; Chester Beatty Laboratories, The Institute of Cancer Research, London SW3 6JB, UK; Regensburg Center for Biochemistry, University of Regensburg, 93053 Regensburg, Germany; Regensburg Center for Biochemistry, University of Regensburg, 93053 Regensburg, Germany; Chester Beatty Laboratories, The Institute of Cancer Research, London SW3 6JB, UK; Cell Biology and Epigenetics, Department of Biology, Technical University of Darmstadt, 64287 Darmstadt, Germany; Cell Biology and Epigenetics, Department of Biology, Technical University of Darmstadt, 64287 Darmstadt, Germany; Cell Biology and Epigenetics, Department of Biology, Technical University of Darmstadt, 64287 Darmstadt, Germany

## Abstract

DNA base modifications diversify the genome and are essential players in development. Yet, their influence on DNA physical properties and the ensuing effects on genome metabolism are poorly understood. Here, we focus on the interplay of cytosine modifications and DNA processes. We show by a combination of *in vitro* reactions with well-defined protein compositions and conditions, and *in vivo* experiments within the complex networks of the cell that cytosine methylation stabilizes the DNA helix, increasing its melting temperature and reducing DNA helicase and RNA/DNA polymerase speed. Oxidation of methylated cytosine, however, reverts the duplex stabilizing and genome metabolic effects to the level of unmodified cytosine. We detect this effect with DNA replication and transcription proteins originating from different species, ranging from prokaryotic and viral to the eukaryotic yeast and mammalian proteins. Accordingly, lack of cytosine methylation increases replication fork speed by enhancing DNA helicase unwinding speed in cells. We further validate that this cannot simply be explained by altered global DNA decondensation, changes in histone marks or chromatin structure and accessibility. We propose that the variegated deposition of cytosine modifications along the genome regulates DNA helix stability, thereby providing an elementary mechanism for local fine-tuning of DNA metabolism.

## INTRODUCTION

It has long been known that besides the four canonical DNA bases, adenine (A), thymine (T), cytosine (C) and guanine (G), chemically modified variants are found within the genome of almost all phyla (1). The predominant DNA modification within the mammalian genome is cytosine methylation, consisting of the addition of a methyl group to the C5 of the cytosine ring [5-methylcytosine (5mC) (2)], and catalyzed by DNA methyltransferases (Dnmts) (3,4). In mammals, cytosine methylation is involved in numerous cellular processes, such as X-chromosome inactivation and imprinting, silencing of gene expression, control of cell development and differentiation (5–7). The impact of DNA methylation on gene expression and ultimately cellular differentiation stresses the need for a tight regulation of d5mC levels. Oxidation of d5mC to 5-hydroxymethylcytosine (d5hmC) (8) and the identification of the responsible enzymes, the ten-eleven Translocation protein family (Tet1, Tet2 and Tet3) (9), established a long sought active DNA demethylation pathway dependent on thymine-DNA glycosylase (TDG) and base excision repair (BER) (10–12). Tet proteins catalyze the oxidation of 5mC to 5hmC in a 2-oxoglutarate-, iron(II)- and oxygen-dependent manner and were shown to further oxidize 5hmC to 5-formylcytosine (5fC) and 5-carboxylcytosine (5caC) (13) in DNA. Subsequent studies could identify factors that specifically recognize and bind modified cytosine bases (epigenetic readers) (14), thereby regulating chromatin structure, DNA accessibility and gene expression (15,16).

Generally, cytosine base modifications are thought to not influence the global canonical B-DNA structure, although local changes around the modification site can arise [reviewed in (17)]. Recent reports, however, show that chemically modified bases can directly affect primary DNA double helix structure and conformation *in vitro* (18), as well as flexibility (19) and thermostability (20). Yet, the influence of base modifications on DNA structure and stability is still highly debated (21,22). These chemical modifications of DNA bases do not alter the Watson–Crick base pairing per se; they, however, add a chemical functionality within the DNA double helix, changing the hydrophobicity of the major groove, introducing steric hindrance and changing base pair stacking, structure and mechanical properties of the DNA double helix (18,22–27). Interestingly, the size and especially the hydrophobicity of the modifications influence fluctuations and rotary motions within the base pairs and are essential determinants of the solvation state of the DNA stretch and consequently of its rigidity and stability (28).

Since an alteration in DNA stability and shape would be predicted to have major consequences for global genome processes, we investigated the effects of cytosine modifications on DNA double helix stability and DNA-related enzymatic processes *in vitro* and *in vivo*. We therefore analyzed, on the one hand, DNA melting temperature *in vitro* and, on the other hand, manipulated genomic DNA base modification levels *in vivo*. To this end, we altered expression levels of modification writer proteins (Dnmts and Tets) and transfected cells with modified nucleotides. Overall, methylated cytosines resulted in higher melting temperatures, slower helicase-mediated DNA unwinding and decreased rates of DNA replication and transcription. In contrast, its oxidation product d5hmC reverted this negative effect and led to lower melting temperatures, and overall higher DNA unwinding, and replication and transcription rates. We additionally show that the loss of DNA modifications in mouse embryonic stem cells altered S-phase progression, due to faster replication fork speed in the absence of d5mC. These results imply that, in addition to previously described effects of DNA modifications on chromatin structure, their effect on the stability of the DNA duplex per se creates an additional way by which DNA base modifications can influence genomic processes, thereby (locally) fine-tuning DNA accessibility and enzymatic activity.

## MATERIALS AND METHODS

### Plasmids

Mammalian expression constructs encoding a mcherry-tagged fusion of the catalytic domain of mouse Tet1 (Tet1CD) and the mcherry tag alone were previously described (15). GFP-tagged human RPA34 (29) and RFP-tagged human proliferating cell nuclear antigen (PCNA) (30) or miRFP670-tagged human PCNA were used to study replication protein A (RPA) accumulation and to identify S-phase stages, respectively. The expression vector containing a monomeric near-infrared (miRFP670) PCNA fusion was generated by amplifying miRFP670 from the pmiRFP670-N1 [Addgene plasmid # 79887 and (31)] vector with primers containing AgeI and BsrGI sites (Supplementary Table S1). The fragment was inserted into AgeI/BsrGI-digested mRFP-hPCNA (30). The resulting plasmid was verified by restriction enzyme and DNA sequencing analyses. The correct subcellular localization was validated by microscopical analysis and the expected protein was detected by western blotting analysis upon expression into mammalian cells (Supplementary Figure S1). Supplementary Figure S2 and Supplementary Table S2 summarize the characteristics of fusion proteins and expression constructs.

To generate the plasmid containing the yeast ARS306 origin of replication (pGC504), two oligonucleotides (GC497 and GC498, Supplementary Table S1) were annealed and ligated into pZN3 (32) digested with BamHI and PstI.

### Cells, culture, transfection, inhibitor treatment and nucleoside/nucleotide incorporation

All cells used were tested for mycoplasma and deemed free of contamination. C2C12 mouse myoblast cells (33), mouse embryonic fibroblasts (MEFs) W8 (34) and PM [p53^−/−^ and Dnmt1^−/−^, (35)] and HEK 293 human embryonic kidney (36) cells were grown in Dulbecco’s modified Eagle Medium (high glucose, DMEM, Sigma-Aldrich, St Louis, MO, USA) supplemented with 20% (C2C12), 15% (MEF PM) or 10% (MEF W8 and HEK 293) fetal bovine serum, 1× glutamine (cat# G7513, Sigma-Aldrich, St Louis, MO, USA) and 1 μM gentamicin (cat# G1397, Sigma-Aldrich, St Louis, MO, USA) in a humidified atmosphere with 5% CO_2_ at 37°C. J1 wt and TKO (Dnmt1^−/−^, Dnmt3a^−/−^ and Dnmt3b^−/−^) mouse embryonic stem cells were cultured under feeder-free conditions on gelatin-coated culture dishes (0.2% gelatin in ddH_2_O) in DMEM (high glucose) supplemented with 16% fetal bovine serum, 1× nonessential amino acids (cat# M7145, Sigma-Aldrich, St Louis, MO, USA), 1× penicillin/streptomycin (cat# P4333, Sigma-Aldrich, St Louis, MO, USA), 2 mM l-glutamine, 0.1 mM β-mercaptoethanol (cat# 4227, Carl Roth, Karlsruhe, Germany), 1000 U/ml LIF (cat# ESG1107, Merck, Kenilworth, NJ, USA), 1 μM PD0325901 and 3 μM CHIR99021 (Axon Medchem BV, Reston, VA, USA). Supplementary Table S3 summarizes the cell line characteristics.

Transfections of C2C12 and MEF mouse cells with expression plasmids were performed using AMAXA nucleofection (AMAXA Nucleofector II. Lonza, Basel, Switzerland) as described previously (37). Modified nucleotides (dCTP, d5mCTP and d5hmCTP) were added at a final concentration of 0.2 mM and Cy3-dUTP at a final concentration of 0.02 mM to the transfection mix (cells resuspended in 5 mM KCl, 15 mM MgCl_2_, 120 mM Na_2_HPO_4_/NaH_2_PO_4_, pH 7.2, and 50 mM mannitol). Cells were analyzed 24 h after transfection. Nucleotide characteristics are summarized in Supplementary Table S4.

Cells used for immunofluorescence were grown on glass coverslips. For time-lapse microscopy, cells were plated in μ-Slide eight-well slides (Ibidi GmbH, Planegg/Martinsried, Germany).

Trichostatin A (TSA, cat# T8552, Sigma-Aldrich, St Louis, MO, USA) treatment was performed with a final concentration of 20 nM for 72 h changing with fresh TSA containing medium every day, as described in (38).

EdU (5-ethynyl-2′-deoxyuridine) and EU (5-ethynyl-uridine) labeling and the subsequent detection of nascent DNA/RNA are described below.

### Growth curve analysis, doubling time, cell cycle distribution and progression

For growth curve analysis, 2 × 10^5^ J1 wt and TKO cells were seeded as technical quadruplicates at day 0, and cell numbers were counted with a Neubauer hemocytometer for four consecutive days. Population doubling times were derived with log2(*n*_*x*_/*n*_0_)/*t*[h] (*n*_x_: cell number at day *x*, n_0_: cell number at day 0, *t*: hours after seeding). To determine the percentage of cells in every cell cycle and S-phase substage, asynchronously growing J1 wt and TKO cell cultures were pulse labeled with 10 μM EdU for 12 min, fixed and EdU was detected as described below. Cells were manually grouped into the S-phase substages (stages I to Y, (39)), non S-phase or mitosis, and percentages were calculated. S-phase (substage) duration was derived by multiplying the doubling time with the percentage of cells in the respective phase. To compare the spatio-temporal progression through S-phase, J1 wt and TKO cells were pulse labeled for 12 min with 10 μM EdU, washed twice and chased for 2 h with 50 μM thymidine (cat# T-1895, Sigma-Aldrich, St Louis, MO, USA). Fixation, permeabilization and MeOH treatment were done as described for immunofluorescence. EdU was detected prior to PCNA detection with mouse anti-PCNA and donkey anti-mouse IgG Cy3. All antibody characteristics are summarized in Supplementary Table S5.

### Immunofluorescence

C2C12, MEF W8 and PM and J1 wt and TKO cells were fixed in 3.7% formaldehyde for 10 min and permeabilized for 20 min in 0.5% Triton X-100. For detection of 5mC, 5hmC, BrdU, H3 acetylation and histone modifications, cells were incubated for 5 min in ice-cold methanol. RNaseA treatment (10 μg/ml in 1× PBS [phosphate-buffered saline]) was performed for 30 min at 37°C and was followed by blocking in 0.2% fish skin gelatin (Sigma-Aldrich, St Louis, MO, USA) in 1× PBS. 5mC, 5hmC and BrdU were detected using mouse anti-5mC antibody, rabbit anti-5hmC antibody and rabbit anti-BrdU in conjunction with 25 U/ml DNaseI (Sigma-Aldrich, St Louis, MO, USA) for 60 min at 37°C in digestion/staining buffer (2% BSA, 30 mM Tris/HCl pH 8, 0.33 mM MgCl_2_, 0.5 mM β-mercaptoethanol). The following primary anti-histone antibodies were diluted in 1% BSA/PBS (bovine serum albumin in 1× phosphate-buffered saline) and incubated for 1 h at room temperature: rabbit anti-H3 acetyl, mouse anti-H3K9ac, rabbit anti-H3K18ac, rabbit anti-H3K56ac, rabbit anti-H4K8ac, rabbit anti-H4K16ac, rabbit anti-H3K9m3 and rabbit anti-H4K20m3. To stop DNaseI digestion, cells were washed with PBS containing 1 mM EDTA and 0.02% Tween-20. Following incubation with the secondary antibodies goat anti-rabbit IgG Chromeo 488, donkey anti-rabbit IgG Cy3, donkey anti-rabbit IgG Cy5, goat anti-mouse IgG Chromeo 488 or donkey anti-mouse IgG Cy3 for 60 min at room temperature, cells were washed with PBS containing 0.02% Tween-20. DNA was counterstained with 4,6-diamidino-2-phenylindole (DAPI, 1 μg/ml, cat# D9542, Sigma-Aldrich, St Louis, MO, USA) for 10 min, and cells were mounted in Mowiol 4–88 (cat# 81381, Sigma-Aldrich, St Louis, MO, USA) with 2.5% DABCO antifade (1,4-diazabicyclo[2.2.2]octan, cat# D27802, Sigma-Aldrich, St Louis, MO, USA). All antibody characteristics are summarized in Supplementary Table S5.

### Fluorescence *in situ* hybridization (FISH)

For the detection of major satellite DNA repeats, probes were amplified and labeled from mouse myoblast C2C12 genomic DNA by PCR (primer sequences in Supplementary Table S1) using self-made biotin dUTPs and Taq polymerase as described in (40). Probes were ethanol precipitated in the presence of 0.13 M sodium acetate and 50 μg/ml fish sperm DNA, resuspended in hybridization solution (70% formamide, 2× SSC [saline sodium citrate] and 10% dextran sulfate, pH 7), denatured at 80°C for 5 min and immediately put on ice. Cells were fixed for 10 min in 3.7% formaldehyde in 1× PBS, washed and subsequently permeabilized two times with 0.5% Triton X-100 for 15 min, equilibrated for 3 min in 2× SSC followed by 30 min in 50% formamide/2× SSC. After combining probes and cells in a metal chamber, denaturation was performed at the indicated temperatures with continuous temperature monitoring for 5 min in a water bath followed by overnight annealing at 37°C. Post-hybridization washing was performed with 2× SSC; blocking and antibody dilution were done with 1% BSA/4x SSC. To detect mcherry positive cells after FISH denaturation, rat anti-RFP antibody and donkey anti-rat IgG Cy3 (Supplementary Table S5) were used. Biotin was detected with Alexa488 conjugated streptavidin (1:800, cat# S11223, Invitrogen, PA49RF Paisley, UK) for 1 h at room temperature. DNA was counterstained and cells were mounted as described for immunofluorescence.

### EdU/EU labeling and visualization of nascent DNA/RNA

To analyze DNA replication, cells were labeled with 10 μM EdU (Supplementary Table S4) for 10, 20 or 30 min. To analyze DNA transcription, cells were labeled with 1 mM EU (Supplementary Table S4) for 25, 45, 60 or 120 min. Cells were fixed 10 min in 3.7% formaldehyde. EdU and EU were detected using the Click-IT assay (Thermo Fisher Scientific, Waltham, MA, USA) with 6-FAM azide (1:1000) according to the manufacturer’s instructions. DNA was counterstained and cells were mounted as described for immunofluorescence.

### DNA preparation

All oligonucleotide, primer, plasmid and nucleotide characteristics are summarized in Supplementary Tables S1, S2 and S4, respectively. PCR products used in HRM, dot blot, DNA/RNA polymerization and exonuclease assays were purified by gel electrophoresis followed by silica column purification using the QIAquick PCR Purification Kit (Qiagen, Hilden, Germany). Template purification for soluble *in vitro* replication assays with yeast proteins was performed using phenol/chloroform (described below).

Genomic DNA (gDNA) for FISH probe generation was prepared as previously described (40).

#### DNA oligonucleotide annealing

DNA oligonucleotides (20-mer) for high-resolution melting temperature (HRM) analysis were purchased from IBA Lifescience (Goettingen, Germany) and IDT (Integrated DNA Technologies, Skokie, Il, USA). Oligonucleotide sequences are summarized in Supplementary Table S1. To prepare double-stranded DNA oligonucleotides, upper and lower oligos were mixed at equal molar amounts in NEBuffer 2 (50 mM NaCl, 10 mM Tris/HCl, 10 mM MgCl_2_, 1 mM dithiothreitol (DTT); NEB, Ipswich, MA, USA), denatured at 95°C for 2 min and annealed by slowly cooling down to room temperature.

#### PCR fragments for HRM, DNA polymerization and exonuclease assays

PCR fragments for HRM analysis were amplified using Q5 polymerase (cat# M0491, NEB, Ipswich, MA, USA) and dCTP, d5mCTP, d5hmCTP, d5fCTP or d5caCTP from a plasmid containing the MINX sequence ((41), Supplementary Table S2) with MINX specific primers or from human HEK gDNA with LINE1 promoter primers. Cycles were set to 98°C for 2 min, 40× (98°C for 15 s, 62.5°C for 30 s, 72°C for 40 s), 72°C for 2 min. To amplify d5fC- and d5caC-containing products, the following cycle conditions were used: 98°C for 1 min, 45× (98°C for 10 s, 65.2°C for 30 s, 72°C for 40 s), 72°C for 2 min.

PCR fragments for DNA polymerization and exonuclease assays were amplified as described above and using dCTP, d5mCTP or d5hmCTP and Q5 polymerase.

#### DNA templates for *in vitro* DNA replication assays

DNA templates (3 kb) for soluble DNA replication assays with eukaryotic replisome components were generated using Q5 polymerase and dCTP, d5mCTP or d5hmCTP from a plasmid containing the yeast ARS306 origin (pGC504 ARS306) with specific pGC504 primers. Primers were designed in a way to generate amplicons with centrally arranged ARS306. Additionally, to generate amplicons with different levels of cytosine modifications, reactions containing 100% (100% C_modif_ and 0% C), 50% (50% C_modif_ and 50% C) or 12.5% (12.5% C_modif_ and 87.5% C) d5mCTP or d5hmCTP were set up. Cycles were set to 98°C for 2 min, 40× (98°C for 20 s, 68.4°C for 30 s, 72°C for 100 s), 72°C for 10 min. All reactions were checked on agarose gels (0.3 μg/ml ethidium bromide), DNA was extracted from the reactions using phenol/chloroform/isoamyl alcohol (cat# 3215, Carl Roth, Karlsruhe, Germany and cat# A1935, AppliChem, Darmstadt, Germany) and ethanol precipitated in the presence of 0.3 M sodium acetate.

#### DNA templates for *in vitro* DNA transcription assays

PCR fragments for T7 RNA polymerization reactions were amplified using dCTP, d5mCTP or d5hmCTP, MINX plasmid as template and Q5 polymerase. For the generation of the T7 transcription assays, the forward primer binds the T7 promoter region, therefore, the resulting T7 promoter region amplicon contained unmodified cytosines on the forward strand, allowing T7 RNA polymerase-mediated transcription.

PCR fragments for tailed template elongation with eukaryotic RNA polymerase were amplified using dCTP or 100% d5mCTP or d5hmCTP, MINX plasmid as template and Q5 polymerase. Nb.BsmI nicking site containing forward primer and the corresponding reverse primer are listed in Supplementary Table S1.

### PCR efficiency and yield assays

PCR efficiency and yield assays contained MINX or LINE1 primers and MINX plasmid or HEK gDNA as template, respectively, dATP, dTTP, dGTP and dCTP, d5mCTP or d5hmCTP and the indicated polymerases. DNA denaturing was performed at the indicated temperatures. Agarose gels were stained with 0.3 μg/ml ethidium bromide (EtBr, cat# 2218, Carl Roth, Karlsruhe, Germany) in 1× TAE (tris acetic acid buffer, 40 mM Tris, 1 mM EDTA, 20 mM glacial acetic acid) for 10 min, destained for 5 min in ddH_2_O and imaged using a AI600 imager (Supplementary Table S6).

### Dot and western blot analysis

Dot blot analysis of modified PCR fragments was performed as described (15). For PCR fragment analysis, 20, 50, 75 or 150 ng of DNA was spotted on a nitrocellulose membrane. To confirm (equal) DNA loading, the membrane was stained for 2 min in 0.2% methylene blue (cat# M9140, Sigma-Aldrich, St Louis, MO, USA) in 0.3 M sodium acetate, pH 5.2, followed by destaining in ddH_2_O. On a separate membrane, the modifications were detected with primary mouse anti-5mC, rabbit anti-5hmC, rabbit anti-5fC and rabbit anti-5caC antibodies, respectively, and secondary sheep anti-mouse IgG HRP (horseradish peroxidase) conjugated or goat anti-rabbit IgG HRP conjugated antibodies.

To analyze the miRFP-hPCNA (pc3385) fusion protein, (transfected) HEK293 cells were lysed in 4× SDS loading buffer (200 mM Tris/HCl pH 6.8, 400 mM DTT, 8% SDS, 0.4% bromophenol blue and 40% glycerol), and whole protein lysates were analyzed by SDS-PAGE and blotted on a nitrocellulose membrane (GE Healthcare, Chicago, Il, USA). PCNA was detected using a mouse anti-PCNA primary antibody and a sheep anti-mouse IgG HRP conjugated secondary antibody. Visualization of immunoreactive bands was achieved by ECL plus Western Blot detection reagent (GE Healthcare, Munich, Germany).

Membranes were imaged with the CCD camera of an AI600 imager (Supplementary Table S6) and antibody characteristics are summarized in Supplementary Table S5.

### *In vitro* DNA and RNA polymerization and DNA exonuclease assay with prokaryotic and phage enzymes

#### DNA polymerization assays using prokaryotic enzymes

DNA polymerization reactions contained LINE1 PCR amplicons containing dC, d5mC or d5hmC, respectively, as DNA template, LINE1 forward and reverse primers (Supplementary Table S1) and dATP, dTTP, dGTP and dCTP (Supplementary Table S4), respectively, and were incubated for 3 min at 99°C to denature the dsDNA template. Primer annealing was done for 1 min at 85°C. Reactions were cooled down to 37°C, and 0 U, 0.0016 U, 0.008 U, 0.04 U, 0.2 U or 1 U of Klenow polymerase (cat# M0210, NEB, Ipswich, MA, USA) was added and incubated for 20 min to allow DNA polymerization. Products were analyzed on a 2% agarose gel; band intensities were measured (readout for DNA polymerization rate) and plotted.

#### DNA exonuclease assays

DNA exonuclease reactions contained MINX PCR amplicons with dC, d5mC or d5hmC, respectively, as DNA template and 0 U, 0.03125 U, 0.0625 U, 0.125 U, 0.25 U or 0.5 U of T4 DNA polymerase (cat# M0203, NEB, Ipswich, MA, USA) was added and incubated for 7 min at room temperature. Exonuclease activity was analyzed on a 2% agarose gel, and band intensities were measured (readout for DNA exonuclease rate) and plotted.

All agarose gels were imaged using an AI600 imager (Supplementary Table S6), integrated band intensities were measured using ImageJ (https://imagej.nih.gov/ij/), and background was subtracted and plotted as a ratio to the control (DNA without enzyme).

#### RNA polymerization assays using phage enzymes

To generate single-stranded DNA (ssDNA) templates for *in vitro* transcription reactions (IVT), 5′ phosphorylated (forward strand, Supplementary Table S1) PCR amplicons were digested with λ exonuclease (cat# EN0561, Thermo Fisher, Waltham, MA, USA). The ssDNA generated was purified by ethanol precipitation as described in (40) and in the presence of 80 μg glycogen (cat# 10901393001, Sigma Aldrich, St Louis, MO, USA). To obtain a double-stranded (dsDNA) T7 promoter region, a forward oligonucleotide containing the T7 promoter (Supplementary Table S1) was annealed to the ssDNA fragments. Therefore, equimolar amounts of ssDNA and oligonucleotide were mixed, heated for 3 min at 95°C and slowly cooled down to room temperature to allow annealing.

For RNA polymerization assays via T7 run-off IVT, PCR amplicons containing dC, d5mC or d5hmC respectively (dsDNA or ssDNA) DNA template, ATP, UTP, GTP and CTP (Supplementary Table S4), T7 RNA polymerase (cat# M0251, NEB, Ipswich, MA, USA) and 0.5× SYBR Green II (cat# S-7564, Thermo Fisher, Waltham, MA, USA) were used. Cycling reactions were performed in a StepOnePlus^TM^ Real-Time PCR machine (Applied Biosystems), SYBR Green II intensities were measured every 30 s for 450 min and plotted using RStudio (version 1.1.447).

### Soluble *in vitro* DNA replication assays with purified eukaryotic replisome components

DNA replication assays with yeast replication proteins were performed at 30°C and as described in (42) with the following changes: MCM loading and phosphorylation were performed (5 μl per lane) in a buffer containing 25 mM HEPES-KOH (pH 7.6), 100 mM potassium glutamate, 10 mM magnesium acetate, 100 μg/ml BSA, 2 mM DTT, 0.01% NP-40-S, 5 mM ATP, 45 nM Cdc6, 20 nM origin recognition complex (ORC), 75 nM Cdt1-Mcm2-7, 50 nM DBF4 dependent kinase (DDK) and 5 nM linear DNA template (described above). After 20 min incubation, the reaction volume was increased 2-fold by the addition of pre-equilibrated buffer to give a final replication reaction buffer of 25 mM HEPES-KOH (pH 7.6), 100 mM potassium glutamate, 10 mM magnesium acetate, 100 μg/ml BSA, 1 mM DTT, 0.01% NP40-S, 3 mM ATP, 22.5 nM Cdc6, 10 nM ORC, 37.5 nM Cdt1-Mcm2-7, 25 nM DDK, 2.5 nM linear DNA template, 200 μM CTP, GTP and UTP, 30 μM dCTP, dGTP, dATP and dTTP and 33 nM α^33^P-dATP. Replication was initiated by adding a master mix of proteins to achieve final concentrations of 20 nM Sld3/7, 20 nM Sld2, 30 nM Dpb11, 100 nM GINS, 40 nM Cdc45, 20 nM Pol ϵ, 10 nM Mcm10, 40 nM RPA, 10 nM S-CDK, 40 nM Pol α, 25 nM Csm3/Tof1, 25 nM Mrc1, 30 nM RFC, 40 nM PCNA and 5 nM Pol δ. Nucleotide characteristics are summarized in Supplementary Table S4. The replication reaction was stopped after 15 min by the addition of 100 mM EDTA, separated on 0.8% alkaline agarose gels as described in (43) and signals detected using a Typhoon FLA-9500 (Supplementary Table S6).

### *In vitro* tailed template transcription (RNA polymerization) assays with purified eukaryotic DNA-dependent RNA polymerase I

Tailed template PCR products (described above) were purified using the QIAquick PCR purification kit (cat# 28104, Qiagen, Hilden, Germany). Purified templates were cut with the nicking endonuclease Nb.BsmI (cat# R0706S, NEB, Ipswich, MA, USA) at 65°C for 2 h. The reaction was inactivated at 80°C for 20 min. After 10 min, a 10-fold molar excess of competitor oligo (Supplementary Table S1) was added and then cooled down slowly to anneal with the cleaved-off single-stranded fragment to obtain a 3′-overhang on the template. The DNA was ethanol-precipitated, dried and resuspended in water to reach a concentration of 125 nM.

*Saccharomyces cerevisiae* DNA-dependent RNA polymerase (Pol) I was purified as described (44,45). The polymerization assay is based on previously published protocols (46). Per transcription reaction 0.25 pmol Pol I, 0.125 pmol template (described above), 50 pmol GpC dinucleotide and HEPES (pH 7.8; end concentration 50 mM) were mixed. Five transcription reactions (final volume 25 μl) were treated as one and started by adding the equal amount of 2× transcription buffer (40 mM HEPES, pH 7.8, 10 mM MgCl_2_, 5 mM EGTA, 0.05 mM EDTA, 2.5 mM DTT, 0.2 mM ATP, 0.2 mM UTP, 0.2 mM GTP, 0.01 mM CTP supplemented with 5 μCi of α^32^P-CTP per transcription reaction (0.0025 mM)). After 30, 60, 90, 120 and 600 s, 10 μl were removed and the reaction was stopped by adding the equal amount of 2× RNA loading dye (8 M urea, 2× TBE, 0.03% bromophenol blue, 0.03% xylene cyanol, 10 mM EDTA, 10 mM EGTA). The samples were heated to 95°C for 5 min, briefly centrifuged and applied to urea-PAGE (6% polyacrylamide gel containing 7 M urea in 1× TBE). The gel was dried for 30 min at 80°C in a vacuum dryer. Radiolabeled transcripts were detected by phosphor imaging (Supplementary Table S6). Nucleotide characteristics are summarized in Supplementary Table S4.

### High-resolution melting analysis (HRM)

To determine the melting temperature of 20-mer oligonucleotide containing single cytosine modifications and fully modified (except primer sites) PCR fragments, Platinum® SYBR® Green qPCR SuperMix-UDG w/ROX (cat# 11744100, Thermo Fisher, Waltham, MA, USA; UDG: uracil DNA glycosylase, ROX: 6-carboxyl-X-rhodamine) was diluted 1:1 with ddH_2_O, UDG was activated at 50°C for 2 min and added to 5 pmol of DNA samples. HRM temperature analysis was performed using a StepOnePlus™ Real-Time PCR machine (Applied Biosystems). Cycling conditions were set to 90°C for 30 s, then temperature was decreased to 50°C with a decreasing rate of 2%, followed by a temperature increase to 90°C with 0.1°C steps. Data normalization and plotting was performed using RStudio (version 1.1.447) and as described previously (47).

### Micrococcal nuclease (MNase) chromatin accessibility analysis

A total of 1 × 10^7^ cells were harvested and cell pellets were resuspended in hypotonic lysis buffer (10 mM Tris, pH 7.5, 10 mM NaCl, 3 mM MgCl_2_ and 0.5% NP-40) for 5 min on ice. Cell nuclei were pelleted (5 min, 120 × *g*), washed once in digestion buffer (50 mM Tris pH 8, 5 mM CaCl_2_) and resuspended in digestion buffer. MNase (cat# EN0181, Thermo Fisher Scientific, Waltham, MA, USA) was diluted in MNase storage buffer (20 mM Tris pH 7.6, 50 mM NaCl, 50% glycerol), and 0 U, 0.1 U, 0.5 U, 0.75 U, 1 U and 3 U were added to the nuclei in a final volume of 100 μl. Reactions were incubated for 5 min at 37°C, stopped by the addition of stopping buffer (20 mM EDTA, 0.4% SDS, 0.5 mg/ml proteinase K) and incubated at 50°C overnight. DNA was recovered by adding 0.1 volumes of 3 M NaCl and 2 volumes of 100% ethanol and storage at –20° for 1 h. After centrifugation (15 min, 15 000 × *g*) and washing with 70% ethanol, the DNA pellet was air-dried and resuspended in 50 μl ddH_2_O. Equal amounts of DNA were loaded on a 1.5% agarose gel and run for 45 min at 100 V. Gels were stained with EtBr in 1× TAE as described above and imaged using a AI600 imager (Supplementary Table S6). Integrated intensities of monomeric DNA and total DNA in each lane were measured with ImageJ and plotted as a DNA_mononmer_/DNA_total_ ratio.

### Replication labeling and molecular combing

Molecular combing experiments were performed using the FiberPrep®-DNA Extraction Kit (cat# EXTR-001, Genomic Vision, Bagneux, France) and as described by the manufacturer. In brief, wild-type J1 and knockout TKO mouse embryonic stem cells cells were first labeled for 15 min with 10 μM 5-iodo-2′-deoxyuridine (IdU, Supplementary Table S4), washed twice with pre-warmed PBS, labeled for 15 min with 100 μM 5-chloro-2′-deoxyuridine (CldU, Supplementary Table S4) and chased for 1 h with 50 μM thymidine (Supplementary Table S4). Cells were harvested by trypsinization and embedded in low melting point agarose (NuSieveGTG agarose, Biozym Scientific GmbH, Hessisch Oldendorf, Germany), genomic DNA was isolated by proteinase K digestion at a final concentration of 0.2 mg/ml, and single DNA molecules were stretched on vinylsilane-coated glass coverslips (cat# COV-002-RUO, Genomic Vision, Bagneux, France), using the FiberComb®-Molecular Combing System (cat# MCS-001, Genomic Vision, Bagneux, France) as described by the manufacturer.

Incorporated IdU and CldU nucleotides were immunofluorescently detected using mouse anti-BrdU/IdU and goat anti-mouse IgG Chromeo 546 before detecting CldU with rat anti-BrdU and donkey anti-rat IgG AlexaFluor 488. ssDNA was detected using mouse anti-ssDNA and goat anti-mouse IgG2a AlexaFluor 647. Samples were mounted with Vectashield antifade mounting medium (Vectorlabs, Burlingame, CA, USA). Antibody characteristics are summarized in Supplementary Table S5.

### Time-lapse microscopy

All characteristics of the microscopy systems, including lasers, filters and objectives used, are summarized in Supplementary Table S6.

To analyze RPA accumulation as a proxy for DNA helicase activity in cells, confocal z-stacks of live cells were acquired using the Ultra-View VoX spinning disk microscopy system. Time-lapse microscopy was carried out in a closed live-cell microscopy chamber at 37°C with 5% CO_2_ and 60% humidity. Somatic cells were grouped into early, mid and late S-phase cells according to their RPA and PCNA replication pattern (Supplementary Figure S1 and (48)). Somatic early S-phase cells are characterized by a homogeneous distribution of replication foci throughout the nucleus while in mid S-phase cells, replication signals are predominantly found around the nuclear and nucleolar border. In late S-phase cells, replication signals decrease in number while increasing in size and are co-localizing with highly condensed constitutive heterochromatin clusters (chromocenters). To analyze RPA accumulation in embryonic stem cells, only S-phase cells replicating chromocenters (stage II (39), Supplementary Figure S3) were taken into consideration. Cells were imaged once prior to adding aphidicolin (cat# A0781, Sigma Aldrich, St Louis, MO, USA) to a final concentration of 50 μg/ml (150 μM) or DMSO (cat# 41639, Sigma Aldrich, St Louis, MO, USA) as a control and subsequently imaged every 5 min for 30 min (long intervals) or every 10 s for 12 min (short intervals). To ensure the complete inhibition of the DNA polymerase by the aphidicolin, cells were incubated with 10 μM BrdU (Supplementary Table S4) for 10 min directly after imaging and fixed in 3.7% formaldehyde, and BrdU was detected as described above.

### High content screening, wide-field and confocal microscopy

All characteristics of the microscopy systems, including lasers, filters and objectives used, are summarized in Supplementary Table S6.

DAPI, 6-FAM, Alexa Fluor 488, mcherry and Cy3 signals were imaged using high content screening microscopy with 20× or 40× long working distance objectives.

Confocal z-stacks of fixed cells were acquired using the Ultra-View VoX spinning disk microscopy system.

Molecular combing samples were imaged using a Zeiss Axiovert 200 widefield microscope.

### Image analysis, statistical analysis and graphical representation

Z-stacks and montages of confocal microscopy images were created using ImageJ (https://imagej.nih.gov/ij/).

Full gel and blot images are depicted in Supplementary Figures S6 and S7.

#### High content screening microscopy analysis

For high content screening microscopy, image analysis was performed with the Harmony Software (PerkinElmer Life Sciences). Briefly, cell nuclei were segmented in the DAPI channel, creating DAPI masks that were used to segment the GFP and mcherry channels. For chromocenter segmentation, DAPI intense pericentric heterochromatin spots were detected (spot intensity > threshold) in the DAPI channel and the resulting mask was used to segment the GFP and mcherry channels. Cells touching the image border were excluded from the analysis. The analysis was further restricted to cells with a roundness coefficient > 0.8 and a nuclear area between 60 and 300 μm^2^. Lastly, intensity values for all three channels in the whole nucleus and in the chromocenters (Supplementary Figure S4) were measured and plotted with RStudio (version 1.1.447, https://rstudio.com/). Based on previous findings showing that the Tet1 levels of low Tet1CD-expressing cells and mouse embryonic stem cells (mESC) are equivalent (49), the group with low Tet1-expressing cells (fluorescence intensity 0–1000, group 1) was divided in three subgroups reflecting endogenous and physiological Tet1 expression levels (fluorescence intensity, group a: 0–100, group b: 100–500, group c: 500–1000), and the average intensity of single cells was plotted (Figure 2A). High Tet1CD-expressing cells were also divided in three groups (2: 1000–2000, 3: 2000–3000 and 4: >3000).

#### RPA accumulation analysis

To analyze the focal RPA accumulation upon aphidicolin/DMSO treatment, cell nuclei were segmented using the Volocity software (Version 6.3, Perkin Elmer), GFP-RPA intensities were measured and the coefficient of variation (*c*_V_ = σ/μ, with σ = standard deviation and μ = mean), as a proxy for helicase speed/activity was calculated for all time-points (Supplementary Figure S5). All values were normalized to the pretreatment *c*_V_ = *c*_V_(*tp*_x_)/*c*_V_(*tp*_0_) with *tp*_x_: any given time point imaged, *tp*_0_: pretreatment time point)*_._*and plotted using RStudio (version 1.1.447).

Slopes of the RPA accumulation curves were calculated as a ratio of the rise (difference of the *y*-coordinates) over the run (difference of the *x*-coordinates) of the respective linear regression line of the average RPA accumulation curves. To avoid taking plateau phases of RPA accumulation into consideration, slope calculations were restricted to the first 20 min of drug treatment (0–20 min).

#### DNA fiber analysis

Replication signals were measured using ImageJ, and replication fork speed (RFS) was calculated as a ratio of the track length (1 μm corresponds to 2 kb) and the time of nucleoside application. Fluorescent DNA fiber tracks were selected according to their pattern and only lengths of the second pulse (CldU) of progressing forks (CldU track preceded by a clear IdU signal) were considered.

#### DNA and RNA polymerization quantification

To quantify DNA polymerization in radioactive yeast replication reactions, the complete signal below the 3 kb end-labeling band was measured, background was corrected and for each template the respective –DDK signal was subtracted from the +DDK reaction. All values were normalized to the dC control template.

To quantify RNA polymerization in radioactive yeast transcription reactions, the signal intensity of the full-length transcript was determined for each reaction, using QuantityOne (4.6.6 Basic, Bio-Rad, Hercules, CA, USA). The background intensity was subtracted from each signal and then divided by the signal intensity of the full-length transcript generated from the unmodified dC template after 10 min (normalized signal intensity). The normalized signal intensities of 12 repeats performed with different Pol I purifications, different template preparations and different α^32^P-CTP batches were plotted using RStudio (version 1.1.447).

#### Statistical analysis

Independent two-group Student’s *t*-tests and Mann–Whitney–Wilcoxon tests were performed using RStudio (version 1.1.447). Statistical significance is indicated in the plots and detailed values for statistics [number of cells (*n*), median, mean, standard deviation (StDev), 95% confidence interval (95% CI) and *P*-values] are summarized in Supplementary Table S7 (main figures) and Supplementary Table S8 (supplementary figures).

#### Graphical representation

Boxplots and violin plots represent the median (center line) with the box depicting the 25–75 percentiles, and the dotted lines represent the upper and lower whiskers with 1.5 times the IQD (interquartile distance). Bar and line plots show normalized averaged values, and error bars show the respective standard deviation. HRM curves show the normalized average derivative of the technical replicates of one biological replicate over the temperature increase. Line intensity profile plots represent the fluorescence intensities along the distance of the selected arrow segment.

## RESULTS

### DNA base modifications affect DNA helix stability *in vitro*

**Figure 1.**
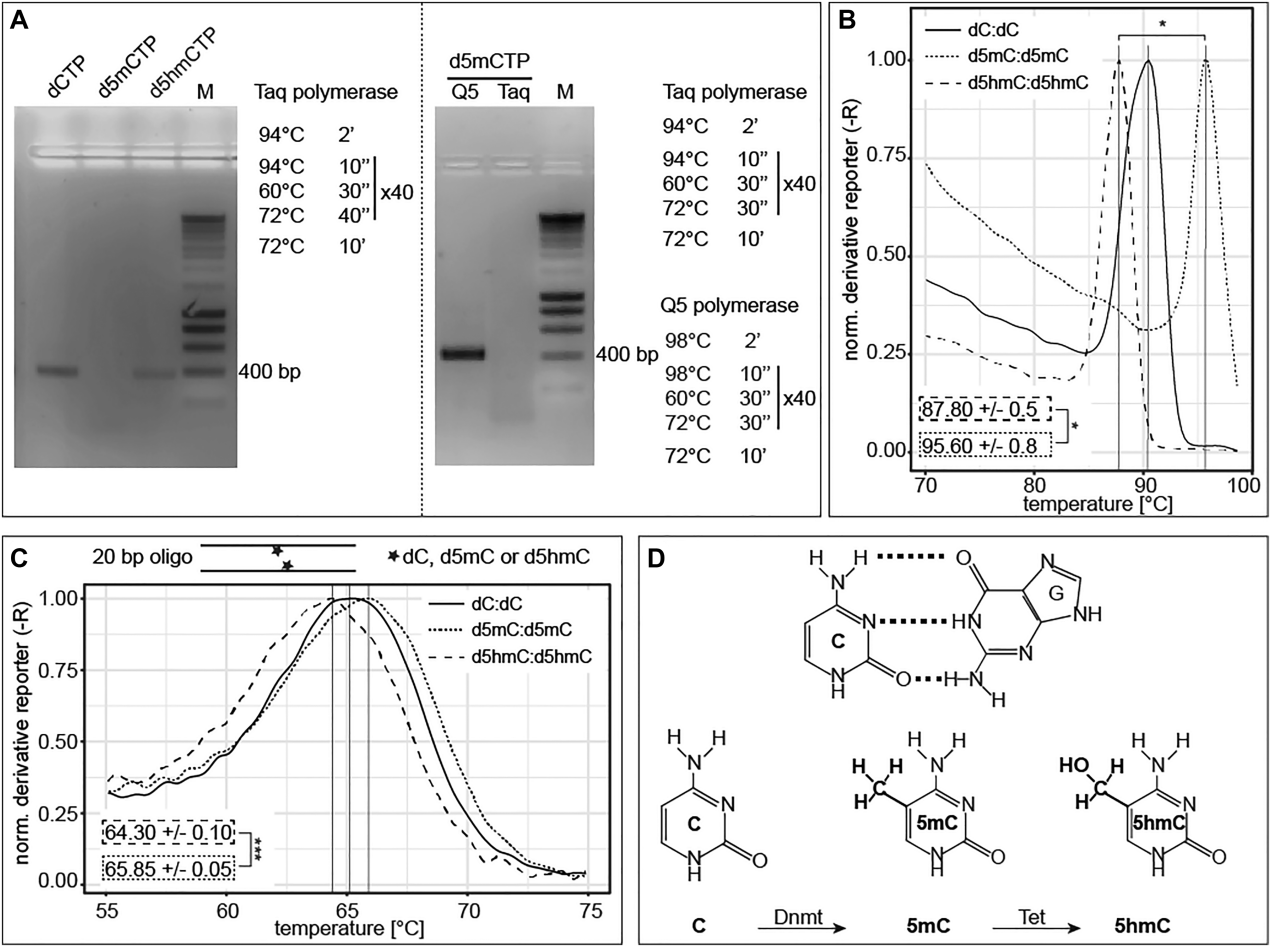
Effect of cytosine modifications on DNA double helix stability *in vitro*. (**A**) Agarose gel electrophoretic analysis of LINE1 PCR amplification products obtained with Taq polymerase, denaturing at 94°C and using dCTP, d5mCTP or d5hmCTP (left). Agarose gel electrophoretic analysis of LINE1 PCR amplification products obtained with Taq or Q5 polymerase, denaturing at 94 or 98°C and using d5mCTP (right). Complete PCR conditions are indicated. M stands for DNA size markers. (**B** and**C**) High-resolution melting temperature (HRM) analysis of LINE1 PCR fragments containing dC, d5mC or d5hmC, respectively, (B) and of 20 bp oligonucleotides containing one central (modified) CpG dinucleotide (CpG, 5mCpG or 5hmCpG) (C). Shown are the corresponding derivatives of the normalized mean SYBR green fluorescence intensity values of technical replicates over increasing temperature. Independent experiments were repeated in duplicates with three (B) or four (C) technical replicates, respectively, and one representative result is depicted. (**D**) Cytosine–guanine base pairings and (hydroxy)methylated cytosine bases with the respective writer enzymes are depicted. *P*-values were determined by Student’s *t-*test; *P < 0.05 and ***P < 0.001.

There is evidence that DNA base modifications by themselves might affect the DNA primary structure (18,24,50) and consequently may influence global genome processes, such as DNA replication, transcription and repair. In a first step, we investigated the stability of the double-stranded DNA helix *in vitro*. To that end, we performed polymerase chain reactions (PCR) using different DNA polymerases and modified base containing nucleotides. The replication of double-stranded DNA *in vitro* consists of three major steps: double-stranded DNA denaturation, primer annealing and primer elongation, where, in mammals, only the four canonical DNA bases are incorporated into newly synthetized DNA. All naturally occurring base modifications in mammalian DNA are generated post-replicatively by specific enzymes (3,4,9,13,51). To ensure that the DNA polymerase used in our *in vitro* experiments did not recognize incorporated modified nucleotides as mismatch and, consequently, removed the modified nucleotide via 3′ to 5′ exonuclease activity, we first analyzed the synthesis efficiency of the 3→5 exo^−^ Taq polymerase. Therefore, PCR reactions containing modified cytosine triphosphates were performed. All reactions contained deoxy-adenosine (dATP), deoxy-thymidine (dTTP) and deoxy-guanosine (dGTP) triphosphates and either deoxy-cytidine (dCTP), deoxy-5-methylcytidine (d5mCTP) or deoxy-5-hydroxymethylcytidine (d5hmCTP) triphosphates. Cycling conditions included denaturing steps at 94°C, as using higher denaturing temperatures decreased Taq polymerase activity/stability and consequently also PCR product yield (Supplementary Figure S8A). PCR reactions containing the standard dNTP mix (dATP, dTTP, dGTP and dCTP) resulted in one specific amplicon of 375 bp. Replacing dCTP with d5mCTP did not yield a PCR amplification product (Figure 1A, left), although Taq polymerase is able to incorporate d5mCTPs at higher denaturing temperatures (Supplementary Figure S8B). Several bacterial and archaeal polymerases have been shown to efficiently integrate d5mCTP (52), and family A polymerases like Taq and Klenow polymerase were shown to have similar incorporation rates for cytosines with small C5 substitutions and unmodified cytosine (53). These results demonstrate that the observed difference in PCR product amount between dCTP and d5mCTP containing reactions is not due to altered (modified) nucleotide affinity of the DNA polymerase (54). The use of d5hmCTP, Taq polymerase and denaturation at 94°C, however, resulted in a PCR product (Figure 1A, left). An amplification fragment was also obtained using d5mCTPs when the Taq polymerase (3′→5′ exo^−^) was replaced by Q5 polymerase (3′→5′ exo^+^), and the denaturing temperature in the cycling conditions was increased to 98°C (Figure 1A, right), which did not impair the activity of Q5 (Supplementary Figure S8A). Moreover, amplification using the Q5 polymerase at 98°C resulted in different yields of amplicon DNA depending on the cytosine modification (Supplementary Figure S8C and Supplementary Table S8). All modified nucleotides were also stably integrated in the PCR products as tested by slot blot analysis with antibodies specific to the respective base modifications (Supplementary Figure S8D). Altogether, these data suggest that cytosine modifications might themselves affect the denaturing temperature of the DNA. To directly test this, we measured the thermal double-stranded DNA stability using our established high-resolution melting (HRM) temperature assay (47). We first analyzed modified 375 bp long human LINE1 promoter PCR fragments (15) containing 222 cytosines (191 cytosines not included in the primer binding sites, i.e., modified) with 22 CpGs. We measured the highest melting temperatures for methylated PCR fragments followed by unmodified cytosine and d5hmC-containing amplicons (Figure 1B). d5caC-containing DNA fragments showed similar results to d5hmC, whereas d5fC showed the lowest melting temperatures (Supplementary Figure S8E and Supplementary Table S8). Similar results were obtained for HRM analysis of the 377 bp long MINX amplicon, containing 201 cytosines and 46 CpGs (188 cytosines and 42 CpGs not included in the primer regions) (Supplementary Figure S8F and Supplementary Table S8). Next, we analyzed the melting temperature of 20 bp DNA oligonucleotides containing a single central (modified) CpG (salt corrected melting temperature of 64.2°C). We also measured a significantly lower melting temperature for the 5hmCpG containing oligonucleotide in comparison to the 5mCpG containing one (Figure 1C). Furthermore, an even lower melting temperature for the 5fCpG oligonucleotide was observed (Supplementary Figure S8G), which is in accordance with our previous PCR amplicon HRM analysis. These results indicate that DNA modifications affect DNA double helix thermal stability *in vitro* in the way that methylated cytosines stabilize the DNA double helix, whereas d5hmC, d5caC and especially d5fC negatively influence DNA duplex stability. Our results (*T*_m_[d5hmC] < *T*_m_[dC] < *T*_m_[d5mC]) are in line with previous studies analyzing melting temperature of longer oligonucleotides with several modifications (20). However, 10mer duplexes showed lowest melting temperatures for unmodified DNA (55).

### Modified cytosine levels can be manipulated in cells

**Figure 2.**
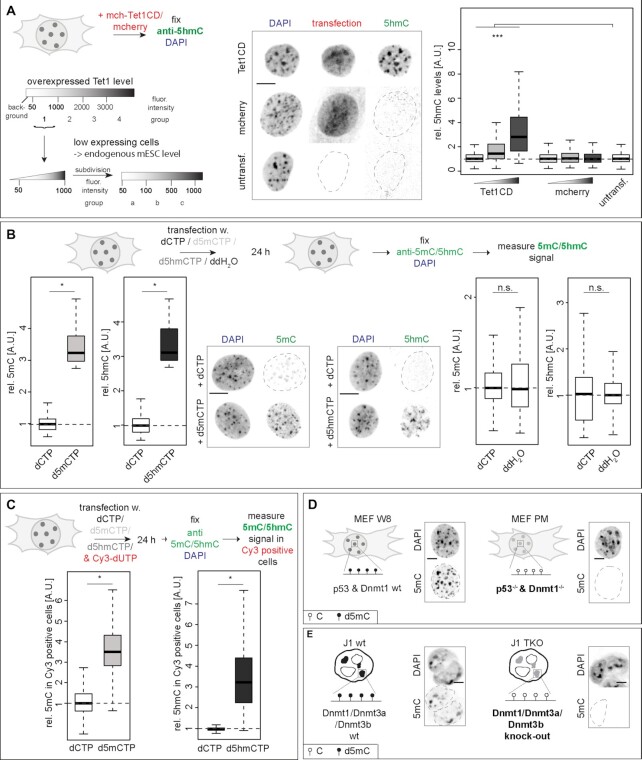
Manipulation of modified cytosine levels in cells. (**A**) Experimental setup to determine d5hmC levels in mch-Tet1CD overexpressing cells and representation of the cell grouping and binning approach applied. Only cells with Tet1 expression levels corresponding to group 1 (fluorescence intensity 50–1000, low Tet1 expressing cells) were analyzed and further subdivided in three subgroups (fluorescent intensities group a: 50–100, group b: 100–500 and group c: 500–1000) during plotting. Relative 5hmC sum fluorescence intensities in C2C12 cells, 24 h after transfection are shown. (**B** and**C**) Relative 5mC and 5hmC sum intensities within the whole (Cy3 positive) C2C12 nucleus 24 h after transfection with dCTP, d5mCTP, d5hmCTP or ddH_2_O (B) or co-transfection with Cy3-dUTPs (C) as indicated. (**D** and**E**) Schematic representation of the genotype and cytosine modification state in MEF W8 (wild-type) and MEF PM (*p53^−^^/^^−^* & *Dnmt1^−^^/^^−^*) cells (D) and in J1 mouse ES (wild-type) and J1 TKO (*Dnmt1^−^^/^^−^, Dnmt3a^−^^/^^−^* and *Dnmt3b^−^^/^^−^*) ES cells (E) and representative spinning disk confocal images of immunofluorescent detection of 5mC in wt and KO cells. Independent experiments were done in duplicates (B–E) or in triplicates (A) and *P* (determined by Student’s *t-*test) and *n*-values are summarized in Supplementary Table S7. All fluorescent signals are plotted as a ratio to the values of the respective control cells. All boxes and whiskers represent 25–75 percentiles and 1.5 times the IQD (interquartile distance), respectively, and the center line depicts the median. Dotted lines represent nuclear contours; A.U.: arbitrary units; scale bar = 5 μm; * *P* < 0.05, *** *P* < 0.001 and n.s.: non-significant.

In cells, levels of DNA base modifications can be manipulated by either changing the expression levels of the respective writer and/or modifier enzymes or introducing modified bases into the cell nucleus that will subsequently be incorporated into newly synthesized DNA. In mammalian cells, methylated cytosines are substrates of Tet dioxygenases ((9,13,51), Figure 1D and Supplementary Figure S8G). To increase d5hmC levels in DNA, mouse myoblast cells were transfected with the mcherry-tagged catalytic domain of Tet1 (Tet1CD), and 5hmC levels were quantified 24 h later using a 5hmC specific antibody. As shown before, further oxidized forms of d5mC have similarly helix destabilizing effects to d5hmC (Figure 1B and C, Supplementary Figures S8E–G and (20,25,26,55)) and, thus, would have similar effects on DNA strand separation. Previous studies showed that the Tet1 protein levels in low overexpressing cells are comparable to the endogenous Tet1 level in mES cells (49). We, therefore, restricted our analyses to these cells, as they represent a physiological relevant system. In these low Tet1CD-overexpressing cells, d5hmC levels significantly increased proportionally to Tet1CD protein levels, while the mcherry tag alone did not alter the d5hmC levels (Figure 2A and Supplementary Table S7). Importantly, contrary to Tet1 full-length (Tet1fl), Tet1CD lacks the large N-terminal regulatory domain, including the CXXC zinc finger. This allows Tet1CD binding not only to unmethylated but also to hypermethylated CGIs, thereby inducing significant global genomic d5mC oxidation. Additionally, Tet1CD catalytic activity is not biased to specific genomic regions and can act randomly throughout the methylated genome (56). Consequently, highly methylated pericentromeric heterochromatin (chromocenters) are targeted by Tet1CD and showed increased d5hmC levels in transfected cells (Figure 2A).

Since overexpression of Tet1CD leads to significantly increased levels of Tet1 protein in the cell nucleus and on chromatin, we selected a different additional approach to enhance the levels of modified cytosine bases in genomic DNA, relying on deoxynucleotide triphosphates (dNTPs) incorporation upon cell electroporation. Genomic d5mC and d5hmC levels significantly increased 24 h after cell transfection. Additionally, cell transfection with dCTP did not change d5mC or d5hmC levels. Co-transfection of dCTP, d5mCTP or d5hmCTP and Cy3-labeled dUTPs (Cy3-dUTP) resulted in d5mC and d5hmC increase in Cy3 positive cells (Figure 2B and C; Supplementary Table S7). The aforementioned approaches lead to an increase of the respective cytosine bases; hence, we also opted for loss of function experiments with concomitant lowering of the cytosine modification. Therefore, we chose *Dnmt1^−^^/^^−^* mouse embryonic fibroblasts (MEF PM) and Dnmt triple knockout (TKO) mouse embryonic stem (ES) cells that are depleted of all cytosine base modifications (Figure 2D and E and (35,57)).

In summary, with the above strategies we can efficiently up and down modulate cytosine modification levels in cells by interfering with levels of Tet1 and Dnmts (Supplementary Figure S9A and B) or by introducing modified cytosine bases into the cells (Supplementary Figure S9C).

### DNA base modifications affect double helix stability in cells

**Figure 3.**
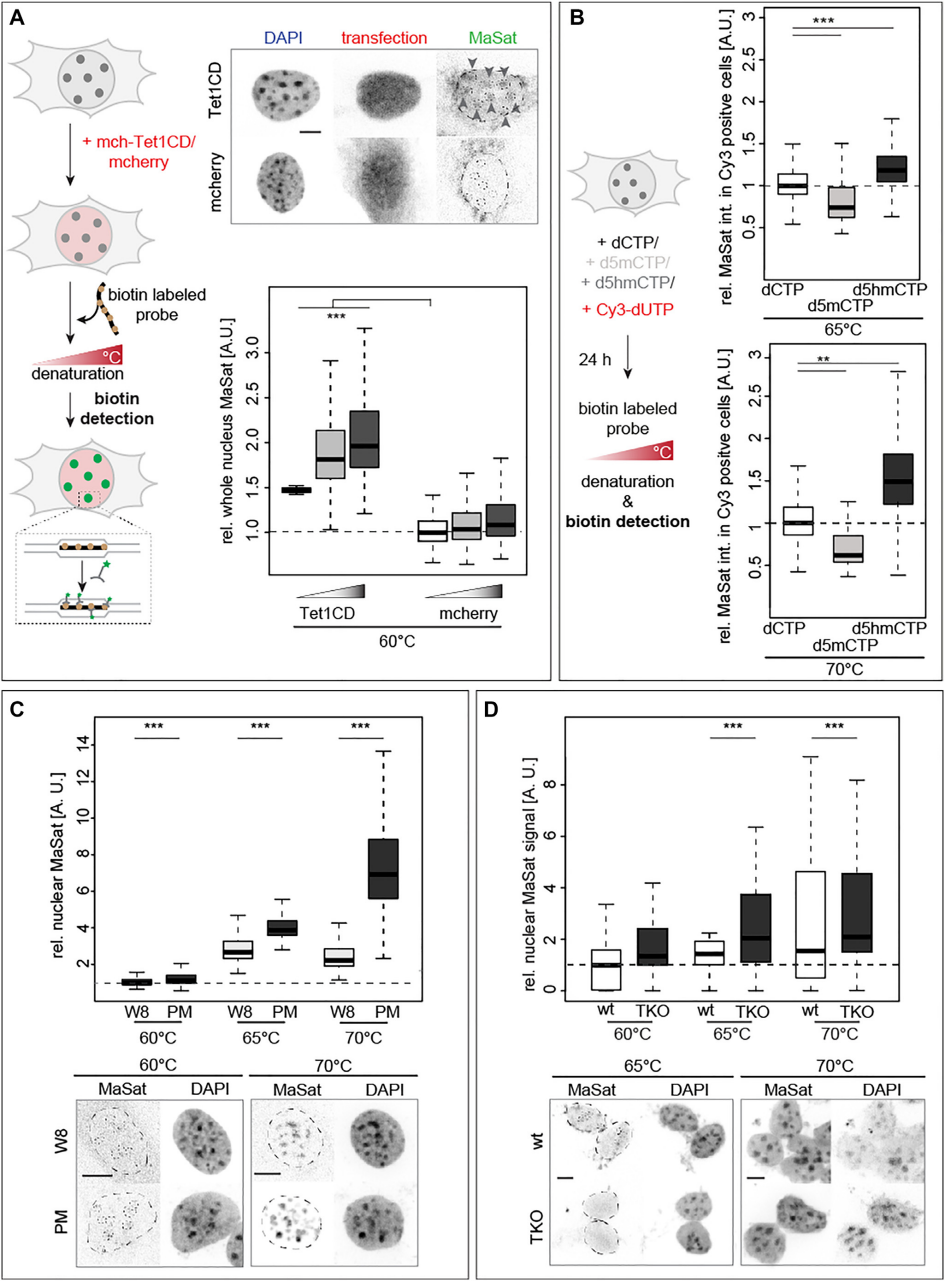
Effect of cytosine modifications on DNA double helix stability *in vivo*. (**A**) Major satellite (MaSat) DNA probes were used for fluorescence *in situ* hybridization (FISH) on transfected C2C12 mouse myoblast cells with genomic DNA denaturation performed at 60°C. Representative spinning disk confocal microscopy images with MaSat probe hybridization detection and DNA counterstaining are shown. Relative quantifications of the nuclear MaSat sum intensity are shown. (**B**) Relative major satellite DNA FISH sum intensities within the whole C2C12 nucleus of Cy3 positive cells at 65 or 70°C denaturation temperature 24 h after co-transfection with dCTP, d5mCTP, d5hmCTP and Cy3-dUTP as indicated. (**C** and**D**) Relative major satellite DNA FISH sum fluorescent intensities in MEF W8 and MEF PM nuclei (C) and in J1 wt and J1 TKO nuclei (D) at 60, 65 and 70°C denaturation; scale bars: 5 μm. Independent experiments were done twice (C and D) or in triplicates (A) and *P* (determined by Student’s *t-*test) and *n*-values (number of cells) are summarized in Supplementary Table S7. All boxes and whiskers are as in Figure 2. Dotted lines represent the nuclear or chromocenter contours. A.U.: arbitrary units; * *P* < 0.05, ** *P* < 0.005 and *** *P* < 0.001.

The next step was to analyze the DNA thermal stability differences in the above-mentioned cellular systems. We, therefore, made use of genomic DNA denaturation in cells via heat treatment followed by fluorescence*in situ* hybridization (FISH). We employed a DNA probe specific for pericentromeric heterochromatic major satellite DNA (MaSat, chromocenters), which contains high levels of d5mC (38,58). We first tested the temperature range in which *in situ* denaturation of the genomic DNA and, subsequently, annealing of the probe were possible. We, therefore, generated MaSat probes via PCR including biotin-conjugated dUTPs, combined them with fixed and permeabilized C2C12 mouse myoblast cells, denatured DNA at different temperatures (60, 70 and 80°C) and annealed the probe at 37°C. The highest MaSat signals were obtained at the optimal denaturing temperature of 80°C and MaSat signal decreased with decreasing denaturing temperatures (Supplementary Figure S10A and Supplementary Table S8). Nuclear and chromocentric MaSat FISH signal intensity increased significantly in Tet1CD but not in mcherry transfected cells when DNA was denatured at 60°C (Figure 3A, Supplementary Figure S10B and Supplementary Tables S7 and S8). Similar results were observed when DNA was denatured at 65 and 70°C (Supplementary Figure S10B and Supplementary Table S8).

To validate that our findings are not C2C12 mouse myoblast specific, we performed the FISH based assay also in Tet1CD transfected mouse embryonic fibroblast (MEF W8) cells, which yielded the same outcome of increased MaSat signal at low denaturation temperatures (65, 70 and 75°C) in Tet1CD transfected cells (Supplementary Figure S11A–C and Supplementary Table S8).

Previous studies found that cells enriched for d5hmC showed large and decondensed nuclei as well as decompacted heterochromatin (8,59,60). Since a more decondensed constitutive heterochromatin results in a more homogeneous DAPI staining throughout the nucleus and, in turn, to lower DAPI standard deviations, we measured the latter as a proxy for DNA decondensation. Indeed, increased levels of Tet1CD led to chromocenter decondensation (Supplementary Figure S10C and Supplementary Table S8). Therefore, we investigated whether the latter FISH results were due to Tet1CD induced chromatin decondensation facilitating DNA denaturation. Hence, we treated cells with trichostatin A (TSA), a potent histone deacetylase inhibitor. After 72 h of treatment, a significant increase in histone H3 acetylation and concomitantly chromatin decondensation was measured, which is in agreement with previous studies (38). d5hmC levels *per se* were not affected by TSA treatment and, importantly, TSA-treated cells did not show increased MaSat probe signal (Supplementary Figure S10D and Supplementary Table S8), indicating that chromatin decondensation does not facilitate thermal DNA denaturation.

To further rule out that we are measuring effects due to changes in overall chromatin structure, composition and accessibility as a consequence of Tet1CD overexpression, we performed the denaturation assays in cells transfected with nucleotides. In comparison to control-treated cells, increased d5mC levels led to significantly lower MaSat signals, whereas increased genomic d5hmC content increased the amount of probe annealing (Figure 3B and Supplementary Table S7).

Lastly, we performed the denaturation assays in the Dnmt loss of function systems (MEF PM and J1 TKO), where we observed a significant increase in MaSat probe signals (Figure 3C and D; Supplementary Table S7). This result is consistent with methylated DNA double helix being more stable than DNA containing unmodified cytosines.

Altogether these results indicate that modified cytosines influence DNA double helix stability, both negatively and positively. Methylated cytosines render the double helix more stable and, therefore, DNA denaturation needs more energy, i.e., higher temperatures, whereas the presence of d5hmC leads to an easier strand separation, due to a less stable DNA helix. Since global genome processes, such as DNA replication, transcription and repair, all require unwinding of the DNA double helix, alterations of DNA base-pairing stability could fundamentally influence these processes. We, therefore, aimed to analyze the impact of cytosine base modifications on RNA and DNA polymerization, as well as on DNA unwinding by helicase enzymes.

### DNA base modifications affect DNA transcription

**Figure 4.**
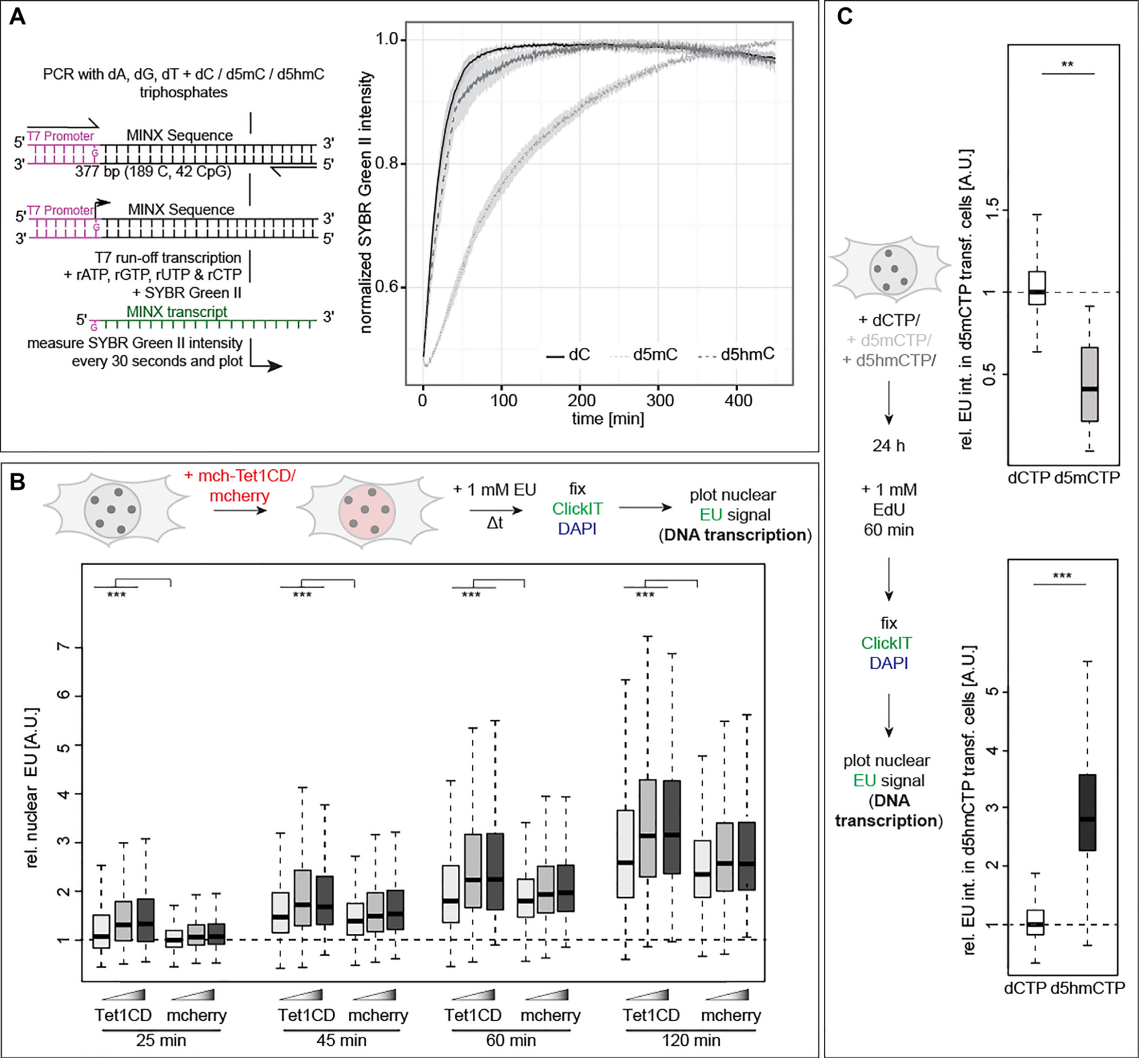
Effect of cytosine modifications on RNA polymerase activity. (**A**) Experimental setup for *in vitro* T7 run-off transcription. Template DNA contained 100% cytosine (dC), methylcytosine (d5mC) or hydroxymethylcytosine (d5hmC) and T7 promoter sequence was unmodified. Normalized mean SYBR green II intensities (± standard deviation), i.e., the amount of RNA transcripts, over time are shown. (**B**) Schematic representation of the experimental setup for *in vivo* ribonucleotide incorporation analysis. Cells were incubated with 1 mM EU 24 h after transfection with the constructs as indicated, EU was detected and the normalized nuclear EU sum intensities are plotted. (**C**) Schematic representation of the experimental setup for *in vivo* ribonucleotide incorporation analysis in dCTP, d5mCTP or d5hmCTP transfected cells. Cells were incubated with 1 mM EU 24 h after transfection with the nucleotides as indicated, EU was detected and the normalized nuclear EU sum intensities are plotted. Independent experiments were done twice (A and C) or in triplicates (B). All boxes and whiskers are as in Figure 2 and *P* and *n*-values are summarized in Supplementary Table S7; ** *P* < 0.005 and *** *P* < 0.001.

One of the major DNA metabolic processes within the cell nucleus is DNA transcription. As this ubiquitous process also depends on DNA double helix unwinding and the subsequent RNA polymerization by a DNA-dependent RNA polymerase, we addressed the effect of modified cytosines on RNA polymerase activity *in vitro*. We, therefore, performed an *in vitro* transcription assay using *in vitro* modified DNA templates containing an unmodified T7 promoter to avoid gene silencing in d5mC containing templates (61–65), followed by a MINX sequence and purified T7 RNA polymerase. The transcription activity of T7 RNA polymerase was detected in real-time with unmodified, methylated and hydroxy-methylated templates (Figure 4A). Importantly, high structural, functional and enzymatic similarities were reported among organisms for RNA polymerases I and II (66,67). We found that methylated templates resulted in significantly lower transcript amounts compared to unmodified or hydroxymethylated templates (Figure 4A). Since RNA polymerase elongation effects could be a concern in the *in vitro* transcription (IVT) assays, we performed similar experiments on (un)modified single-stranded (ssDNA) templates containing a double-stranded (dsDNA) T7 promoter region. Similarly to the dsDNA templates, we observed SYBR Green II fluorescence for the ssDNA templates, although the overall signal intensity, i.e. the overall amount of RNA transcripts, was lower than for dsDNA templates (Supplementary Figure S12A). Additionally, we measured no significant difference in template elongation between the unmodified and 5mdC- or 5hmdC-containing substrates. This is in agreement with previous studies revealing similar GTP incorporation rates opposite of dC, d5mC and d5hmC (68,69). We, therefore, conclude that the differences in transcription measured with the dsDNA template are due to changes in DNA strand separation, i.e. DNA melting, and not due to differences in elongation rates.

To further extend the *in vitro* transcription assays to eukaryotic enzymes, we analyzed *Saccharomyces cerevisiae* DNA-dependent RNA polymerase I (Pol I) transcription on (un)modified templates. The majority of the RNA produced in cells is produced by RNA polymerase I and, hence, this enzyme better represents the measurements *in vivo* (see below). To allow nonspecific RNA polymerase initiation, we generated templates containing a 3′-overhang (3′-tailed templates) based on the nicking enzyme Nb.BsmI (46). Slot blot analyses with antibodies specifically recognizing 5mC and 5hmC, respectively, confirmed the modification of the templates (Supplementary Figure S13A). *In vitro* transcription assays contained endogenous 14-subunit *S. cerevisiae* Pol I (44), and synthesized RNA was visualized and quantified by detecting the incorporated radiolabeled nucleotides, as a proxy for DNA transcription (Supplementary Figure S13A). We observed a clear increase of RNA products with increasing incubation time. The use of similar amounts of methylated templates, however, resulted in a clear trend indicating partial inhibition of transcription compared to unmodified templates. In contrast, d5hmC-containing DNA templates showed a reversion of transcription impairment and synthesized RNA amounts were similar to the dC containing control template (Supplementary Figure S13A). The fact that these effects can be detected even using budding yeast RNA polymerase, which in the natural environment of a yeast cell does not face modified DNA, denotes a high degree of evolutionary conservation of the enzymes involved in genome metabolism. Whereas the effect of mC-containing templates on Pol I transcription is subtle, a previous study showed that DNA methylation contributes to Pol I inhibition *in vivo* (70). Furthermore, Pol I transcription can overcome stronger opposing forces than Pol II *in vitro* (71), rationalizing the scale of the effects observed.

Next, we investigated the influence of cytosine base modifications on RNA polymerases and transcription rates *in vivo*. To this end, we incubated cells for various periods of time with EU (5-ethynyl-uridine), an uridine derivative that is incorporated into nascent RNA and can be detected via Click chemistry. The results showed a time dependent increase of EU signal (labeled RNA). Additionally, Tet1CD-transfected cells showed significantly higher EU levels than mcherry expressing cells, for all analyzed time points (Figure 4B and Supplementary Table S7). Since the nucleoli are subnuclear compartments where rDNA transcription by DNA dependent RNA polymerase I takes place, we additionally analyzed the EU levels in these specialized nuclear regions. Similar to whole nuclear EU results, we found elevated RNA levels in Tet1CD transfected cells (Supplementary Figure S12B and Supplementary Table S8). To relate these *in vivo* observations to the *in vitro* transcription assays, we also measured EU intensities in high Tet1CD expressing cells. Interestingly, and in accordance with the single enzyme reactions, we see similar levels of RNA in control and cells with high d5hmC levels (Supplementary Figure S13B).

We note, however, that the increased transcription in cells with high d5hmC levels may, in addition to the helix destabilizing effect, be due to the release of transcriptional inhibition by cytosine methylation and associated reader proteins (72). To bypass potential effects caused by ectopically overexpressing genes, we analyzed DNA transcription in nucleotide transfected cells. Nuclear EU levels decreased upon incorporation of d5mCTP into the genomic DNA, whereas the transcription rate increased upon elevating genomic d5hmC levels (Figure 4C and Supplementary Table S7). These results are in agreement with previous reports showing that DNA methylation inhibits transcription, whereas transcription is re-initiated upon oxidation of the d5mC. Moreover, this is also consistent with our findings that DNA metabolic processes are slowed upon DNA methylation due to helix stability increase whereas oxidation of d5mC to d5hmC results in a less stable DNA duplex facilitating DNA unwinding and subsequent DNA/RNA polymerization reactions.

Altogether these *in vitro* and *in vivo* data indicate that DNA transcription rates decrease in the presence of stabilizing d5mC with d5hmC reverting this inhibition and leading to increased synthesis of RNA.

### DNA base modifications affect DNA polymerase and exonuclease activities

**Figure 5.**
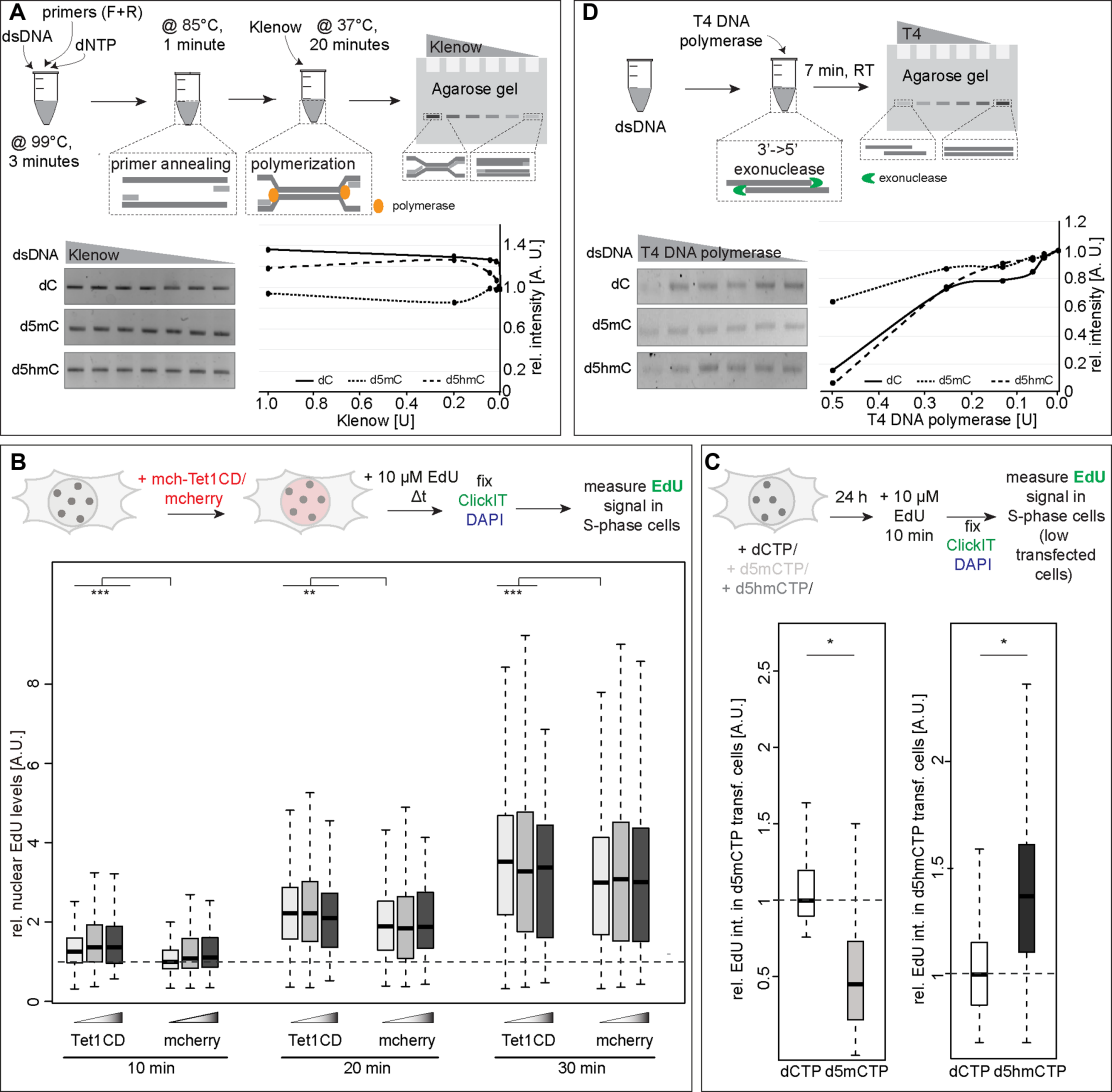
Effect of cytosine modifications on DNA polymerase activities. (**A** and**D**) Experimental setup for *in vitro* polymerization (A) and *in vitro* exonuclease (D) assays. Template DNA contained 100% cytosine (dC), methylcytosine (d5mC) or hydroxymethylcytosine (d5hmC) (except primer sequences). Exemplary agarose gel images for Klenow polymerase assay (A) and for T4 DNA polymerase exonuclease assay (D) and the quantification of the corresponding sum band intensities as a proxy for polymerization (A) and exonuclease activity (D), normalized to the DNA input (0 U enzyme) are shown. (**B**) Schematic representation of the experimental setup for *in vivo* nucleotide incorporation analysis. Cells were incubated with 10 μM EdU for the pulse labeling times indicated 24 h after transfection, EdU was detected and normalized nuclear EdU sum fluorescence intensities of S-phase cells are plotted. (**C**) Schematic representation of the experimental setup for *in vivo* nucleotide incorporation analysis in dCTP, d5mCTP or d5hmCTP transfected cells. Cells were incubated with 10 μM EdU for the pulse labeling times indicated 24 h after nucleotide transfection; EdU was detected and normalized nuclear EdU sum fluorescence intensities of S-phase cells are plotted. Independent experiments were done twice (A, C and D) or in triplicates (B) and *P* and *n*-values are summarized in Supplementary Table S7. Full gels are shown in Supplementary Figure S6. All boxes and whiskers are as in Figure 2; * *P* < 0.05, ** *P* < 0.005 and *** *P* < 0.001.

Besides RNA synthesis, DNA polymerization during DNA replication and repair is an essential nuclear process to ensure complete and error-free genome duplication. We, thus, aimed to investigate the effect of DNA base modifications on DNA polymerase activity. To test this, we performed *in vitro* DNA replication assays using *in vitro* modified DNA templates and *in vitro* purified Klenow or Taq DNA polymerases. The function of DNA polymerases in DNA synthesis is conserved across species and the basic reaction mechanism of DNA double helix opening does not differ between prokaryotic and eukaryotic organisms (73,74). Modified dsDNA template, dNTPs and primers were heated to denature DNA, cooled down to allow primer annealing and incubated at 37 or 72°C while adding Klenow or Taq polymerase, respectively. Strand displacement and DNA polymerization were analyzed by agarose gel electrophoresis. The amount of DNA synthesized, increased with increasing polymerase concentrations, and d5mC modified templates resulted in less DNA amplification compared to d5hmC and unmodified cytosine (Figure 5A; Supplementary Figure S14A and Supplementary Tables S7 and S8). These results indicate that modified cytosines influence the *in vitro* DNA polymerization rate in a way that correlates with the helix stabilization effect of the modified nucleotide present in the template DNA. Similarly to RNA polymerases, several archaeal (KOD), bacterial (Klenow exo^−^) and human (polymerase δ and η) DNA-dependent DNA polymerases were also shown to discriminate barely, if any, between dGTP incorporation opposite unmodified and modified cytosines and very similar dGTP incorporation rates were found for C, d5mC, d5hmC and d5fC (75–78). Consequently, our results most probably reflect differing helix stability rather than different polymerase specificities or elongation effects.

To extend our *in vitro* enzymatic assay results to an eukaryotic system, we performed processive soluble replication reactions with the purified *Saccharomyces cerevisiae* replisome components that are required for replication *in vitro* as described in (42). Double-stranded 3 kb linear template DNA contained an unmodified central ARS306 yeast origin of replication sequence with two opposing ORC-binding sites. This allows loading of an MCM helicase double hexamer and gives rise to bidirectional replication. DNA templates additionally contained 3′ and 5′ (un)modified sequences flanking the origin (Supplementary Figure S15A). To further analyze the influence of different amounts of modified cytosines and since 100% modified templates are unlikely to appear at high frequencies in cells, we included DNA templates containing various amounts of d5mC or d5hmC. To this end, PCR reactions contained either 100% (un)modified dCTPs, 50% modified and 50% dCTPs or 12.5% modified and 87.5% unmodified dCTPs (Supplementary Figure S15A). Indeed, the slot blot analysis revealed a clear decrease in signal intensity correlating with the decrease in the amounts of modified nucleotides present in the PCR reaction (Supplementary Figure S15A). *In vitro* replication assays contained replisome components enabling leading and lagging strand synthesis at comparable rates of *in vivo* replication (42), and synthesized DNA was visualized and quantified by detecting the incorporated radiolabeled nucleotides, as a proxy for DNA synthesis rates (Supplementary Figure S15A). While the yeast-based assay is no longer a single enzyme experiment, the use of purified proteins, yet, allows a very defined molecular composition of the reaction. Similarly to our results obtained with bacterial enzymes described above (Figure 5A and Supplementary Figure S14A), we observed less DNA replication for methylated templates compared to unmodified or hydroxymethylated templates (Supplementary Figure S15A). This effect was gradually reverted with decreasing d5mC levels in the template DNA. Interestingly, replication of 100% d5hmC-containing DNA was also lower, and reducing d5hmC amounts in the templates led to DNA replication rates similar to the unmodified dC template. The reversal of the d5hmC replication inhibition by decreasing modified nucleotide amounts was more pronounced than for the d5mC samples. Although we also measured this effect in the single enzyme reactions (Figure 5A), the difference between dC and d5hmC was much smaller than in the multi-complex replisome experiment (Supplementary Figure S15A). As mentioned above for the yeast RNA polymerase assay, the effects measured denote a high conservation of the enzymatic machinery involved in genome metabolism across species even the ones, such as budding yeast, with no natural DNA modifications.

We next tested the impact of cytosine modification on mammalian DNA replication *in vivo*. As it is not feasible to study DNA polymerization in living cells in the absence of DNA unwinding by the DNA helicase complex, we extended our analysis to the whole replisome, thereby analyzing the effect of d5hmC on the activity of the coupled helicase and polymerase complex. We incubated C2C12 cells 24 h after transfection for defined time periods with the thymidine analog EdU (5-ethynyl-2′-deoxyuridine) that is incorporated into newly synthesized DNA and can be detected via Click chemistry to visualize sites of ongoing DNA replication. We found EdU levels to increase with increasing labeling time (Figure 5B; Supplementary Figure S14B, Supplementary Tables S7 and S8). Low Tet1CD transfected cells incorporated more EdU in comparison to mcherry transfected cells during all three labeling times. However, when analyzing high Tet1CD expressing cells, we observed significantly decreasing levels of EdU incorporation (Supplementary Figure S15B and Supplementary Table S8). This observation is similar to the trend observed in the yeast *in vitro* replication experiments (Supplementary Figure S15A). Previous studies showed that DNA decondensation upon TSA induced histone hyperacetylation leads to slower DNA synthesis rates (38). Low Tet1CD-expressing cells showed DNA decondensation (Supplementary Figure S10C and Supplementary Table S8); however, instead of lower EdU incorporation they showed higher EdU incorporation rates (Figure 5B; Supplementary Figure S14B, Supplementary Tables S7 and S8). This indicates that the increased d5hmC levels and the accompanying less stable DNA double helix rather than DNA decondensation influence the speed of the replisome resulting in a higher nucleotide incorporation rate.

As high DNA methylation levels are a hallmark of pericentric heterochromatin and simultaneously constitute a Tet1 oxidation substrate, we further analyzed EdU incorporation at major satellite repeats. In agreement with our previous findings, we found higher incorporation of EdU in Tet1CD-transfected cells (Supplementary Figure S14B and Supplementary Table S8).

Next, we increased modified cytosine levels in the genomic DNA by d5mCTP or d5hmCTP transfection. Whereas d5mCTP incorporation led to a significantly decreased EdU signal, d5hmCTP resulted in higher EdU incorporation rates (Figure 5C and Supplementary Table S7). This is consistent with the Tet1 overexpression data and the decrease/increase of EdU incorporation reflects the DNA helix stabilizing/destabilizing effect of d5mC/d5hmC, respectively.

Lastly, we expanded the analysis to test whether and how DNA base modifications in the template DNA affect other enzymatic activities of DNA polymerases. Hence, we incubated MINX PCR amplicons with T4 DNA polymerase. In the absence of nucleotides, T4 DNA polymerase, similar to other DNA polymerases, exhibits 3′→5′ exonuclease activity. Indeed, we observed decreasing DNA amounts with increasing enzyme amounts for all three DNA templates. Exonuclease activity was, however, reduced on methylated templates (Figure 5D and Supplementary Table S7).

Taken together, these results show that DNA replication is negatively influenced by methylcytosine, whereas the oxidized methylcytosine variants revert and increase DNA polymerization speed in a dose-dependent manner.

### DNA base modifications affect DNA helicase unwinding in cells

A prerequisite for DNA replication is DNA double helix unwinding by DNA helicases into two single strands (79,80). We reasoned that changes in the mechanical stability of the DNA double strand might influence helicase speed and processivity and, thus, analyzed helicase unwinding *in vivo*.

**Figure 6.**
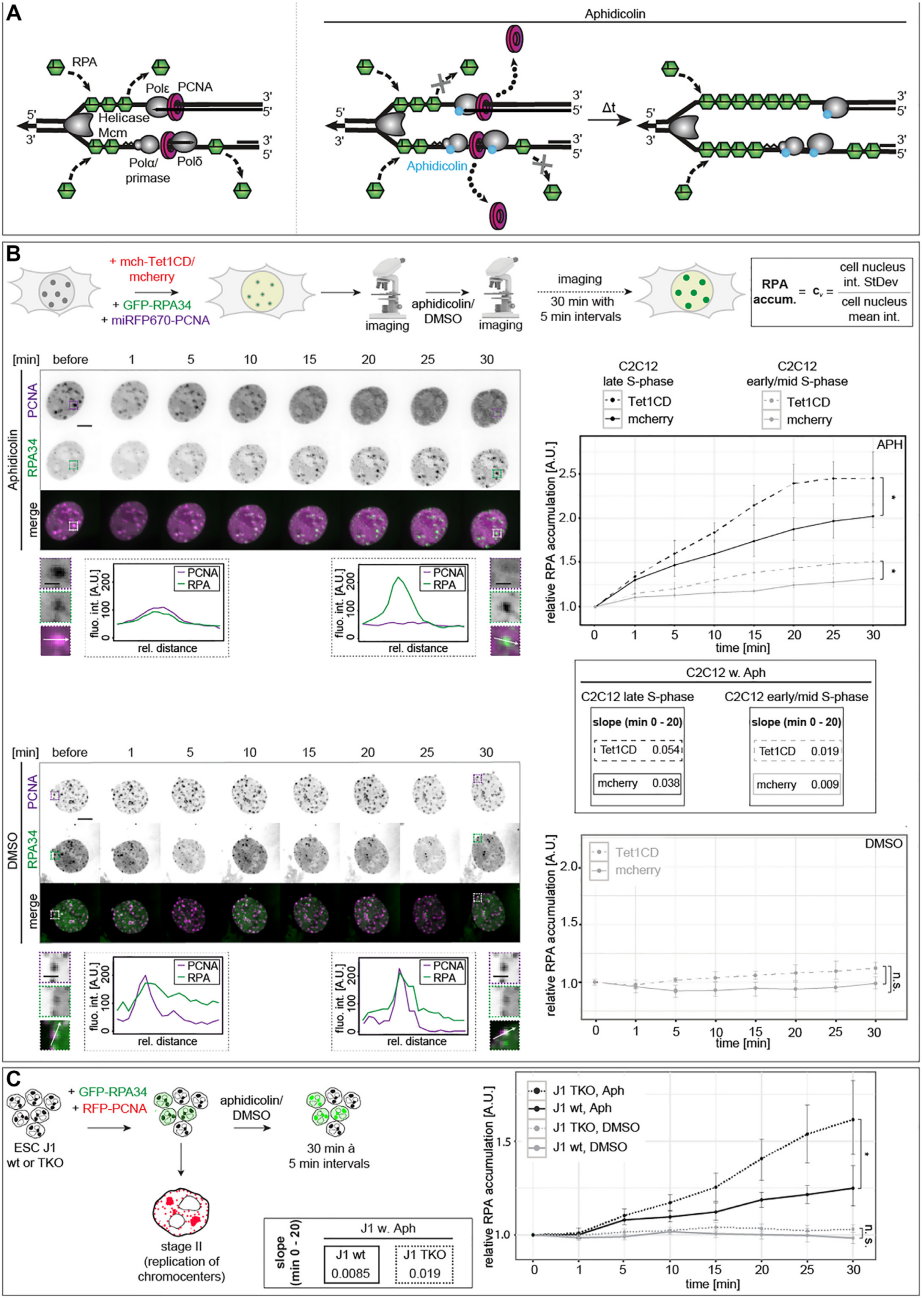
Effect of cytosine modifications on DNA helicase activity *in vivo*. (**A**) In the absence of aphidicolin, the DNA double helix is unwound by DNA helicases, ssDNA is covered with ssDNA binding proteins (RPA) and the DNA synthesis complex displaces RPA during DNA polymerization. Upon addition of aphidicolin, DNA polymerase, but not DNA helicase activity, is inhibited. This allows for continued RPA loading on the unwound ssDNA and the lack of RPA displacement by the DNA polymerases, leading to RPA accumulation at DNA replication sites. (**B**) Live cell microscopy setup for helicase activity measurements. Cells were triple transfected as indicated 24 h before imaging. RPA accumulation at replication foci, as a proxy for helicase speed, was calculated as depicted and normalized to the respective pretreatment control. Representative spinning disk confocal time-lapse microscopy images of aphidicolin/DMSO-treated cells over 30 min, showing RPA accumulation and PCNA dissociation from replication foci and the progression of S-phase, respectively (left). Dashed boxes represent the selected magnified ROIs for line intensity profile analysis (arrows) before and 30 min after aphidicolin/DMSO addition. Line intensity plots of PCNA (magenta) and RPA (green) through a replicating chromocenter before and 30 min after aphidicolin/DMSO addition are shown. Graphs showing the normalized average RPA accumulation (*c*_ν_ ± standard deviation) over 30 min at 5 min interval imaging for aphidicolin-treated cells and for 30 min imaging for DMSO-treated cells (bottom). Aphidicolin cells were grouped into early/mid and late C2C12 S-phase cells. Slopes of the RPA accumulation curves were calculated for aphidicolin treated cells as a ratio of the rise (difference of the *y*-coordinates) over the run (difference of the *x*-coordinates) of the respective linear regression line of the average RPA accumulation curves between 0 and 20 min of drug treatment. (**C**) J1 wt and TKO cells were double transfected as indicated 24 h before imaging stage II S-phase cells (chromocenter replication). Imaging, analysis and representation were performed as described above; scale bars: 5 μm (whole nuclei images) and 2.5 μm (magnifications). *P* and *n*-values are summarized in Supplementary Table S7; *P < 0.05, ***P < 0.001, n.s.: non-significant.

To test the influence of modified cytosine bases on the helicase unwinding activity *in vivo*, we made use of a system we developed before, based on the decoupling of the replisome via a drug treatment (81). For this purpose, we treated cells with the tetracyclic antibiotic aphidicolin that reversibly inhibits eukaryotic DNA polymerases α, δ and ϵ (82–85). For DNA replication to take place, the DNA double helix needs to be unwound by DNA helicases. The single-stranded DNA generated (ssDNA) is immediately covered with single-stranded DNA-binding proteins (RPA complex) to avoid DNA damage and/or re-annealing of the two DNA strands. The DNA synthesis complex, comprising DNA polymerases and PCNA (proliferating nuclear antigen), uses the ssDNA strands as template to synthesize a complementary daughter strand, while simultaneously displacing RPA from the ssDNA (Figure 6A and (80)). With the aphidicolin treatment, we specifically inhibit the DNA polymerase without inhibiting the helicase activity (81). As previously published (81), the DNA polymerase gets uncoupled from the DNA helicase, leading to a continuous unwinding of the DNA double helix by the helicase, while the DNA polymerization is stalled by aphidicolin. The ssDNA generated is continually covered with the single-stranded binding protein RPA. Due to the inhibition of the DNA polymerase, RPA is not displaced from the DNA, leading to an accumulation at DNA replication sites. Furthermore, the polymerase inhibition via aphidicolin leads to the disassembly of proteins involved in DNA replication elongation from replication sites (e.g., PCNA, Figure 6A and (81)). We made use of the uncoupling of helicase and polymerase to analyze the effect of modified cytosine bases and the concomitant change in double helix stability on the helicase unwinding speed, by measuring the RPA accumulation on ssDNA at DNA replication sites at defined time points after aphidicolin addition. C2C12 mouse myoblast cells were transiently transfected with expression constructs coding for fluorescently marked proteins, namely GFP-tagged RPA34 to determine the helicase unwinding speed via RPA accumulation over time, miRFP670-tagged PCNA to control for complete polymerase inhibition, indicated by dissociation of PCNA from replication foci and mcherry-tagged Tet1CD or mcherry. Twenty-four hours after transfection, cells were subjected to live cell confocal microscopy and only cells showing a clear S-phase pattern in the GFP channel (RPA) and/or miRFP670 (PCNA) channel were taken into consideration. As a pretreatment control, z-stacks of the cells were acquired before adding aphidicolin or DMSO and after the addition of aphidicolin (DMSO for the controls), cells were imaged every 5 min over a period of 30 min (Figure 6B). Already after 5 min of drug treatment, a clear accumulation of RPA was observed at sites of ongoing DNA replication (Figure 6B). Complete inhibition of the polymerase by aphidicolin was, on the one hand, verified by PCNA dissociation from replication foci (Figure 6B) and, on the other hand, by incubating the cells with the thymidine analog BrdU (bromodeoxyuridine) after 30 min of aphidicolin treatment, with no BrdU incorporation being detected (Supplementary Figure S16A). Control cells that were treated with DMSO, neither showed RPA accumulation nor PCNA dissociation from the replisome and incubation of the cells with BrdU showed replication foci pattern colocalizing with the RPA signal (Supplementary Figure S16A). Comparison of the RPA and PCNA signals before and after DMSO treatment showed S-phase progression during the 30 min of imaging (Figure 6B). These results demonstrate that we were able to completely block DNA synthesis in S-phase C2C12 mouse myoblast cells by aphidicolin treatment.

To measure and analyze the RPA accumulation, the nuclei of S-phase cells were segmented, the GFP intensity inside each nucleus was measured for each time point (Supplementary Figure S5), normalized to the GFP channel values at time point 0 (pretreatment) and the coefficient of variation of the GFP-channel intensities was plotted for each time point. In addition, we validated that the GFP-RPA levels did not vary significantly between the mcherry-tagged Tet1CD and mcherry expressing cells (Supplementary Figure S16B and Supplementary Table S8).

DNA replication during S-phase is organized in a distinct spatio-temporal manner that, in somatic cells, follows the chromatin compaction state, starting with replication of euchromatin (early S-phase), facultative heterochromatin (mid S-phase) and constitutive heterochromatin (late S-phase) (Supplementary Figure S1C and reviewed in (80)). The latter type of chromatin is mainly transcriptionally inactive and marked by high levels of cytosine methylation, and, in mouse cells, forms prominent clusters visible as bright foci in DAPI counterstaining. Immunofluorescent detection of d5mC and d5hmC in mcherry-transfected cells showed low 5hmC levels, which is consistent with the low Tet1-3 expression levels in C2C12 cells (86). 5mC signals were mostly, but not exclusively, visible as clustered and compact structures corresponding to chromocenters. In Tet1CD-transfected cells, 5hmC levels increased significantly and correlated with 5mC signals (Figure 2A and Supplementary Figure S17A). Highest 5hmC increases were observed in the normally highly methylated constitutive heterochromatic genomic regions. Additionally, colocalization analyses via line intensity profiles and Pearson’s correlation factors showed the strict dependency of 5hmC on 5mC (Supplementary Figure S17A). According to the genome-wide analyses of Jin *et al.* (56), Tet1CD catalytic activity is not biased to specific methylated genomic regions and can act throughout the genome. Hence, 5hmC generation and distribution ‘follow’ 5mC genome-wide. Considering the fundamental prerequisite of the presence of 5mC to allow 5hmC generation, hydroxymethylation is prominently observed in pericentromeric heterochromatin (chromocenters) of Tet1CD-transfected cells.

In view of the abundant chromocentric 5hmC levels in Tet1CD-transfected cells, we analyzed RPA accumulation in the helicase unwinding assays in late S-phase cells, i.e. the time of S-phase when chromocenters are replicated, and early/mid S-phase cells separately (Figure 6B). Correlation analyses revealed RPA accumulation at these highly modified regions (Supplementary Figure S17B). To confirm the presence of 5hmC in pericentromeric heterochromatin in GFP-RPA and mcherry-Tet1CD transfected cells treated with aphidicolin/DMSO, we co-stained the latter for 5mC and 5hmC and performed line profile colocalization analyses (Supplementary Figure S17B). Late S-phase cells depicted clear GFP-RPA accumulation at 5mC-stained genomic regions, a hallmark for pericentromeric heterochromatin. As previously published (81), aphidicolin treatment led to the accumulation of GFP-RPA at sites of ongoing DNA replication. Tet1CD-transfected cells additionally showed clear 5hmC signals, which, due to the strict prerequisite of 5mC for 5hmC oxidation, overlap with the immunofluorescently detected 5mC.

Detection and analysis of the helicase activity and speed in replicating C2C12 cells showed a time-dependent accumulation of RPA at replication sites of S-phase cells treated with aphidicolin (Figure 6B; Supplementary Figure S16C, Supplementary Tables S7 and S8). Cells transfected with Tet1CD, however, showed a significantly higher amount of RPA accumulation at replication sites than mcherry transfected cells. Potential reduction of DNA helicase activity as a consequence of aphidicolin addition would be similar in both cases, thus, allowing us to conclude that the effects observed are Tet1CD activity dependent. Interestingly, increased RPA accumulation in Tet1CD-expressing cells was observed for early and mid S-phase substages; late S-phase cells, however, showed a higher accumulation after 30 min of drug treatment (Figure 6B).

As the main increase of RPA accumulation took place during the first 10 min after aphidicolin addition, we specifically analyzed the RPA accumulation during this period by imaging the triple transfected cells at a higher frame rate with one image every 10 s for 12 min. As expected, Tet1CD-transfected cells showed a rapid and steady increase of RPA accumulation, whereas a much slower RPA accumulation was observed for the control cells (Supplementary Figure S16C). No RPA accumulation could be measured for DMSO control-treated cells, although Tet1CD-transfected cells showed slightly higher RPA levels after 30 min (Figure 6B). Similar results of increased RPA accumulation in Tet1CD transfected cells were obtained when the replisome uncoupling was performed in triple transfected MEF W8 cells (Supplementary Figure S16D and E and Supplementary Table S8).

To further validate the effect of d5mC on the DNA unwinding activity, we used the reverse cellular model of loss of cytosine modification by deletion of all three DNA methyltransferases (Dnmt1, Dnmt3a and Dnmt3b, [TKO]) in ES cells or the maintenance DNA methyltransferase Dnmt1 in fibroblasts. We, therefore, co-transfected J1 wt and J1 TKO ES cells and MEF W8 (wild-type) and MEF *dnmt1^−^^/^^−^* (PM) fibroblasts with GFP-RPA and RFP-PCNA expression constructs to identify S-phase cells. Akin to the results we obtained with overexpression experiments, we observed in wild-type and knockout cells a time-dependent RPA accumulation, PCNA dissociation from replication foci and the lack of BrdU incorporation after aphidicolin addition. RPA accumulation was higher in J1 TKO and MEF PM cells compared to wild-type J1 and MEF W8 cells. DMSO-treated cells showed normal S-phase progression, BrdU incorporation in newly synthesized DNA as well as no significant RPA accumulation (Figure 6C and Supplementary Figure S18A–C). These results are in line with increased helicase unwinding in d5mC-deficient cells, which is associated with a decrease in helix stability and more efficient helicase unwinding.

As Tet1 overexpression as well as Dnmt1 deletion could deeply impact chromatin structure, which in turn might influence genomic processes like DNA unwinding prior to DNA replication, we investigated potential changes in chromatin accessibility and nucleosome composition by micrococcal nuclease (MNase) digestion assay. We found that the ratio of monomeric nucleosomal DNA to the total DNA for each MNase concentration did not vary significantly (Supplementary Figure S19A), indicating that nucleosome positioning and number were not significantly influenced by Tet1CD overexpression. Similar results were obtained for MEF W8 and MEF PM cells (Supplementary Figure S19B).

In summary, methylated DNA is unwound by helicases slower than unmodified DNA, whereas oxidation of the methyl group reverts this effect and even enhances helicase unwinding, at least in part due to its DNA duplex destabilizing effect. Accordingly, we show that the lack of methylated cytosines and consequently the less stable DNA helix, also increases the unwinding speed of DNA helicases *in vivo*. This is in agreement with our previous results that the addition of a methyl group to cytosine bases leads to a more stable DNA helix and in turn higher energy and/or more time is needed to separate the helix in two single-stranded DNA strands.

### Loss of DNA methylation affects S-phase progression and increases replication fork speed in mouse embryonic stem cells

**Figure 7.**
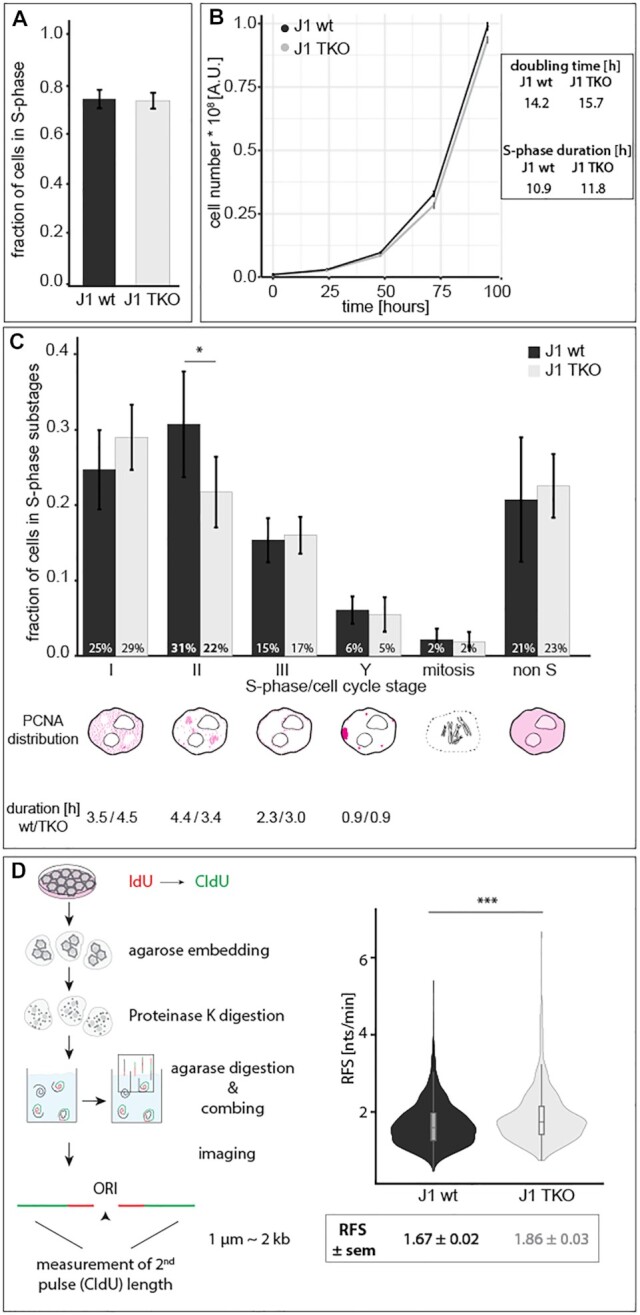
Effect of loss of cytosine modifications in mouse embryonic stem cells on S-phase progression and replication fork speed. (**A**) Bar plot showing the mean percentage of cells in S-phase in J1 wt and TKO ES cells. (**B**) Line plots showing the population doubling time of mouse J1 and TKO ES cells over five consecutive days with the ensuing doubling times depicted. S-phase duration was derived from the doubling times and the percentage of S-phase cells. (**C**) Bar plot showing the mean percentage of cells within the different cell cycle and S-phase substages (S-phase I - Y, G1/G2 (non S) and mitosis) and the ensuing S-phase substage durations. (**D**) Schematic representation of the molecular combing technique. Violin plots showing the replication fork speed (RFS) of J1 wt and TKO cells. Error bars represent the standard deviation, all boxes and whiskers are as in Figure 2 and independent experiments were performed in quadruplicates (A**–**C) or in duplicates (D). *P* (determined by Mann–Whitney–Wilcoxon test) and n-values are summarized in Supplementary Table S7; * *P* < 0.05 and *** *P* < 0.001.

Since helicase-mediated DNA unwinding and DNA polymerization are the key enzymatic processes during genome duplication and in view of our results described above, we aimed to further clarify the effect of loss of DNA methylation on cell cycle and S-phase progression in general as well as DNA replication at the molecular level. Loss of all three DNA methyltransferases completely abolished d5mC levels in J1 TKO cells (Figure 2E and Supplementary Figure S9C). Doubling time and the percentage of cells present in S-phase of J1 TKO was not different compared to wild-type ES cells (Figure 7A and B; Supplementary Table S7). Importantly, loss of d5mC did not change the temporal order of DNA replication patterns of ES cells ((39) and Supplementary Figure S3). However, when we compared the frequency of the different replication substages in J1 wt and TKO cells, we observed a significant decrease of TKO cells at stage II (Figure 7C and Supplementary Table S7), indicating that duplication of normally highly methylated pericentromeric heterochromatin required less time in TKO than in wild-type cells (39). In general, faster S-phase progression can be achieved either by the activation of additional origins of replication or by increasing the replication fork speed (RFS). We, thus, measured RFS of J1 wt and J1 DNMT TKO cells by molecular combing. ES cells lacking global DNA methylation exhibited a significant increase in DNA replication fork speed (Figure 7D and Supplementary Table S7).

Previous studies in Dnmt1-deficient fibroblasts showed that the loss of DNA methylation was accompanied by an increase in histone acetylation, chromatin decondensation and reduced replication fork speed (38). In ES cells, however, no significant differences in histone acetylation or methylation were observed between wild-type and knockout cells (Supplementary Figure S20, Supplementary Table S8 and (57)), arguing against a possible effect of histone modifications on the observed differences in RFS. Furthermore, in somatic cells increased histone acetylation resulted in decreased fork speed, which is opposite to what we found here. All in all, in asynchronous J1 Dnmt TKO cell cultures, we counted fewer stage II cells (∼10%) and concomitantly calculated a shorter stage II duration, indicating that Dnmt TKO cells require less time for the duplication of pericentromeric heterochromatin. Together with the difference in replication fork speed (∼10%), our results suggest that the loss of DNA methylation results in faster replication fork progression, specifically along DNA that is usually highly methylated under wild-type conditions.

## DISCUSSION

DNA base modifications greatly expand genome diversity and provide additional mechanisms to regulate gene expression. Consequently, the tight regulation of their establishment, maintenance and removal is essential for proper cell survival and development. In mammalian cells, the most extensively studied DNA modification is methylation of cytosines by Dnmts (87). More recently, the Tet enzyme family was found to oxidize d5mC (8,9) and the function and regulation thereof are still poorly understood. Whereas DNA methylation has been mainly linked to gene silencing and chromatin remodeling (88), Tet-mediated oxidation of d5mC is, on the one hand, a mechanism for active loss of DNA methylation (10) and, on the other hand, the oxidized derivatives of d5mC are reported to be stably maintained in the genome (89,90). Specific readers of all cytosine base modifications have been reported (14,91) and their influence on gene expression and chromatin regulation is the subject of extensive investigations. Whether and how cytosine base modifications can however directly influence the DNA double helix and, as a consequence, fundamental genomic processes such as DNA replication or transcription are far less understood (reviewed in (17)).

**Figure 8.**
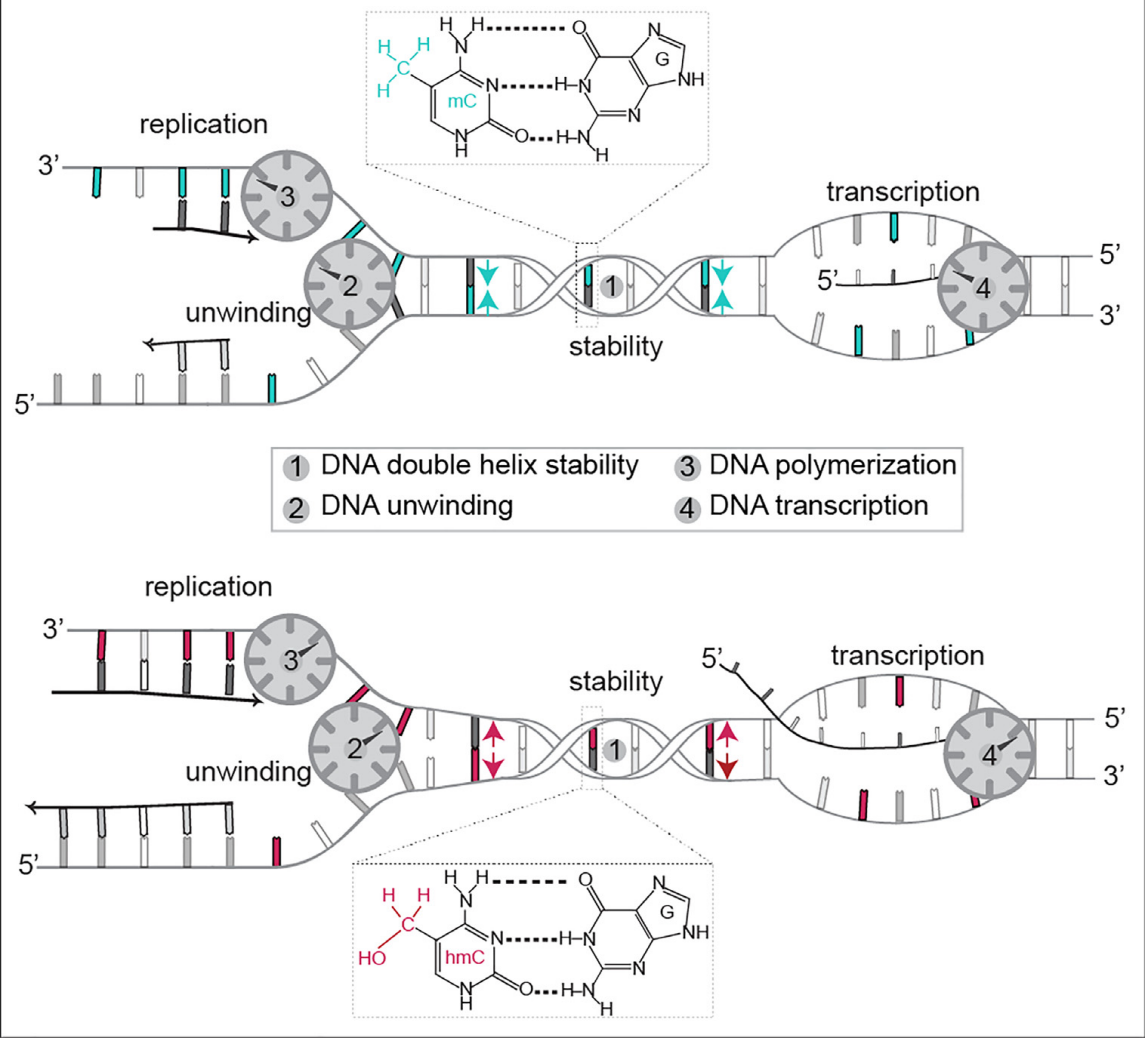
Graphical summary of the effect of cytosine modifications on DNA double helix stability and metabolism. DNA methylation (blue) increases DNA duplex stability (1) and reduces in turn helicase (2) and DNA/RNA polymerase (3/4) speed. Oxidation of methylated cytosine to hydroxymethylation (red) results in decreased helix stability (1) and increased helicase (2) as well as DNA/RNA polymerase (3 and 4) speed.

Here, we show *in vitro* and *in vivo* that the presence of methylated cytosines increases the melting temperature of the DNA double helix, demonstrating that the addition of the methyl group stabilizes the double helix structure. Incorporation of oxidized variants of d5mC, however, leads to lower melting temperatures, hinting toward a helix destabilizing effect of the hydroxy, formyl and carboxyl groups (Figure 8). Previous *in vitro* studies showed increased sequence dependent duplex stability upon cytosine methylation (20,25,50,55). Additionally, calculations of enthalpy and entropy changes upon cytosine modifications also reflected the thermal stability effects measured (92). Analysis of hydrogen bonding and base stacking interactions reinforced the idea of an increase in stability upon cytosine methylation and a destabilizing effect arising from hydroxymethyl, formyl and carboxyl groups (28,93,94). The influence on base stacking would to some extent also explain the previously observed sequence dependency. While increasing the size of the cytosine modification leads to less structural fluctuation between base pairs and, thus, a more rigid and stable structure, this can be counteracted by adding polar groups as in hydroxymethylcytosine. This is explained by the fact that the hydrophobic methyl group is less favorable for interaction with water molecules, resulting in a higher rigidity and, consequently, altogether in higher thermostability of 5mC-G base pairs, whereas the more hydrophilic and polar hydroxymethyl group has higher affinity for water molecules, leading to higher fluctuations in 5hmC-G base pairs (28). Furthermore, electron-withdrawing abilities of the formyl and carboxyl groups reduce hydrogen bonding in 5fC-G and 5caC-G base pairs, destabilizing the DNA duplex (93). These *in vitro* and *in silico* data are in line with our findings and provide a mechanistic explanation for our results.

The local changes in DNA helix structure may also represent a specific recognition platform for reader proteins. The weaker d5fC and d5caC pairing abilities could facilitate TDG base flipping during DNA demethylation (93). In addition, it was shown that DNase I cleavage was enhanced adjacent to methylated CpGs, presumably due to the minor groove narrowing effect exerted by the bulky methyl group (95). DNA shape variations arising from the presence of methylated DNA bases augment the negative electrostatic potential, which in turn can more effectively attract basic side chains of DNA-binding proteins (95–97). Interestingly, the chromatin remodeler proteins MeCP2, Mbd1 and Mbd2 identify 5mCpGs via two conserved positively charged, arginine-rich fingers (98–100), possibly exploiting changes in helix structure and stability for binding site recognition.

In our *in vivo* analyses, we made use of several different approaches to manipulate cytosine modification levels within cells (Supplementary Figure S9). To increase modified cytosine levels, on the one hand, we changed expression levels of enzymes modifying cytosines (Dnmts and Tet1; Supplementary Figure S9A and B). On the other hand, we chose a nucleotide-based approach (Supplementary Figure S9C). Both approaches were performed in undifferentiated C2C12 mouse myoblast cells, as these cells show low endogenous Tet1–3 and Mbd expression (15,86,101). The lack of the N-terminal CXXC DNA-binding motif in the Tet1CD variant allowed us to achieve global, random and unbiased methylcytosine oxidation. In contrast to Tet1 full-length (Tet1fl), Tet1CD has no preference for specific genomic regions and massive conversion of DNA methylation is observed in CpG islands (CGIs), non-CGIs, upstream, promoter, exon, intron, downstream and intergenic DNA regions (56). To study the loss of cytosine modifications on DNA structure and subsequent genomic processes, we used Dnmt1/Dnmt3a/Dnmt3b knockout mouse embryonic stem cells (57) and Dnmt1 knockout mouse embryonic fibroblasts (35), both of which are depleted of cytosine modifications (Supplementary Figure S9B).

We show that DNA helicase, DNA exonuclease and DNA-dependent DNA and RNA polymerase speed are regulated by the cytosine variants present in the DNA templates (Figure 8). In agreement with the duplex stabilizing effect of cytosine methylation observed in our *in vitro* melting experiments, helicase and polymerase speeds were decreased with increasing d5mC level in cells. Importantly, DNA replication initiation is not dependent on the presence of cytosine variants, as Dnmt knockout cells actively replicate their genome within a similar time frame to the wild-type cells. Hence, cytosine variants are unlikely to influence the amount of active replicons. In contrast, oxidation of d5mC to d5hmC (and d5fC or d5caC) resulted in helix destabilizing effects and increased *in vivo* helicase and polymerase speed/activity. Interestingly, this process is not dependent on chromatin decondensation or global nucleosomal remodeling effects. Oxidation of cytosine methylation therefore *de facto* reverts DNA duplex stability without the actual need for removal of the methyl group by DNA repair-based processes. It is thus tempting to speculate that cytosine modifications could locally regulate DNA unwinding, DNA replication and gene expression by changing DNA (electro)mechanical properties. This is further corroborated and expanded by our replication fork speed analysis in TKO ES cells (Figure 7D). Importantly, the effect we measured was greater in normally heavily methylated genomic regions (Figures 6B and C, 7C; Supplementary Figure S17A). Since we previously mapped the exact positions of methylated CpGs in major satellite repeats and in view of the unbiased and global cytosine oxidation of Tet1CD, this observation underlines the decrease in DNA helix stability by the loss of cytosine methylation. In contrast to results from somatic cells (38), we did not observe changes in histone acetylation upon Dnmt and concomitant d5mC depletion, indicating that the increased fork speed was dependent on DNA helix stability rather than on chromatin decondensation. In line with this, it was shown that bacteriophage T7 DNA helicase unwinding rate is correlated to the local DNA sequence and unzipping force (102). Genome-wide mapping of DNA destabilizing oxidized cytosine modifications revealed enrichment of d5hmC and d5fC within gene bodies of actively transcribed genes and within CpG islands in promoters of actively transcribed genes (103,104). Cytosine methylation, however, stabilizes the DNA helix and plays a role in transcriptional repression and gene silencing (65). This is consistent with our data that oxidation of d5mC increased the transcription rate *in vitro* and *in vivo*.

We now propose an additional role of DNA base modifications in the regulation of genome processes by directly modulating DNA double helix stability to impede (cytosine methylation) or facilitate (methylation oxidation) access and unwinding of double-stranded DNA. Hence, the variegated modifications along the DNA molecule could play a role in the specific regulations of its own local transactions.

## DATA AVAILABILITY

All data and custom written scripts are available from the OMERO open microscopy environment public repository (http://cc-omero.bio.tu-darmstadt.de/webclient/userdata/?experimenter=-1) and TUdatalib (https://doi.org/10.48328/tudatalib-551). All renewable biological materials will be made available upon request from the lead corresponding author M. Cristina Cardoso (cardoso@bio.tu-darmstadt.de).

## Supplementary Material

gkab509_Supplemental_File

## Notes

Present address: Florian D. Hastert, Paul-Ehrlich-Institut, Bundesinstitut für Impfstoffe und biomedizinische Arzneimittel, 63225 Langen, Germany.
