# Supplementary material for: Cytosine base modifications regulate DNA duplex stability and metabolism
Source: Nucleic Acids Res. 2021 Jun 16;49(22):12870–94. doi: 10.1093/nar/gkab509 (PMC8682791; doi:10.1093/nar/gkab509)
Supplement: gkab509_Supplemental_File [file gkab509_supplemental_file.pdf]

Cytosine base modifications regulate DNA duplex stability and metabolism

Cathia Rausch[1], Peng Zhang[1,2,*], Corella S. Casas-Delucchi[3,#], Julia L. Daiß[4,#], Christoph Engel[4], Gideon Coster[3], Florian D. Hastert[1], Patrick Weber[1] and M. Cristina Cardoso[1,*]

[1] Cell Biology and Epigenetics, Department of Biology, Technical University of Darmstadt, 64287 Darmstadt, Germany.
[2] Center for Tissue Engineering and Stem Cell Research, Guizhou Medical University, Guiyang, Guizhou, China, 550004.
[3] The Institute of Cancer Research, Chester Beatty Laboratories, London SW3 6JB, United Kingdom.
[4] Regensburg Center for Biochemistry, University of Regensburg, 93053 Regensburg, Germany.

[#] These authors contributed equally to this work.

* To whom correspondence should be addressed: M. Cristina Cardoso: Tel: +49 6151 16 21882; Fax: +49 6151 16 21880; Email: cardoso@bio.tu-darmstadt.de.
Correspondence may also be addressed to: Peng Zhang: Tel/Fax: +86 85180988028; Email: peng12zhang@outlook.com.

Present address: Florian D. Hastert, Paul-Ehrlich-Institut, Bundesinstitut für Impfstoffe und biomedizinische Arzneimittel, 63225 Langen, Germany.

SUPPLEMENTARY TABLES, FIGURES AND FIGURE LEGENDS

Supplementary Table 1: Oligonucleotide characteristics.

Name	Sequence [5' - 3']	Application	Reference
**AgeI**-miRFP-fw	ATC C**AC CGG T**CG CCA CCA TGG TA	cloning	this study
**BsrGI**-miRFP-rev	AAA **TGT ACA** GGC TCT CAA GCG CGG TGA T	cloning	this study
MaSat-fw	AAA ATG AGA AAC ATC CAC TTG	FISH probe generation	(1)
MaSat-rev	CCA TGA TTT TCA GTT TTC TT	FISH probe generation	(1)
CG-up	ACT ACC ATC **X**GG ACC AGA AG*	HRM	(2)
CG-down	CTT CTG GTC **X**GG ATG GTA GT*	HRM	(2)
MINX-fw	CGG TAC CTA ATA CGA CTC ACT ATA GGG AGA	PCR fragment, replication & IVT template generation	(2)
MINX-5'P-fw	5'Phospho-CGG TAC CTA ATA CGA CTC ACT ATA	5' phosphorylated IVT template generation	this study
MINX-rev	GTG CCA AGC TTG CAT GC	PCR fragment & IVT template generation	(2)
LINE1-fw	ATC CCA CAC CTG GCT CAG AGG G	PCR fragment generation	(3)
LINE1-rev	GTC AGG GGT CAG GGA CCC ACT T	PCR fragment generation	(3)
GC497	GAT CCT TAA TTA ACC TCA GCT TGA CCA TGA CTC GAC TGC AAT CGC CCT CAG CGC GGC CGC CTG CA	cloning	this study
GC498	GGC GGC CGC GCT GAG GGC GAT TGC AGT CGA GTC ATG GTC AAG CTG AGG TTA ATT AAG	cloning	this study
pGC504-3kb-fw	TTC TAA ACC ATT GCC GCT TAC TC	yeast replication template generation	this study
pGC504-3kb-rev	CCG TAT CGT AGT TAT CTA CAC GAC	yeast replication template generation	this study
MINX-400bp-tailed-fw	ATC ACC TTA CCC TAT ACT TAC TCG CAT TCC CGG TAC CTA ATA CGA CTC ACT ATA	yeast IVT template generation	this study
MINX-400bp-tailed-rev	GTG CCA AGC TTG CAT GCC TGC	yeast IVT template generation	this study
Competitor oligonucleotide	CGA GTA AGT ATA GGG TAA GGT GAT	yeast IVT template generation	(4)

* **X**: cytosine, 5-methyl, 5-hydroxymethyl, 5-formyl or 5-carboxyl cytosine.

Supplementary Table 2: Plasmid characteristics.

Name	pc number*	Fluorophore	Gene species	Promoter	Reference
mch-mTet1CD	2547	mcherry	Mus musculus	CAG	(5)
mcherry	2387	mcherry	Discosoma sp.	CMV	(5)
mRFP-hPCNA	1054	mRFP	Homo sapiens	CMV	(6)
miRFP-hPCNA	3385	miRFP670	Homo sapiens	CMV	this study
GFP-hRPA34	624	GFP	Homo sapiens	CMV	(7)
pUC18-MINX-M3	3902	-	-	T7	(8)
pGC504 ARS306	4625	-	Saccharomyces cerevisiae	-	this study

* pc number: plasmid collection number.

Supplementary Table 3: Cell line characteristics.

Name	Species	Type	Genotype	Reference
C2C12	Mus musculus	myoblast	wildtype	(9)
MEF W8	Mus musculus	embryonic fibroblast	wildtype	(10)
MEF PM	Mus musculus	embryonic fibroblast	$p53^{-/-}$ & $Dnmt1^{-/-}$	(11)
HEK 293	Homo sapiens	embryonic kidney	wildtype	(12)
J1 wt	Mus musculus	embryonic stem cell	wildtype	(13)
J1 TKO	Mus musculus	embryonic stem cell	$Dnmt1^{-/-}$ & $Dnmt3a^{-/-}$ & $Dnmt3b^{-/-}$	(14)

Supplementary Table 4: Nucleotide and nucleoside characteristics.

Name	Application	Cat #	Company
Biotin-16-dUTP	labeling of FISH probes	-	selfmade (1)
EdU (5-ethynyl-2'-deoxyuridine)	labeling of nascent DNA	E10415	Thermo Fisher Scientific, Waltham, MA, USA
BrdU (5-bromo-2'-deoxyuridine)	labeling of nascent DNA	B5002	Sigma-Aldrich, St Louis, MO, USA
Thymidine	labeling of nascent DNA (during chase time)	T9250	Sigma-Aldrich, St Louis, MO, USA
EU* (5-ethynyl-uridine)	labeling of nascent RNA	E10345	Thermo Fisher Scientific, Waltham, MA, USA
dATP, dTTP & dGTP	PCR & *in vitro* replication	10297018	Thermo Fisher Scientific, Waltham, MA, USA
dCTP	PCR, manipulation of C modification level (transfection) & *in vitro* replication	10297018	Thermo Fisher Scientific, Waltham, MA, USA
d5mCTP	PCR & manipulation of C modification level (transfection)	NU-1125	Jena Bioscience, Jena, Germany
d5hmCTP	PCR & manipulation of C modification level (transfection)	NU-932	Jena Bioscience, Jena, Germany
d5fCTP	PCR	N-2064	Trilink, San Diego, CA, USA
d5caCTP	PCR	N-2063	Trilink, San Diego, CA, USA
α^{33}P-dATP, 3000 Ci/mmol, 20 mCi/ml	*in vitro* yeast replication assay	SRF-203H	Hartmann Analytic, Braunschweig, Germany
Cy3-dUTP	labeling of nascent DNA (transfection)	PA53022	GE Healthcare, Chicago, IL, USA
ATP, UTP, CTP & GTP*	*in vitro* T7 run-off transcription & *in vitro* yeast replication	18109-017	Thermo Fisher Scientific, Waltham, MA, USA
α^{32}P-CTP*, 400 Ci/mmol, 10 mCi/ml	*in vitro* transcription assay	SRP-109	Hartmann Analytic, Braunschweig, Germany
5-iodo-2'-deoxyuridine (IdU)	molecular DNA combing	I7125	Sigma-Aldrich, St Louis, MO, USA
5-chloro-2'-deoxyuridine (CldU)	molecular DNA combing	C6891	Sigma-Aldrich, St Louis, MO, USA

* ribonucleotides for RNA synthesis.

Supplementary Table 5: Primary and secondary antibody characteristics.

Reactivity	Host	Dilution	Application	Cat #	Company/Reference
anti-PCNA (PC-10)	mouse	1:100/1:1000	IF*/WB**	M0879	Dako, Hamburg, Germany
anti-5mC (33D3)	mouse	1:150/1:1000	IF/Slot Blot	39649	Active Motif, La Hulpe, Belgium
anti-5hmC	rabbit	1:250/1:5000	IF/Slot Blot	39769	Active Motif, La Hulpe, Belgium
anti-BrdU	rabbit	1:500	IF	600-401-C29	Rockland Immunochemicals Inc., Limerick, PA, USA
anti-H3 acetyl	rabbit	1:200	IF	06-599	Upstate, Lake Placid, NY, USA
anti-H3K9ac (1B10)	mouse	1:500	IF	61251	Active Motif, La Hulpe, Belgium
anti-H3K18ac (EP959Y)	rabbit	1:200	IF	ab40888	Abcam, Cambridge, UK
anti-H3K27ac	rabbit	1:200	IF	4353	Cell Signaling Technology, Danvers, MA, USA
anti-H3K56ac (EPR996Y)	rabbit	1:100	IF	ab76307	Abcam, Cambridge, UK
anti-H4K8ac	rabbit	1:100	IF	06-760	Upstate, Lake Placid, NY, USA
anti-H4K16ac	rabbit	1:200	IF	39167	Active Motif, La Hulpe, Belgium
anti-H3K9m3	rabbit	1:200	IF	39161	Active Motif, La Hulpe, Belgium
anti-H4K20m3	rabbit	1:500	IF	ab9053	Abcam, Cambridge, UK
anti-RFP (5F8)	rat	1:10	IF/FISH	-	(15)
anti-5fC	rabbit	1:2000	Slot Blot	61223	Active Motif, La Hulpe, Belgium
anti-5caC	rabbit	1:2000	Slot Blot	61225	Active Motif, La Hulpe, Belgium
anti-BrdU/IdU (B44)	mouse	1:200	molecular combing IF	347580	Becton Dickinson, Franklin Lakes, NJ, USA
anti-BrdU/CldU (BU1/75 (ICR1))	rat	1:200	molecular combing IF	OBT003 0CX	Biorad, Puchheim, Germany
anti-ssDNA (16-19, IgG2a)	mouse	1:200	molecular combing IF	MAB303 4	Millipore, Burlington, MA, USA
anti-mouse IgG Chromeo 488	goat	1:1000	IF (fluorescent secondary)	15031	Active Motif, La Hulpe, Belgium
anti-rabbit IgG AMCA	donkey	1:00	IF (fluorescent secondary)	715-155-151	The Jackson Laboratory, Bar Harbor, ME, USA
anti-rabbit IgG Chromeo 488	goat	1:1000	IF (fluorescent secondary)	15041	Active Motif, La Hulpe, Belgium
anti-mouse IgG Cy3	donkey	1:300	IF (fluorescent secondary)	715-165-151	The Jackson Laboratory, Bar Harbor, ME, USA
anti-rat IgG Cy3	donkey	1:300	IF/FISH (fluorescent secondary)	712-165-153	The Jackson Laboratory, Bar Harbor, ME, USA
anti-rabbit IgG Cy3	donkey	1:300	IF (fluorescent secondary)	711-165-152	The Jackson Laboratory, Bar Harbor, ME, USA
anti-rabbit IgG Cy5	donkey	1:300	IF (fluorescent secondary)	711-175-152	The Jackson Laboratory, Bar Harbor, ME, USA
anti-mouse IgG HRP	sheep	1:5000	Slot blot (HRP*** conj. secondary)	NA 931V	GE Healthcare, Chicago, Il, USA
anti-rabbit IgG HRP	goat	1:5000	Slot blot (HRP conj. secondary)	A0545	Sigma-Aldrich, St Louis, MO, USA
anti-mouse IgG Chromeo 546	goat	1:200	molecular combing IF (fluorescent secondary)	15033	Active Motif, La Hulpe, Belgium
anti-rat IgG AlexaFluor 488	donkey	1:200	molecular combing IF (fluorescent secondary)	712-545-153	The Jackson Laboratory, Bar Harbor, ME, USA
anti-mouse IgG2a AlexaFluor 647	goat	1:200	molecular combing IF (fluorescent secondary)	A-21241	Fisher Scientific GmbH, Hampton, NH, USA

* IF: immunofluorescence, ** WB: western blot; *** HRP: horseradish peroxidase.

Supplementary Table 6: Imaging system characteristics.

Microscope /Company	Lasers/lamps	Filters (ex. & em. [nm])*	Objectives/ lenses	Detection system	Incubation system	Application
Ultra-View VoX spinning disk on an inverted Nikon Ti-E microscope/ PerkinElmer Life Sciences, UK	solid state diode lasers (405 nm, 488 nm, 561 nm, 640 nm)	405/488/568/ 640** 405: 415–475 488: 505–549 561: 580–650 640: 664–754	oil immersion 60x Plan-Apochromat (NA 1.45)	cooled 14-bit Hamamatsu® C9100-50 EMCCD	closed live-cell microscopy chamber (ACU control, Olympus) for time-lapse microscopy	time-lapse microscopy & confocal z-stack imaging
Operetta high content screening microscopy/ PerkinElmer Life Sciences, UK	Xenon fiber-optic light source, 300 W, 360 – 640 nm continuous spectrum	405: 360-400 & 410–480 488: 460-490 & 500–550 561: 560-580 & 590–640	20x or 40x air (0.45 NA and 0.95 NA) long WD***	14-bit Jenoptik CMOS	-	high content screening microscopy
Widefield Axiovert 200 /Zeiss, Germany	HBO100 mercury lamp	488: 473-491 & 506-534 561: 550-580 & 590-650 640: 590-650 & 663-738	oil immersion 63x Plan-Apochromat (1.4 NA)	12-bit AxioCam mRM	-	molecular combing imaging
Amersham AI600 imager/GE Healthcare, Chicago, Il, USA	UV transillumination light: 312 nm	EtBr: 312 & 585-625	large aperture f/0.85 FUJINON™	16-bit Peltier cooled Fujifilm Super CCD	-	EtBr stained gel & HRP stained blot imaging
Typhoon FLA-9500/GE Healthcare, Chicago, Il, USA	635 nm (red LD laser)	IP BP390	-	bi-alkali photomultiplier tubes (PMT), 16-bit digitization	-	radioactive gel imaging
Personal Molecular Imager™ System/ Biorad, Hercules, CA, USA	635 nm	BP390	-	photomultiplier tubes (PMT), 16-bit digitilization	-	radioactive gel imaging

*ex.: excitation & em.: emission, ** dichroic specification, *** WD: working distance.

Supplementary Table 7: Plot statistics (main figures).

Figure	Sample	n[a]	Median	Mean	StDev[a]	95% CI[a]	*p*-value[a]
2A	Tet1CD 1	3318	0.93	1.04	0.51	1.04 - 1.06	5.02E-05
	Tet1CD 2	1142	1.33	1.92	1.47	1.83 - 2.01	< 2.2E-16
	Tet1CD 3	128	2.61	3.21	1.3	2.98 - 3.44	< 2.2E-16
	mcherry 1	3292	1	1.1	0.54	1.08 - 1.12	0.652
	mcherry 2	2809	1.04	1.28	0.97	1.24 - 1.32	0.06399
	mcherry 3	295	0.98	1.17	0.99	1.06 - 1.28	0.6548
	untransf.	4460	1	1.07	0.66	1.05 - 1.09	-
2B	dCTP 5mC	2710	1	1.19	0.35	0.98 - 1.1	-
	d5mCTP 5mC	2274	3.3	3.13	0.41	2.98 - 3.01	0.045
	dCTP 5hmC	2564	1	1.08	0.31	0.97 - 1.0	-
	d5hmCTP 5hmC	2283	3.09	3.25	0.5	3.0 - 3.2	0.039
	dCTP 5mC	2710	1	1.06	0.29	0.99 - 1.1	-
	ddH_2O 5mC	2566	0.99	1.04	0.38	0.9 - 1.1	0.351
	dCTP 5hmC	2564	1	0.98	0.48	0.99 - 1.12	-
	ddH_2O 5hmC	2081	1.01	0.99	0.42	0.95 - 1	0.471
2C	dCTP 5mC	1935	1	1.08	0.24	0.98 - 1.02	-
	d5mCTP 5mC	2051	3.43	3.42	0.39	3.39 - 3.43	0.035
	dCTP 5hmC	2146	1	1.2	0.21	1.01 - 1.21	-
	d5hmCTP 5hmC	2314	3.17	3.09	0.41	3.3 – 3.41	0.014
3A	Tet1CD 1	300	1.47	1.47	0.05	1.47 – 1.48	3.3E-06
	Tet1CD 2	1477	1.81	2.02	1.53	1.94 – 2.09	< 2.2E-16
	Tet1CD 3	809	1.96	2.28	1.4	2.18 - 2.38	< 2.2E-16
	mcherry 1	190	1	1.11	0.45	1.04 - 1.17	-
	mcherry 2	3134	1.04	1.29	4.24	1.15 - 1.44	0.251
	mcherry 3	253	1.08	1.39	1.39	1.33 - 1.44	0.0857
3B	dCTP 65°C	4783	1	1.08	0.52	1.07 - 1.1	-
	d5mCTP 65°C	4443	0.74	0.94	0.7	0.92 - 0.96	0.000214
	d5hmCTP 65°C	4401	1.18	1.24	0.4	1.23 - 1.26	< 2.2E-16
	dCTP 70°C	4329	1	1.11	0.72	1.09 – 1.13	-
	d5mCTP 70°C	4140	0.62	0.96	1.32	0.92 - 1	< 2.2E-16
	d5hmCTP 70°C	3982	1.49	1.61	0.81	1.59 – 1.64	< 2.2E-16
3C	W8 60°C	2827	1	1.08	0.32	1.07 - 1.09	-
	W8 65°C	3150	1.52	1.33	0.66	1.31 - 1.35	-
	W8 70°C	2846	2.23	3.06	1.8	2.99 - 3.13	-
	PM 60°C	3102	2.57	3.06	1.72	1.08 -1.1	8.70E-06
	PM 65°C	4572	6.93	2.55	1.02	2.52 - 2.58	< 2.2E-16
	PM 70°C	4514	2.22	7.69	2.28	7.62 - 7.76	< 2.2E-16
3D	wt 60°C	27	1	1.12	1.2	1.06 - 1.08	-
	TKO 60°C	36	1.35	1.28	0.9	1.11 - 1.15	0.06524
	wt 65°C	22	1.44	1.43	0.8	0.87 – 0.98	-
	TKO 65°C	26	2.05	1.52	1.31	1.2 – 1.35	0.0000268
	wt 70°C	23	1.55	2.08	1.4	1.26 – 1. 47	-
	TKO 70°C	22	2.59	2.19	1.25	1.24 – 1.31	< 2.2E-16
4B	Tet1CD 25 min 1	2768	1.08	1.26	0.59	1.24 - 1.28	< 2.2E-16
	Tet1CD 25 min 2	1153	1.31	1.49	0.69	1.45 - 1.53	< 2.2E-16
	Tet1CD 25 min 3	293	1.34	1.51	0.66	1.43 - 1.58	< 2.2E-16
	mcherry 25 min 1	2283	1	1.09	0.38	1.07 - 1.1	-
	mcherry 25 min 2	3687	1.06	1.19	0.53	1.17 - 1.21	0.354
	mcherry 25 min 3	1500	1.08	1.24	0.8	1.2 - 1.28	0.0215
	Tet1CD 45 min 1	2680	1.47	1.68	0.75	1.65 - 1.7	< 2.2E-16
	Tet1CD 45 min 2	751	1.73	1.99	1.09	1.91 - 2.06	< 2.2E-16
	Tet1CD 45 min 3	147	1.68	1.95	0.86	1.81 - 2.09	1.32E-8
	mcherry 45 min 1	2553	1.39	1.52	0.62	1.5 - 1.54	-
	mcherry 45 min 2	2784	1.5	1.7	0.89	1.67 - 1.74	5.20E-09
	mcherry 45 min 3	1314	1.54	1.73	0.84	1.71 - 1.8	0.0479
	Tet1CD 60 min 1	2293	1.8	1.75	1.33	2.02 – 2.1	3.05E-11
	Tet1CD 60 min 2	1343	2.24	2.06	1.68	2.49 - 2.64	< 2.2E-16
	Tet1CD 60 min 3	257	2.24	2.62	0.75	2.472- 2.83	9.79E-10
	mcherry 60 min 1	2835	1.8	1.96	0.86	1.93 - 1.99	-
	mcherry 60 min 2	2795	1.94	2.14	0.87	2.11 - 2.18	0.145
	mcherry 60 min 3	1635	1.96	2.17	0.85	2.13 - 2.21	0.0568

Figure	Sample	n[a]	Median	Mean	StDev[a]	95% CI[a]	*p*-value[a]
	Tet1CD 120 min 1	2007	2.59	2.93	1.43	2.87 - 2.99	< 2.2E-16
	Tet1CD 120 min 2	1540	3.14	3.42	1.51	3.35 - 3.49	< 2.2E-16
	Tet1CD 120 min 3	348	3.15	3.41	1.52	3.25 - 3.57	< 2.2E-16
	mcherry 120 min 1	2527	2.35	2.58	1.02	2.54 - 2.62	-
	mcherry 120 min 2	2974	2.57	2.82	1.14	2.78 - 2.86	0.00743
	mcherry 120 min 3	1682	2.56	2.85	1.15	2.79 - 2.9	0.000485
4C	dCTP	3804	1	1.05	0.84	1.02 – 1.18	-
	d5mCTP	2864	0.41	0.39	0.99	0.35 – 0.48	0.0032
	dCTP	8619	1	1.2	0.54	0.9 – 1.31	-
	d5hmCTP	9835	2.73	2.83	1.12	2.54 - 292	0.00042
5A[b]	dC 0 U	2	-	1	-	-	-
	dC 1 U	2	-	1.36	-	-	-
	d5mC 0 U	2	-	1	-	-	-
	d5mC 1 U	2	-	1.18	-	-	0.00195
	d5hmC 0 U	2	-	1	-	-	-
	d5hmC 1 U	2	-	0.95	-	-	0.16
5B	Tet1CD 10 min 1	4125	1.26	1.47	0.71	1.45 - 1.49	< 2.2E-16
	Tet1CD 10 min 2	808	1.5	1.81	1.25	1.72 - 1.9	< 2.2E-16
	Tet1CD 10 min 3	650	1.64	1.97	1.21	1.88 - 2.06	3.82E-06
	mcherry 10 min 1	1950	1	1.21	0.58	1.18 - 1.24	-
	mcherry 10 min 2	798	1.21	1.48	0.8	1.42 - 1.53	0.022
	mcherry 10 min 3	127	1.15	1.4	0.7	1.28 - 1.52	0.323
	Tet1CD 20 min 1	1258	2.27	2.46	1.25	2.39 - 2.53	< 2.2E-16
	Tet1CD 20 min 2	1444	2.4	2.75	1.62	2.67 - 2.83	< 2.2E-16
	Tet1CD 20 min 3	184	2.25	2.45	1.37	2.25 - 2.65	0.002118
	mcherry 20 min 1	948	1.95	2.13	1.09	2.06 - 2.2	-
	mcherry 20 min 2	441	1.94	2.41	1.49	2.27 - 2.55	0.0004385
	mcherry 20 min 3	850	1.98	2.33	1.25	2.25 - 2.41	0.1832
	Tet1CD 30 min 1	1308	3.61	3.77	1.96	3.66 - 3.88	< 2.2E-16
	Tet1CD 30 min 2	1479	3.65	3.86	2.31	3.74 - 3.98	2.65E-15
	Tet1CD 30 min 3	155	3.58	3.81	2.36	3.44 - 4.18	0.0004385
	mcherry 30 min 1	1216	3.32	3.35	2.01	3.24 - 3.46	-
	mcherry 30 min 2	3657	3.47	3.98	2.75	3.89 - 4.07	0.0236
	mcherry 30 min 3	578	3.55	4.08	2.92	3.84 - 4.32	5.32E-09
5C	dCTP	8285	1	1.05	0.18	0.98 – 1.1	-
	d5mCTP	4861	0.45	0.38	0.36	0.35 – 0.48	0.024
	dCTP	9921	1	1.1	0.25	1 – 1.02	-
	d5hmCTP	4727	1.37	1.42	0.56	1.35 – 1.59	0.039
5D[b]	dC 0 U	2	-	1	-	-	-
	dC 1 U	2	-	0.182	-	-	-
	d5mC 0 U	2	-	1	-	-	-
	d5mC 1 U	2	-	0.65	-	-	0.315
	d5hmC 0 U	2	-	1	-	-	-
	d5hmC 1 U	2	-	0.08	-	-	0.902
6B	Tet1CD late 30 min	18	-	2.45	0.29	-	-
	mcherry late 30 min	18	-	2.02	0.12	-	0.0478
	Tet1CD early/mid 30 min	5	-	1.51	0.1	-	-
	mcherry early/mid 30 min	7	-	1,32	0.07	-	0.0143
	Tet1CD DMSO 30 min	21	-	1.06	0.04	-	-
	mcherry DMSO 30 min	18	-	0.99	0.05	-	0.173
6C	J1 wt DMSO	13	-	0.98	0.02	-	-
	J1 TKO DMSO	11	-	1.03	0.03	-	0.25
	J1 wt Aph	17	-	1.25	0.12	-	-
	J1 TKO Aph	20	-	1.61	0.21	-	0.0136
7A	J1 wt	1512	-	0.74	0.02	-	-
	J1 TKO	1647	-	0.72	0.02	-	0.356
7C[c]	Stage I wt	368	-	0.25	0.05	-	-
	Stage II wt	458	-	0.31	0.07	-	-
	Stage III wt	239	-	0.15	0.04	-	-
	Stage Y wt	91	-	0.06	0.02	-	-
	Mitosis wt	35	-	0.02	0.01	-	-

Figure	Sample	n[a]	Median	Mean	StDev[a]	95% CI[a]	*p*-value[a]
	Non-S wt	321	-	0.21	0.08	-	-
	Stage I TKO	484	-	0.29	0.04	-	0.6502
	Stage II TKO	365	-	0.22	0.05	-	0.0126
	Stage III TKO	326	-	0.17	0.05	-	0.6521
	Stage Y TKO	90	-	0.05	0.02	-	0.8322
	Mitosis TKO	30	-	0.02	0.01	-	0.7161
	Non-S TKO	379	-	0.23	0.04	-	0.9102
7D	J1 wt	731	1.59	1.67	0.57	0.38 – 0.84	-
	J1 TKO	641	1.75	1.86	0.67	0.56 – 1.42	0.000268

[a] n: number of cells of all replicates (if not stated otherwise), StDev: standard deviation, 95% CI: 95% confidence interval, p-value: calculated as stated in figure legends and material and methods section.
[b] n numbers refer to independent PCRs analyzed and p-value is calculated against the band intensities of the dC containing experiment.
[c] p-values are calculated for wt versus TKO in every stage.

Supplementary Table 8: Plot statistics (supplementary figures).

Figure	Sample	n[a]	Median	Mean	StDev[a]	95% CI[a]	*p*-value[a]
S8C[b]	dC	2	-	1	0.05	0.55 - 1.45	-
	d5mC	2	-	0.46	0.14	-0.8- 1.72	0.02641
	d5hmC	2	-	1.35	0.37	-1.97 - 4.67	0.03969
S8E[b]	dC	3	-	87.93	0.74	86.09 - 89.77	-
	d5mC	3	-	93.72	0.25	93.1 - 94.34	0.0014
	d5hmC	3	-	86.56	0.26	85.91 - 87.21	0.046
	d5fC	3	-	83.75	0.18	83.3 - 84.2	0.03121
	d5caC	3	-	85.4	0.95	83.04 - 87.76	0.423
S8F[b]	dC	3	-	85.7	0.68	83.86 - 87.54	-
	d5mC	3	-	89.95	0.78	89.33 - 90.57	0.003652
	d5hmC	3	-	83.44	0.9	82.79 - 84.09	0.04702
	d5fC	3	-	82.62	0.55	82.17 - 83.07	0.003151
	d5caC	3	-	83.22	0.72	80.86 - 85.58	0.02314
S10A	60°C	114	1	1.33	0.61	1.22 - 1.44	-
	70°C	113	4.81	4.89	0.77	4.75 - 5.03	8.24E-07
	80°C	121	6.1	5.98	2.33	5.56 - 6.4	0.001492
S10B up	Tet1CD 65°C 1	545	2.15	2.3	0.65	2.24 -2.35	< 2.2E-16
	Tet1CD 65°C 2	2601	2.54	2.88	1.3	2.83 - 2.93	< 2.2E-16
	Tet1CD 65°C 3	732	2.72	3.17	1.76	3.04 - 3.3	< 2.2E-16
	mcherry 65°C 1	353	1	1.09	0.6	1.02 - 1.15	-
	mcherry 65°C 2	3080	1.08	1.23	0.68	1.2 - 1.25	0.417
	mcherry 65°C 3	2145	1.16	1.38	1.42	1.32 - 1.44	0.415
	Tet1CD 70°C 1	520	2.56	2.73	0.8	2.66 - 2.8	< 2.2E-16
	Tet1CD 70°C 2	2622	2.92	3.29	1.64	3.23 – 3.35	< 2.2E-16
	Tet1CD 70°C 3	693	3.11	3.65	2.32	3.48 - 3.83	< 2.2E-16
	mcherry 70°C 1	1615	1.42	1.53	1.02	1.48 - 1.58	-
	mcherry 70°C 2	2924	1.52	1.77	2.32	1.69 - 1.85	0.1144
	mcherry 70°C 3	2770	1.61	1.87	2.15	1.79 - 1.95	0.0334
S10B down	Tet1CD 60°C 1	1002	1.68	2.01	1.05	1.94 -2.08	< 2.2E-16
	Tet1CD 60°C 2	1189	1.69	1.92	1.62	1.83 - 2.01	< 2.2E-16
	Tet1CD 60°C 3	3347	1.75	2.72	2.16	2.65 - 2.79	< 2.2E-16
	mcherry 60°C 1	1043	1	1.19	0.63	1.15 - 1.23	-
	mcherry 60°C 2	2197	0.97	1.29	1.75	1.22 - 1.36	0.708
	mcherry 60°C 3	4203	1.06	1.4	1.99	1.34 - 1.46	0.2168
	Tet1CD 65°C 1	2190	2.31	2.21	0.82	2.18 - 2.24	< 2.2E-16
	Tet1CD 65°C 2	2664	2.15	2.44	1.1	2.4 - 2.48	< 2.2E-16
	Tet1CD 65°C 3	3709	2.65	3.9	2.9	3.81 - 3.99	< 2.2E-16
	mcherry 65°C 1	1010	1.02	1.11	0.65	1.07 - 1.15	-
	mcherry 65°C 2	2428	1.04	1.08	0.79	1.05 - 1.11	0.4578
	mcherry 65°C 3	3104	1.01	1.28	0.97	1.25 - 1.31	0.05469
S10C	Tet1CD 1	3318	0.48	0.41	0.14	0.41 - 0.42	< 2.2E-16
	Tet1CD 2	1142	0.5	0.45	0.17	0.44 - 0.46	< 2.2E-16
	Tet1CD 3	128	0.51	0.46	0.16	0.43 - 0.49	< 2.2E-16
	mcherry 1	3292	0.94	0.98	0.25	0.97 - 0.99	0.857
	mcherry 2	2809	0.97	1.04	0.35	1.03 - 1.05	0.7245
	mcherry 3	295	0.98	1.09	0.39	1.05 - 1.13	0.685
	untransf.	4460	1	1.03	0.33	1.02 - 1.04	-
S10D	acet. +TSA	2806	13.68	3.28	1.95	3.21 - 3.35	< 2.2E-16
	acet. -TSA	2714	1	1.05	0.56	1.03 - 1.07	-
	decond. +TSA	1342	0.39	0.42	0.32	0.4 - 0.44	< 2.2E-16
	decond. -TSA	2262	1	1.34	0.56	1.32 - 1.36	-
	5hmC +TSA	1342	1.01	1.06	0.87	1.01 - 1.11	0.5034
	5hmC -TSA	2262	1	1.03	0.63	1 - 1.06	-
	60°C +TSA	2540	0.96	1.32	0.54	1.3 - 1.34	0.554
	60°C -TSA	2351	1	1.56	0.36	1.55 - 1.57	-
	65°C +TSA	1956	1.12	1.23	0.98	1.19 - 1.27	0.079
	65°C -TSA	3976	1	1.54	1.02	1.51 - 1.57	-
	70°C +TSA	1845	1.04	1.52	0.98	1.48 - 1.56	0.208
	70°C -TSA	2630	1	1.21	0.65	1.19 - 1.23	-
S11A	Tet1CD 1	1595	1.24	1.26	1.4	1.19 - 1.33	0.01553

Figure	Sample	n[a]	Median	Mean	StDev[a]	95% CI[a]	*p*-value[a]
	Tet1CD 2	1326	1.94	2.47	1.98	2.36 - 2.58	1.14E-06
	Tet1CD 3	1550	3.54	4.47	1.6	4.39 - 4.55	1.03E-08
	mcherry 1	3688	1.26	1.44	0.8	1.41 - 1.47	0.221
	mcherry 2	1902	1.14	1.91	1.92	1.82 - 2	0.6627
	mcherry 3	1610	1.18	1.9	0.78	1.86 - 1.94	0.83
	untransf.	1950	1	1.22	1.1	1.17 - 1.27	-
S11B	60°C	129	1	1	0.01	0.99 - 1	-
	65°C	123	1.02	1.02	0.01	1.02 - 1.02	1.05E-07
	70°C	115	1.05	1.06	0.03	1.05 - 1.07	1.13E-11
	75°C	118	1.63	1.67	0.26	1.62 - 1.72	5.04E-10
	80°C	128	3.26	3.56	1.35	3.32 - 3.8	6.89E-11
S11C	Tet1CD 65°C	1116	1.38	1.32	0.03	1.31 - 1.32	2.74E-10
	mcherry 65°C	2168	1	1	0.01	0.99 - 1	-
	Tet1CD 70°C	1218	1.51	1.39	0.05	1.38 - 139	5.35E-07
	mcherry 70°C	2034	1.1	1.03	0.01	1.02 - 1.03	-
	Tet1CD 75°C	1326	2.32	2.33	0.14	2.32 - 2.33	8.84E-06
	mcherry 75°C	1929	1.36	1.19	0.12	1.18 - 1.19	-
	Tet1CD 80°C	2105	2.62	2.38	0.43	2.36 - 2.4	0.005725
	mcherry 80°C	2251	2.48	2.4	0.16	2.39 - 2.41	-
S12B	Tet1CD 60 min 1	2293	1.03	1.15	0.53	1.13 – 1.17	< 2.2E-16
	Tet1CD 60 min 2	1343	1.26	1.4	0.69	1.36 – 1.44	< 2.2E-16
	Tet1CD 60 min 3	257	1.26	1.45	0.87	1.34 – 1.55	9.58E-11
	mcherry 60 min 1	2835	1	1.08	0.4	1.06 - 1.09	-
	mcgerry60 min 2	2795	1.08	1.17	0.46	1.16 - 1.19	0.0975
	mcherry 60 min 3	1635	1.09	1.18	0.44	1.16 - 1.2	0.847
S13A[c]	dC 30 sec	12	0.08	0.1	0.02	0.04 - 0.05	-
	dC 60 sec	12	0.14	0.22	0.05	0.12 - 0.13	-
	dC 90 sec	12	0.25	0.35	0.05	0.24 - 0.25	-
	dC 120 sec	12	0.4	0.47	0.05	0.38 - 0.4	-
	dC 600 sec	12	1	1	0	0.9 - 1.0	-
	d5mC 30 sec	12	0.06	0.05	0.02	0.05 - 0.06	n.s.
	d5mC 60 sec	12	0.11	0.12	0.03	0.10 - 0.12	n.s.
	d5mC 90 sec	12	0.22	0.23	0.03	0.21 - 0.22	n.s.
	d5mC 120 sec	12	0.37	0.34	0.04	0.36 - 0.38	n.s.
	d5mC 600 sec	12	0.87	0.81	0.08	0.8 - 0.82	n.s.
	d5hmC 30 sec	12	0.06	0.03	0.03	0.06 - 0.07	n.s.
	d5hmC 60 sec	12	0.15	0.14	0.02	0.14 - 0.15	n.s.
	d5hmC 90 sec	12	0.28	0.27	0.03	0.27 - 0.29	n.s.
	d5hmC 120 sec	12	0.41	0.39	0.04	0.41 - 0.42	n.s.
	d5hmC 600 sec	12	0.97	0.92	0.08	0.96 - 0.98	n.s.
S13B	Tet1CD 1a	5768	1.08	1.26	0.59	1.2 - 1.22	< 2.2E-16
	Tet1CD 1b	1153	1.31	1.49	0.68	1.24 - 1.31	< 2.2E-16
	Tet1CD 1c	293	1.34	1.51	0.66	1.1 - 1.2	< 2.2E-16
	Tet1CD 2	159	1.38	1.49	0.69	1.01 - 1.2	5.59E-12
	Tet1CD 3	92	1.12	1.27	0.55	0.93 - 1.21	0.0017
	Tet1CD 4	213	1.1	1.25	0.6	0.81 - 0.94	0.00011
	mcherry 1a	9283	1	1.09	0.38	1.04 - 1.06	-
	mcherry 1b	4687	1.06	1.19	0.53	1.03 - 1.06	0.89
	mcherry 1c	1500	1.07	1.24	0.8	1.09 - 1.15	0.65
	mcherry 2	1020	1.14	1.32	1.23	1.02 - 1.08	0.056
	mcherry 3	315	1.07	1.17	1.06	1.04 - 1.22	0.36
	mcherry 4	409	1.08	1.21	1.28	0.9 - 1.01	0.078
S14A[b]	dC 0 U	2	-	1	-	-	-
	dC 2 U	2	-	1.49	-	-	-
	d5mC 0 U	2	-	1	-	-	-
	d5mC 2 U	2	-	0.9	-	-	0.0121
	d5hmC 0 U	2	-	1	-	-	-
	d5hmC 2 U	2	-	1.38	-	-	0.85
S14B	Tet1CD 10 min 1	4125	1.26	1.32	0.65	1.3 - 1.34	< 2.2E-16
	Tet1CD 10 min 2	808	1.46	1.67	0.98	1.6 - 1.74	< 2.2E-16
	Tet1CD 10 min 3	650	1.52	1.83	0.87	1.76 - 1.9	3.52E-06

Figure	Sample	n[a]	Median	Mean	StDev[a]	95% CI[a]	*p*-value[a]
	mcherry 10 min 1	1950	1	1.01	0.97	0.97-1.05	-
	mcherry 10 min 2	798	1.21	1.08	1.01	1.01 - 1.15	0.022
	mcherry 10 min 3	127	1.15	1.08	0.89	0.92 - 1.24	0.323
	Tet1CD 20 min 1	1258	1.97	2.32	1.04	2.26 - 2.38	< 2.2E-16
	Tet1CD 20 min 2	1444	2.02	2.1	1.02	2.05 - 2.15	< 2.2E-16
	Tet1CD 20 min 3	184	2.01	2.08	0.98	1.94 - 2.22	0.002748
	mcherry 20 min 1	948	1.87	2.03	1.02	1.96 - 2.1	-
	mcherry 20 min 2	441	1.98	2.1	1.12	2 - 2.2	0.0397
	mcherry 20 min 3	850	1.88	1.99	0.99	1.92 - 2.06	0.1531
	Tet1CD 30 min 1	1308	3.01	3.42	1.85	3.32 - 3.52	< 2.2E-16
	Tet1CD 30 min 2	1479	3.02	3.28	1.68	3.19 - 3.37	8.47E-16
	Tet1CD 30 min 3	155	3	3.18	1.45	2.95 - 3.41	0.01529
	mcherry 30 min 1	1216	2.85	3.08	1.04	3.02 - 3.14	-
	mcherry 30 min 2	3657	2.95	3.13	1.04	3.1 - 3.16	0.022
	mcherry 30 min 3	578	2.98	3.37	1.11	3.28 - 3.46	0.03161
S15A	dC	2	-	1	-	-	-
	12.5% d5hmC	2	-	0.92	-	-	
	50% d5hmC	2	-	0.81	-	-	
	100% d5hmC	2	-	0.41	-	-	
	12.5% d5mC	2	-	0.47	-	-	
	50% d5mC	2	-	0.38	-	-	
	100% d5mC	2	-	0.32	-	-	
S15B	Tet1CD 1a	1258	1.17	1.21	0.55	1.99 - 1.23	< 2.2E-16
	Tet1CD 1b	1444	1.18	1.28	0.69	1.24 - 1.31	< 2.2E-16
	Tet1CD 1c	184	1.11	1.48	0.59	1.06 - 1.24	0.0405
	Tet1CD 2	93	1.1	1.1	0.6	0.98 - 1.22	0.458
	Tet1CD 3	38	0.92	1.1	0.67	0.85 - 1.29	0.879
	Tet1CD 4	67	0.77	0.88	0.47	0.76 - 0.99	0.0047
	mcherry 1a	948	1	1.05	0.53	1.02 - 1.09	-
	mcherry 1b	441	0.97	1.05	0.57	0.99 - 1.1	0.847
	mcherry 1c	850	0.99	1.12	0.63	0.98 - 1.26	0.38
	mcherry 2	58	0.99	1.05	0.48	0.93 - 1.18	0.96
	mcherry 3	31	1.05	1.13	0.77	0.85 - 1.41	0.589
	mcherry 4	44	0.94	0.96	0.55	0.79 - 1.12	0.087
S16B	Tet1CD	35	0.98	0.98	0.03	0.97 - 0.99	0.341
	mcherry	31	1	0.99	0.06	0.97 - 1.01	-
S16C	Tet1CD 30 min	31	-	1.8	0.13	-	-
	mcherry 30 min	29	-	1.52	0.08	-	0.0489
	Tet1CD 12 min	35	-	1.33	0.09	-	-
	mcherry 12 min	30	-	1.16	0.08	-	< 2.2E-16
S16E	MEF Tet1CD 30 min	11	-	1.71	0.09	-	-
	MEF mcherry 30 min	19	-	1.45	0.07	-	0.048
	MEF Tet1CD DMSO 30 min	14	-	1.11	0.02	-	-
	MEF mcherry DMSO 30 min	10	-	1.12	0.02	-	0.0873
S17A	Tet1CD	21	0.78	0.81	0.1	0.73 - 0.84	-
	mcherry	18	0.25	0.23	0.08	0.2 - 0.3	2.76E-11
S18C	MEF W8 30 min	26	-	2.38	0.13	-	0.0144
	MEF PM 30 min	34	-	2.09	0.12	-	-
	MEF W8 DMSO 30 min	31	-	1.17	0.02	-	0.433
	MEF PM DMSO 30 min	31	-	1.13	0.04	-	-
S20A	Nuclear H3K9ac J1 wt	29	-	52.38	8.29	-	-
	Nuclear H3K9ac J1 TKO	34	-	47.21	3.21	-	0.92
	Chromocentric H3K9ac J1 wt	29	-	41.42	3.3	-	-
	Chromocentric H3K9ac J1 TKO	34	-	47.9	8.57	-	0.47
	Nuclear H3K18ac J1 wt	31	-	52.62	9.79	-	-
	Nuclear H3K18ac J1 TKO	20	-	51.1	10.2	-	0.505
	Chromocentric H3K18ac J1 wt	31	-	47.8	2.4	-	-
	Chromocentric H3K18ac J1 TKO	20	-	51.8	4.35	-	0.384
	Nuclear H3K27ac J1 wt	47	-	37.03	5.71	-	-
	Nuclear H3K27ac J1 TKO	23	-	54.3	5.04	-	0.0967

Figure	Sample	n[a]	Median	Mean	StDev[a]	95% CI[a]	*p*-value[a]
	Chromocentric H3K27ac J1 wt	47	-	38.52	3.6	-	-
	Chromocentric H3K27ac J1 wt	23	-	49.02	6.8	-	0.073
	Nuclear H3K56ac J1 wt	33	-	83.58	9.18	-	-
	Nuclear H3K56ac J1 TKO	21	-	87.87	1.67	-	0.97
	Chromocentric H3K56ac J1 wt	33	-	85.73	13.39	-	-
	Chromocentric H3K56ac J1 TKO	21	-	83.01	14.25	-	0.835
	Nuclear H4K8ac J1 wt	28	-	48.66	12.14	-	-
	Nuclear H4K8ac J1 TKO	22	-	52.25	8.93	-	0.523
	Chromocentric H4K8ac J1 wt	28	-	51.2	9.04	-	-
	Chromocentric H4K8ac J1 TKO	22	-	57.8	6.27	-	0.368
	Nuclear H4K16ac J1 wt	26	-	49.97	8.14	-	-
	Nuclear H4K16ac J1 TKO	23	-	38.19	10.76	-	0.832
	Chromocentric H4K16ac J1 wt	26	-	63.8	8.34	-	-
	Chromocentric H4K16ac J1 TKO	23	-	57.8	4.63	-	0.551
S20B	Nuclear H3K9m3 J1 wt	30	-	49.08	11.01	-	-
	Nuclear H3K9m3 J1 TKO	26	-	38.82	7.19	-	0.16
	Chromocentric H3K9m3 J1 wt	30	-	80.46	8.82	-	-
	Chromocentric H3K9m3 J1 TKO	26	-	65.32	5.97	-	0.132
	Nuclear H4K20m3 J1 wt	34	-	19.83	4.83	-	-
	Nuclear H4K20m3 J1 TKO	29	-	21.65	7.33	-	0.622
	Chromocentric H4K20m3 J1 wt	34	-	38.09	7.95	-	-
	Chromocentric H4K20m3 J1 TKO	29	-	33.36	9.64	-	0.403

[a] n: number of cells of all replicates (if not stated otherwise), StDev: standard deviation, 95% CI: 95% confidence interval, p-value: calculated as stated in figure legends and material and methods section.
[b] n numbers refer to independent PCRs analyzed and p-value is calculated against the band intensities of the dC containing experiment.
[c] 2 way ANOVA statistical analysis did not reveal any significant difference (n.s.) between dC, d5mC and d5hmC.

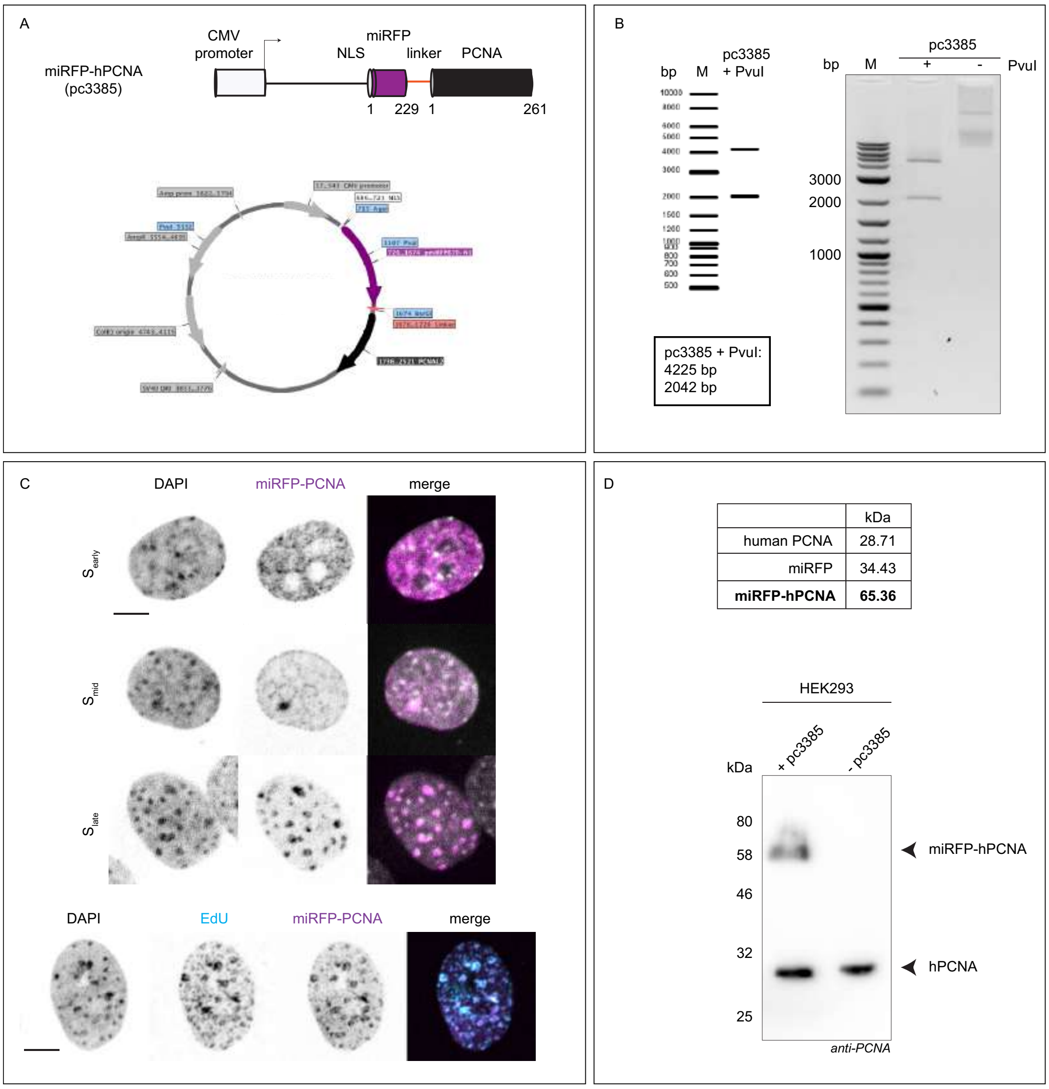

Supplementary Figure 1 – Characterization of the pmiRFP-PCNA plasmid. (A) miRFP was cloned into the RFP-hPCNA plasmid (pc1054) as described in the materials and methods section, to generate the miRFP-hPCNA plasmid (pc3385). The gene expression in mammalian cells is driven by a CMV promoter. Plasmid map shows all important vector components, including the restriction sites used for cloning (AgeI & BsrGI) and control digest (PvuI) of the recombinant plasmid. **(B)** Control digest of the miRFP-hPCNA construct using PvuI results in two bands of 4225 bp and 2042 bp, respectively. M stands for DNA size marker. **(C)** The subcellular localization of the miRFP-hPCNA construct was validated by microscopical analysis in transfected C2C12 mouse myoblast cells. The miRFP-hPCNA fusion protein showed the early, mid and late S-phase pattern described for somatic cells. S_{early} is characterized by a homogeneous distribution of replication throughout the nucleus, in S_{mid} replication signals are observed at the nucle(ol)ar periphery and S_{late} is marked by fewer but larger replication signals co-localizing with DAPI intense stained chromocenters. Labeling transfected cells with EdU resulted in a co-localization of the detection of nascent DNA (EdU) and the marked replisome component (miRFP-PCNA). **(D)** The expected miRFP-hPCNA fusion protein was detected via western blotting using a PCNA specific antibody. HEK293 cells were transfected with pc3385, lysed in loading dye and whole cell lysates were analyzed by SDS-PAGE gel electrophoresis followed by blotting onto a nitrocellulose membrane. Detection with anti-PCNA antibody showed bands at the size of the miRFP-hPCNA fusion protein (65 kDa) and the untagged endogenous PCNA (29 kDa), whereas the untransfected control cells (-pc3385) only showed signal for the endogenous PCNA. Scale bar = 5 µm. pc numbers refer to the plasmid collection numbers.

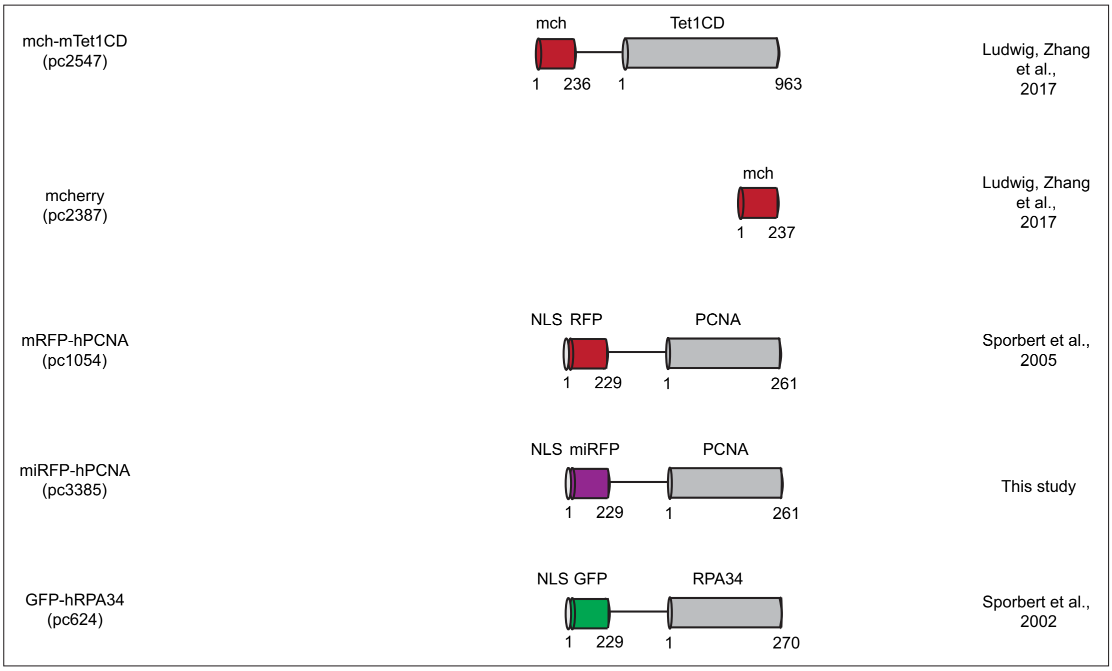

Supplementary Figure 2 – Schematic illustration of the relevant protein domains encoded in the plasmids used. Amino acid coordinates are indicated below every structure. pc numbers refer to the plasmid collection numbers. Respective publications are indicated. Plasmid construction details are described in the materials and methods part for the plasmids not published before. m: mouse, h: human, NLS: nuclear localization signal.

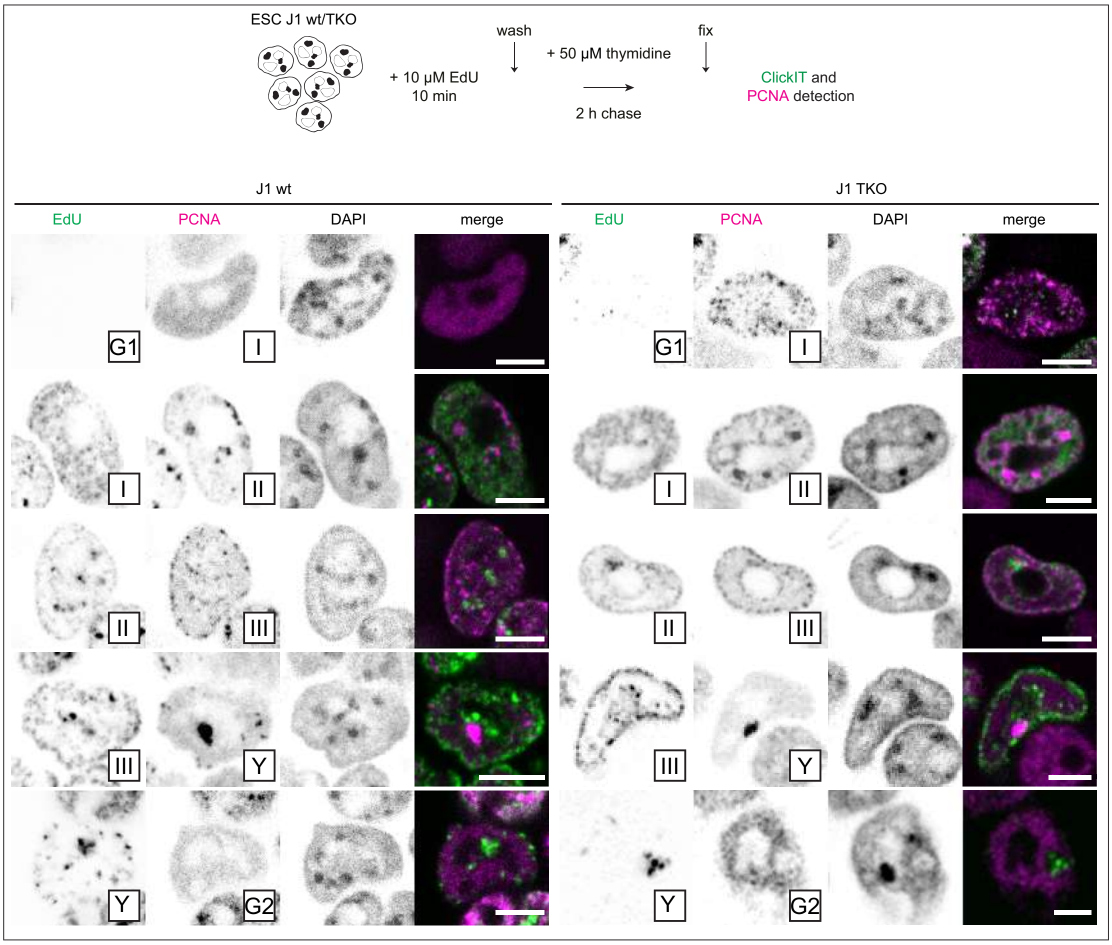

Supplementary Figure 3 – Effect of loss of global DNA methylation on the spatio-temporal progression of DNA replication in ES cells. J1 wt and TKO ES cells were pulse labeled with EdU and fixed after a chase period of 2 hours. (Immuno)fluorescent detection of the nucleotide analogue and endogenous PCNA allowed the analysis of the spatio-temporal progression of S-phase in fixed cells. Representative confocal images of the transitions from G1 to stage I, S-phase substage progressions and progression from stage Y to G2 are shown. Scale bar = 5 µm.

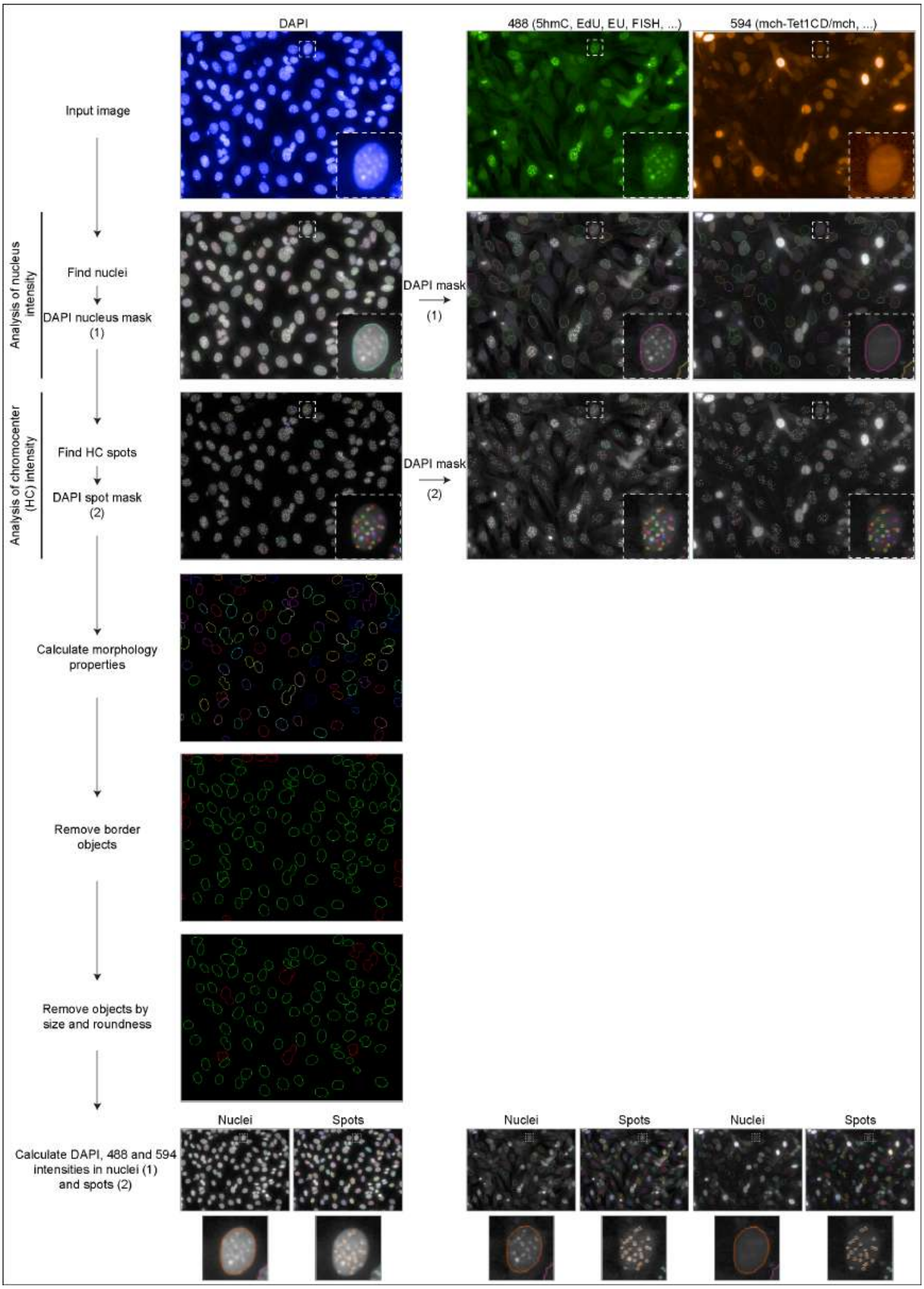

Supplementary Figure 4 – High content screening microscopy analysis pipeline. Analysis of high content screening microscopy images was done using the PerkinElmer Harmony software. Nuclei and chromocenters (HC spots) were found and masked based on the DAPI input images. DAPI masks were used to segment 488 nm and 594 nm channel images. Morphology properties of segmented nuclei were calculated (roundness, area, …), border objects were removed from the analysis and objects were further selected according to their size and roundness. Intensities of the DAPI, 488 and 594 channels were calculated for whole nuclei, chromocenter spots or nucleoli.

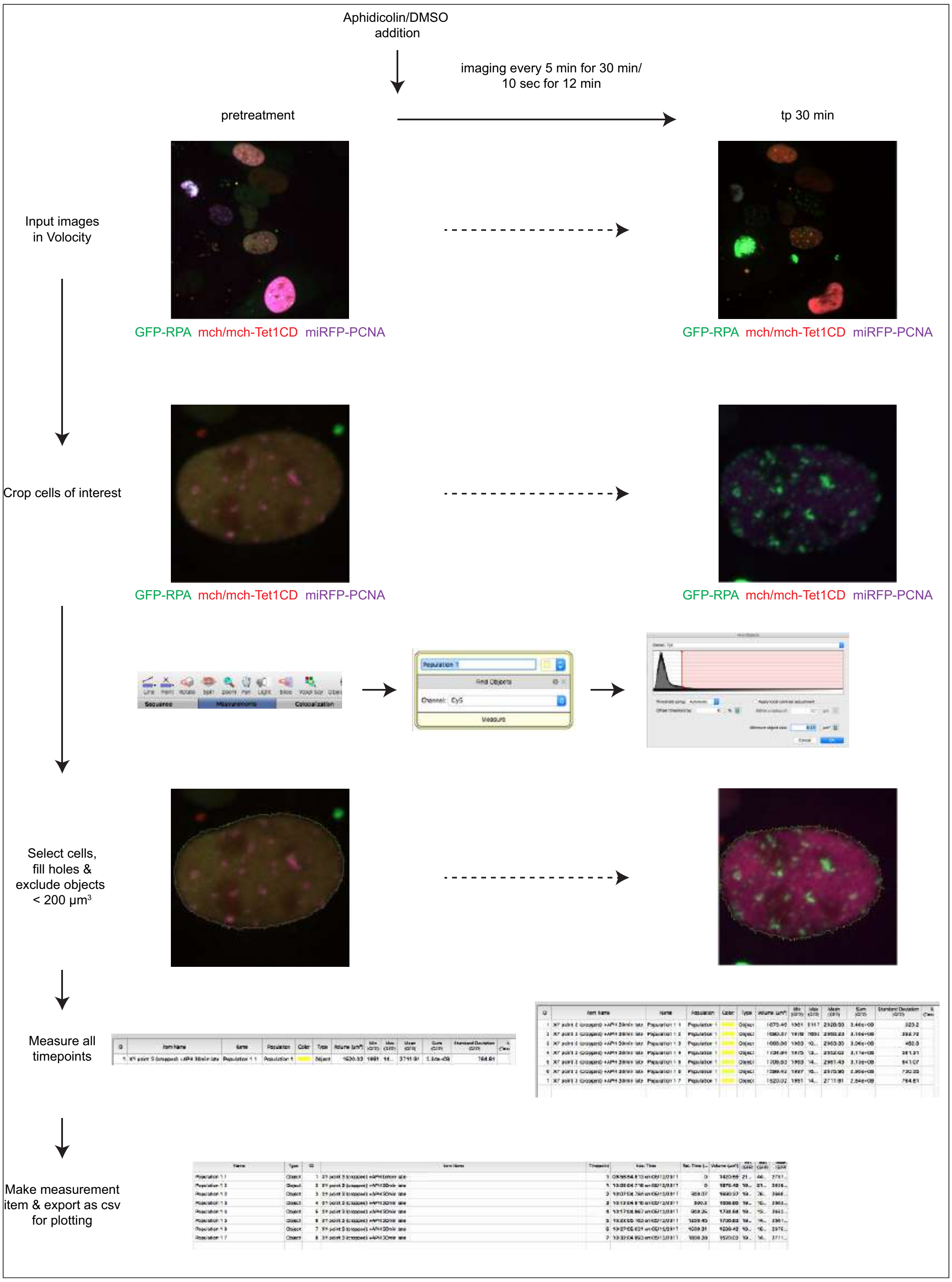

Supplementary Figure 5 – Live cell RPA accumulation analysis pipeline. Analysis of live cell microscopy images was done using the PerkinElmer Volocity software. Cells of interest were cropped in all images/timepoints. Cells were masked based on the GFP or miRFP signal. Objects smaller than 200 µm^3 were excluded and holes within the mask were filled. Signal intensities were measured in pretreatment and for all timepoints of treated cells. Measurements were grouped, saved and exported as csv files for further analysis and plotting as described in the Material and Methods section.

Figure 5A

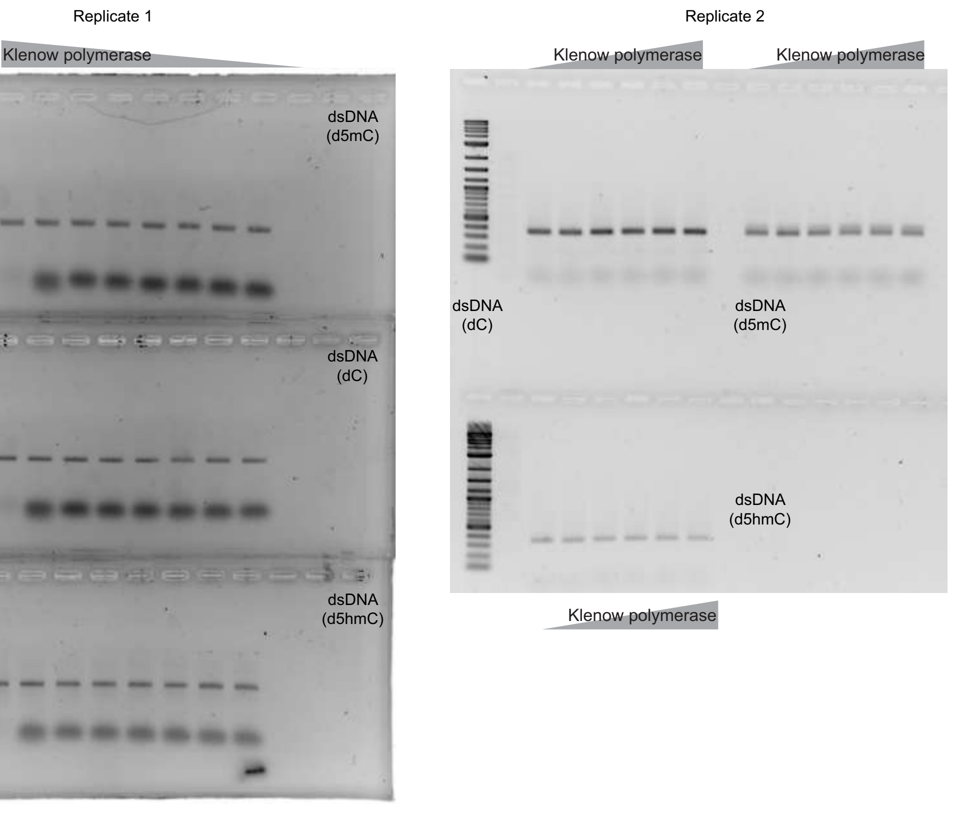

Figure 5D

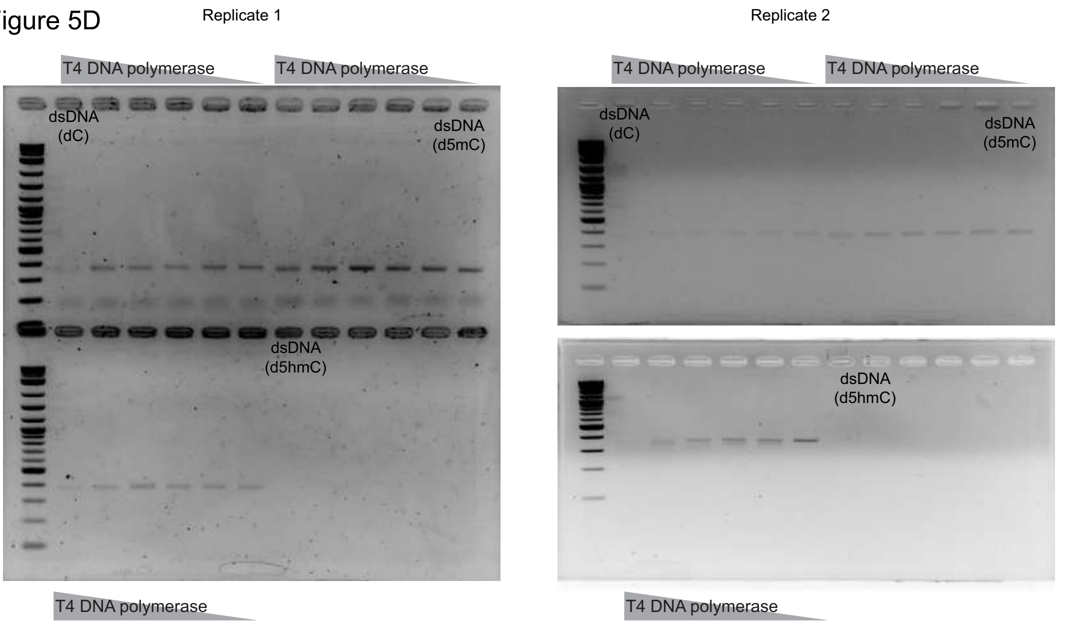

Supplementary Figure 6 – Full gel images used for the analysis of the Klenow (Figure 5A) and T4 polymerase assays (Figure 5D). The two independent replicates are shown.

Figure S8A

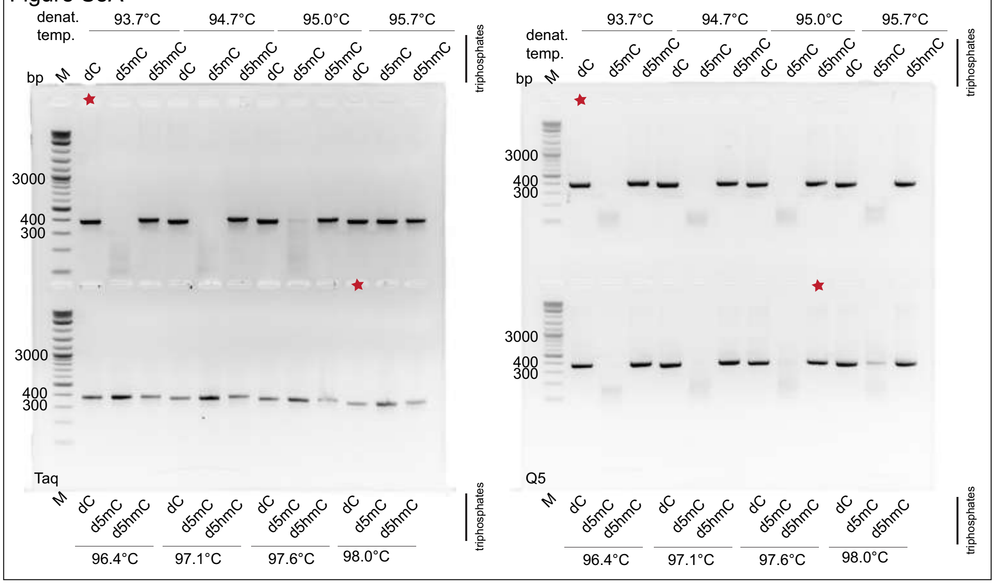

Figure S8C

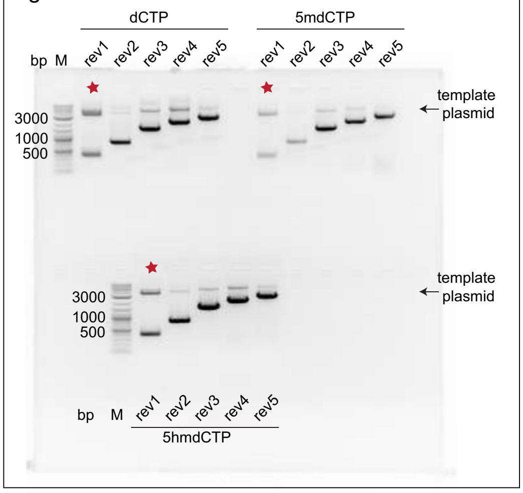

Figure S8D

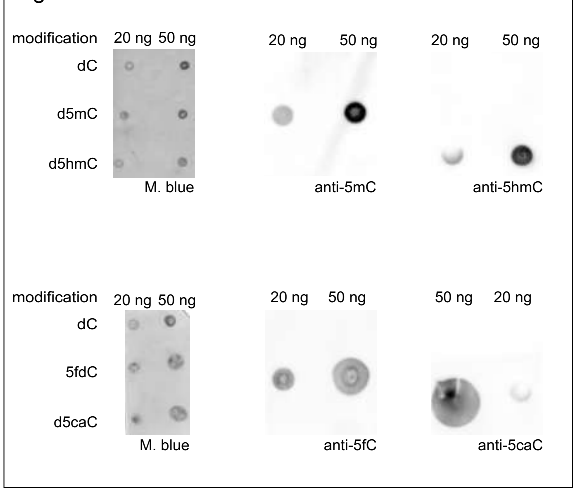

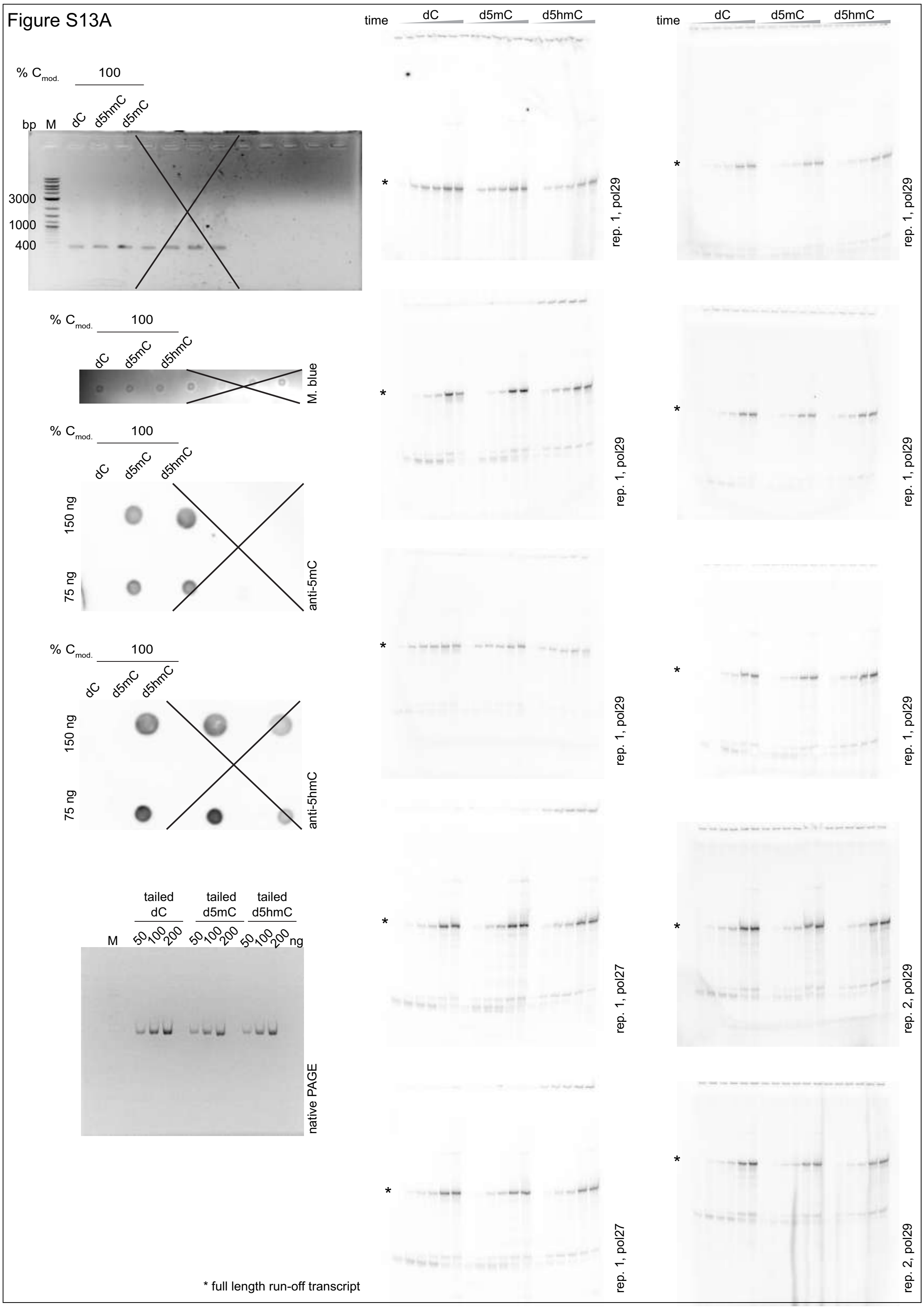
Figure S13A
% C mod.
100
bp
M
dC
d5hmC
d5mC
3000
1000
400
% C mod.
100
dC
d5mC
d5hmC
M. blue
% C mod.
100
dC
d5mC
d5hmC
150 ng
75 ng
anti-5mC
% C mod.
100
dC
d5mC
d5hmC
150 ng
75 ng
anti-5hmC
tailed dC
tailed d5mC
tailed d5hmC
M
50 100 200
50 100 200
50 100 200 ng
native PAGE
* full length run-off transcript
time
dC
d5mC
d5hmC
rep. 1, pol29
rep. 1, pol29
rep. 1, pol29
rep. 1, pol27
rep. 1, pol27
time
dC
d5mC
d5hmC
rep. 1, pol29
rep. 1, pol29
rep. 1, pol29
rep. 2, pol29
rep. 2, pol29

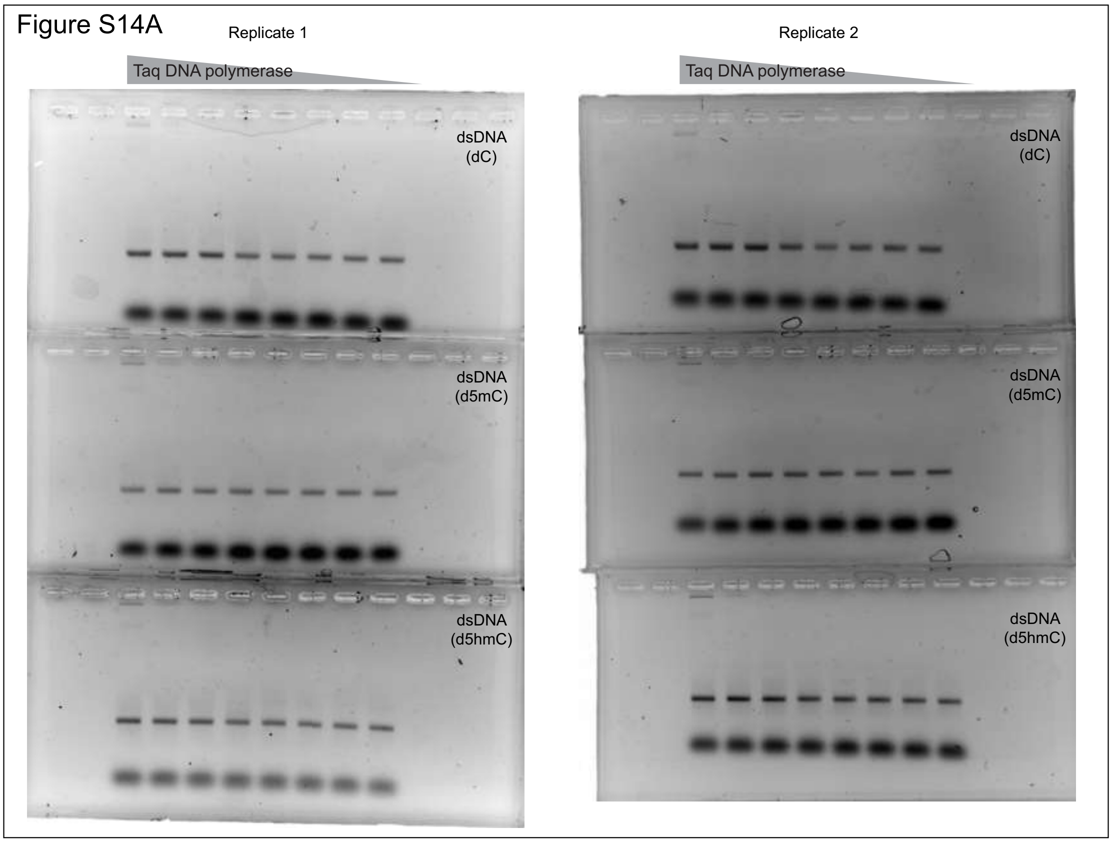
Figure S14A
Replicate 1
Replicate 2
Taq DNA polymerase
Taq DNA polymerase
dsDNA (dC)
dsDNA (d5mC)
dsDNA (d5hmC)
dsDNA (dC)
dsDNA (d5mC)
dsDNA (d5hmC)

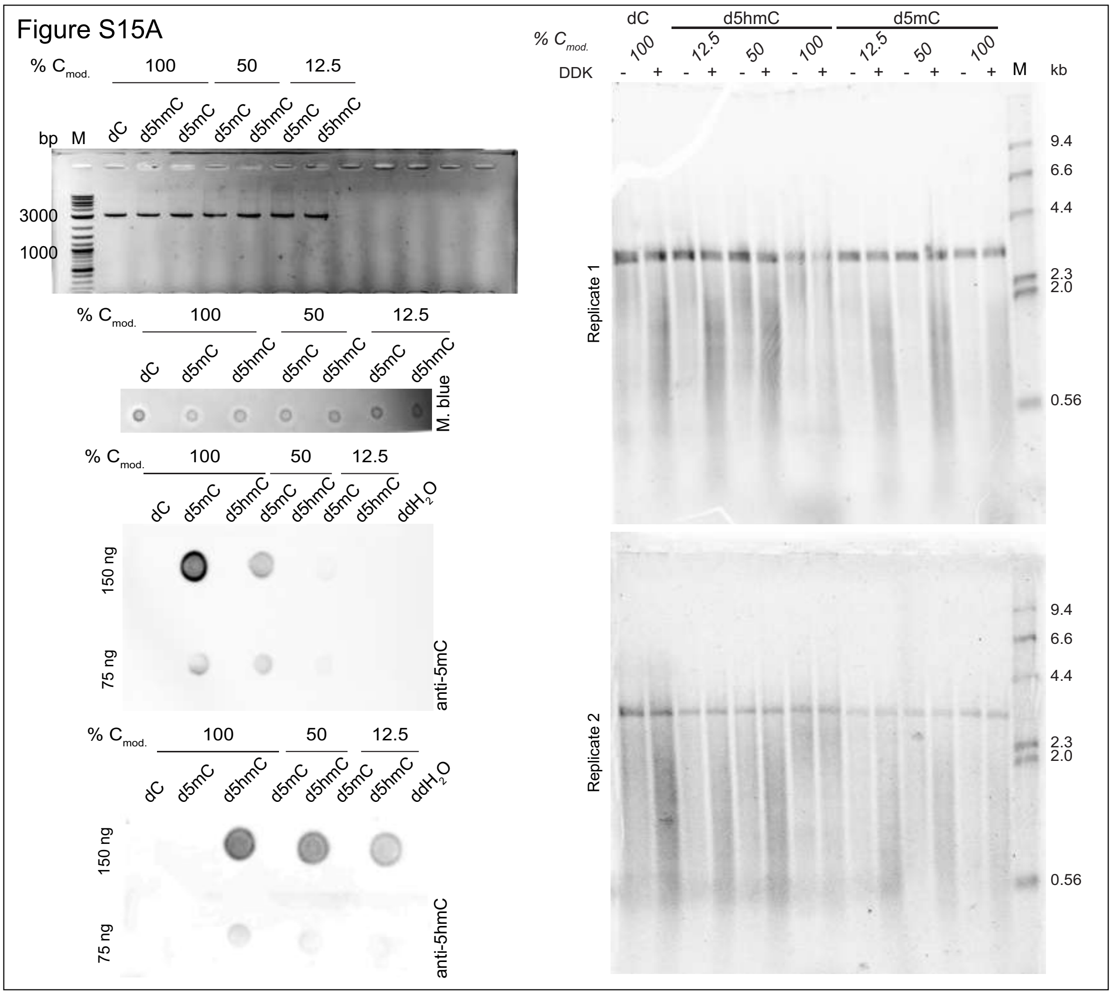
Figure S15A
% C mod.
100
50
12.5
bp M dC d5hmC d5mC d5mC d5hmC d5mC d5hmC
3000
1000
% C mod.
100
50
12.5
dC d5mC d5hmC d5mC d5hmC d5mC d5hmC
M. blue
% C mod.
100
50
12.5
dC d5mC d5hmC d5mC d5hmC d5mC d5hmC ddH2O
150 ng
75 ng
anti-5mC
% C mod.
100
50
12.5
dC d5mC d5hmC d5mC d5hmC d5mC d5hmC ddH2O
150 ng
75 ng
anti-5hmC
dC
d5hmC
d5mC
% C mod.
100 12.5 50 100 12.5 50 100
DDK - + - + - + - + - + - + - +
M
kb
9.4
6.6
4.4
2.3
2.0
0.56
Replicate 1
Replicate 2

Supplementary Figure 7 – Full gel images used for polymerase efficiency assays (Supplementary Figure 8A), PCR yield assays (Supplementary Figure 8C), slot blot analysis of modified PCR fragments (Supplementary Figure 8D), *in vitro* yeast RNA polymerization assays (Supplementary Figure 13A), Taq polymerase assays (Supplementary Figure 14A) and *in vitro* yeast replication assays (Supplementary Figure 15A). The independent replicates of the assays are shown if applicable. Crossed out parts of gels and blots were not used in the manuscript.

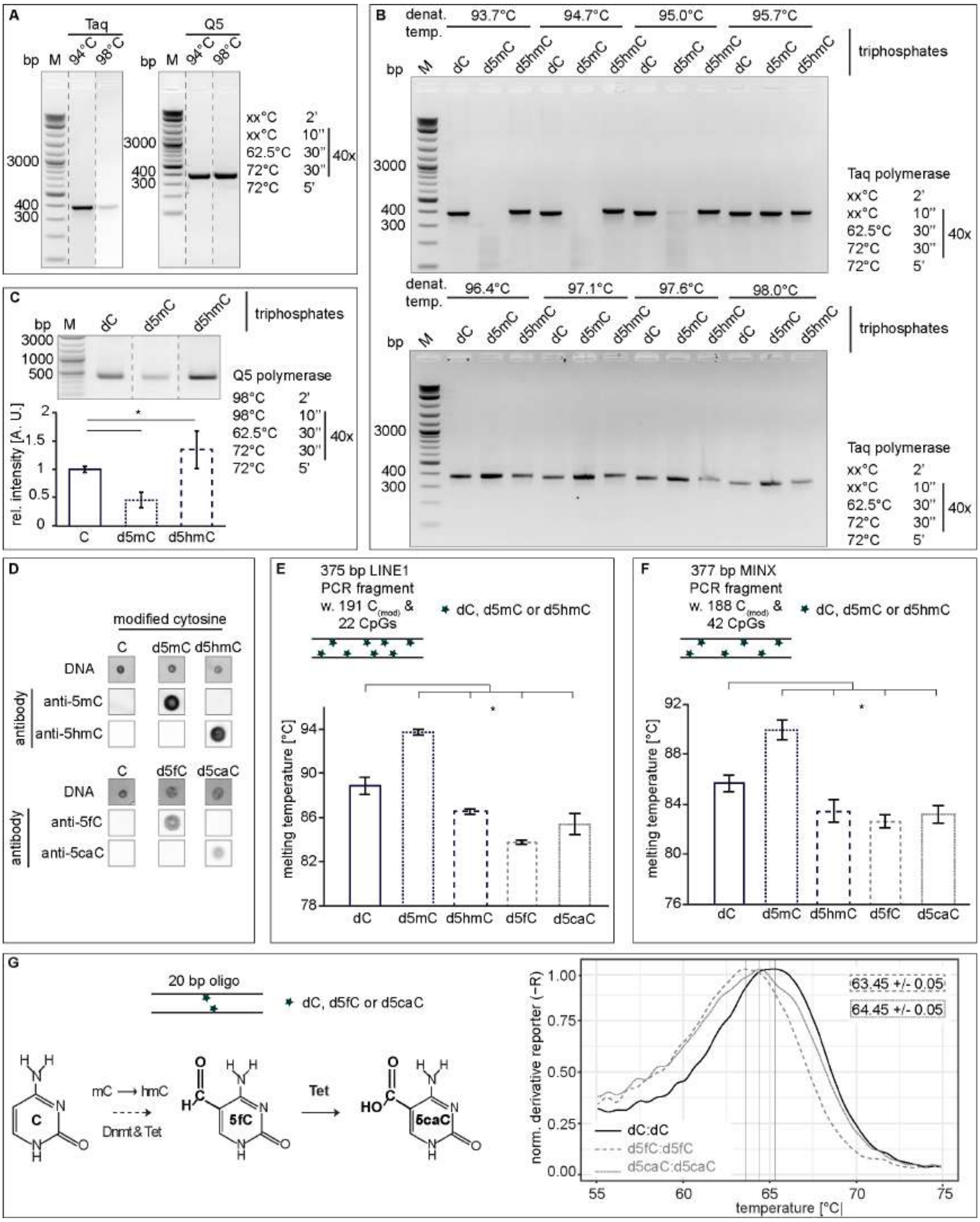

Supplementary Figure 8 – Effect of cytosine modifications on DNA double helix stability *in vitro*. (A) Agarose gel electrophoretic analysis of MINX PCR amplification efficiency obtained with Taq and Q5 polymerase with dCTP and using denaturing temperatures of 94°C and 98°C. **(B)** Agarose gel electrophoretic analysis of MINX PCR amplification products generated with Taq polymerase, denaturing temperatures between 93°C and 98°C and using dCTP, d5mCTP or d5hmCTP. **(C)** Agarose gel electrophoretic analysis of MINX PCR amplification product yields obtained with Q5 polymerase and using dCTP, d5mCTP or d5hmCTP and normalized quantification of PCR band intensities. **(D)** Slot blot analysis of dC, d5mC, d5hmC, d5fC and d5caC PCR amplicons. 20 ng of DNA were loaded and membranes were stained with methylene blue as a loading control (DNA). Membranes were incubated with anti-5mC, anti-5hmC, anti-5fC and anti-5caC antibodies, respectively and detected with an HRP conjugated secondary antibody. Exposure times and image contrast were adjusted for every antibody. **(E-F)**, Melting temperature of LINE1 PCR

amplicons (**E**) and MINX PCR amplicons (**F**) containing 100% dC, d5mC, d5hmC, d5fC or d5caC, respectively (except primer sites), as determined by high resolution melting (HRM) temperature analysis. **(G)** Modified cytosine bases with the respective writer enzymes are depicted. High resolution melting temperature (HRM) analysis of 20 bp oligonucleotides containing one central (modified) CpG dinucleotide (CpG, 5fCpG or 5caCpG). Independent experiments were repeated in triplicates with four technical replicates (**E, F** and **G**), error bars represent standard deviation and *p* and n-values are summarized in Supplementary Table 8. Complete PCR conditions are indicated. M stands for DNA size markers. All quantifications of band intensities are depicted as a ratio to the respective controls. Full gels and blots are shown in Supplementary Figure 7. * $p < 0.05$.

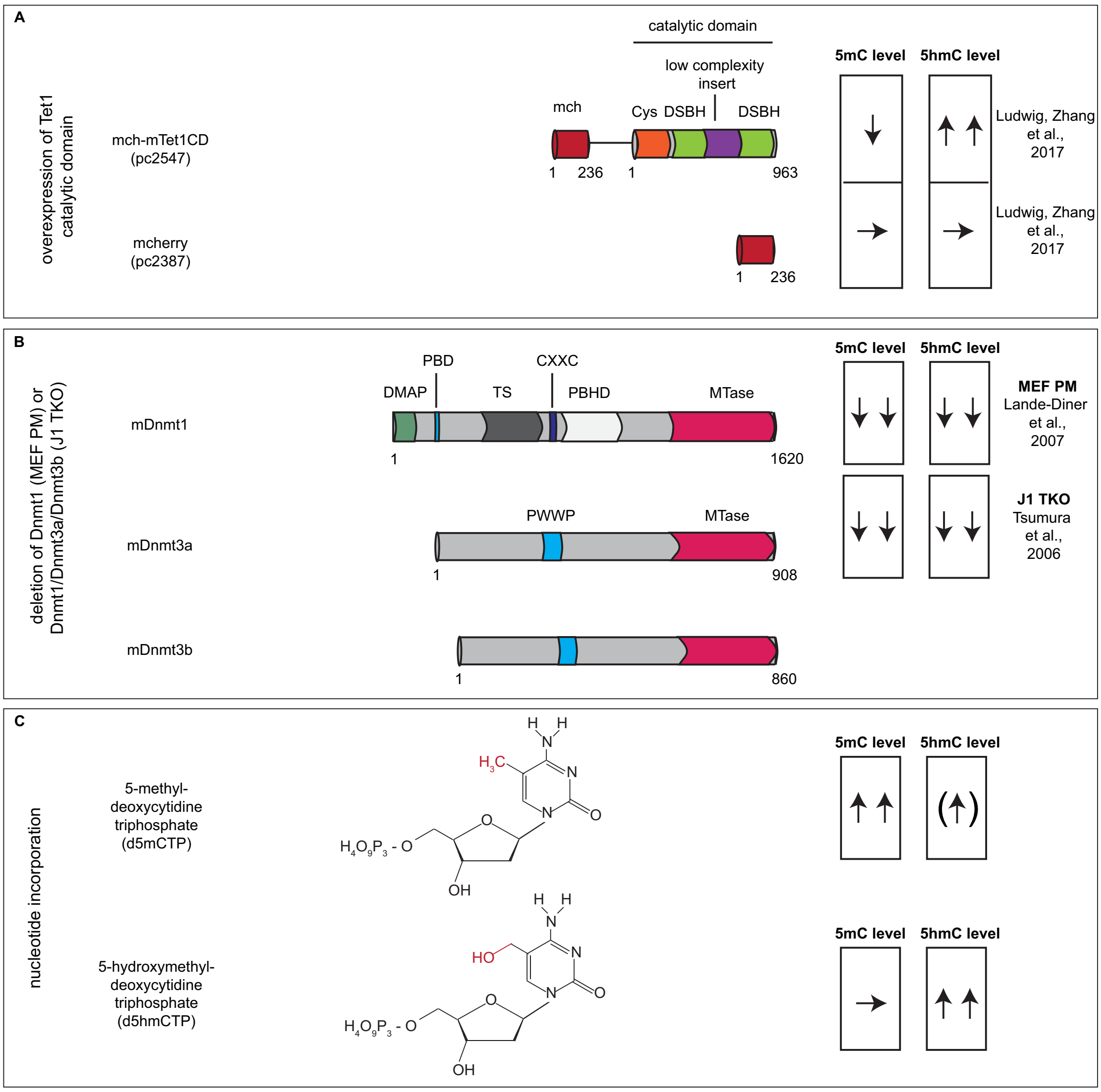

Supplementary Figure S9 – Graphical summary of the strategies and outcomes for manipulation of cytosine levels in cells. Amino acid coordinates are indicated below every structure. pc numbers refer to the plasmid collection numbers. **(A)** Overexpressing constructs with mcherry tagged Tet1 catalytic domain lead to increased 5hmC levels. **(B)** Genomic knock-outs of Dnmt1 (MEF PM) or Dnmt1, Dnmt3a and Dnmt3b (J1 TKO) lead to the loss of all DNA cytosine modifications. **(C)** Transfection of cells with methylated or hydroxymethylated cytosine nucleotides lead to increased genomic 5mC and 5hmC levels, respectively.

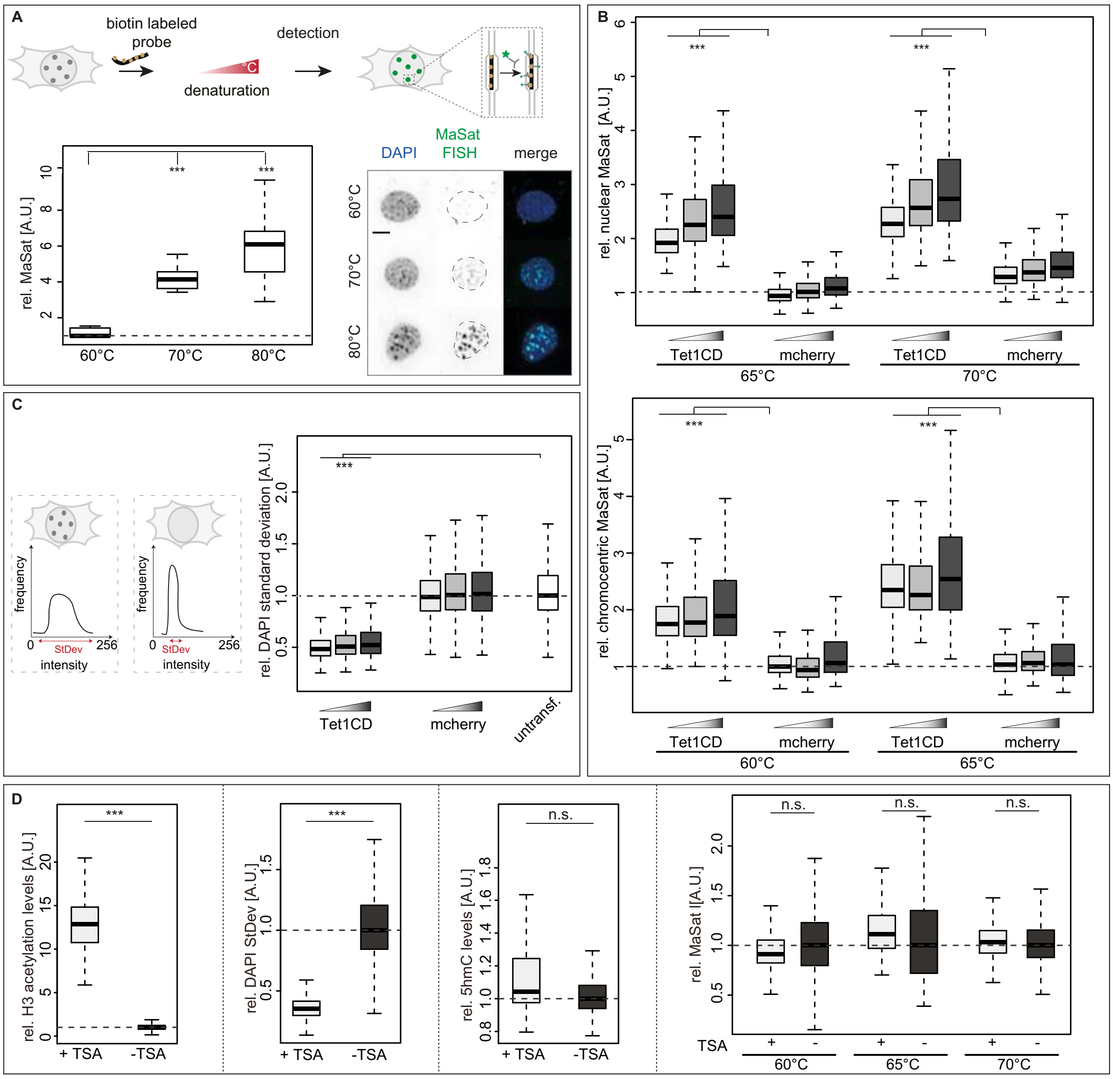

Supplementary Figure 10 – Effect of cytosine modifications on genomic DNA double helix stability *in vivo*. **(A)** Experimental setup to determine the optimal denaturing temperatures for major satellite DNA FISH assays. Relative major satellite DNA FISH sum intensities of C2C12 cells denatured at 60°C, 70°C or 80°C and representative spinning disk confocal images for every denaturing temperature are shown. **(B)** Relative major satellite DNA FISH sum intensities on transfected C2C12 mouse myoblast cells are plotted. DNA denaturation was done at 60°C, 65°C and 70°C, respectively. MaSat signal intensities were measured in the whole nucleus (defined by DAPI counterstaining, up) and in pericentric heterochromatin (chromocenters, down). **(C)** On a histogram, condensed chromatin is characterized by high DAPI intensities. Thus, cell nuclei containing condensed chromatin show a higher DAPI standard deviation than cells containing decondensed chromatin, which is characterized by low DAPI standard deviations. Normalized DAPI standard deviation values, as a proxy for DNA decondensation levels, 24 hours post transfection of C2C12 cells are plotted. **(D)** Relative histone H3 acetylation sum intensities, DNA decondensation, 5hmC and major satellite DNA FISH sum intensities in C2C12 cells after 72 hours of TSA treatment. Independent experiments were done in duplicates (**A**) or in triplicates (**B, C** and **D**). All fluorescent signals are plotted as a ratio to the values of the respective control cells. All boxes and whiskers represent 25–75 percentiles and 1.5 times the IQD (interquartile distance), respectively and the center line depicts the median. *p* and n-values are summarized in Supplementary Table 8. Scale bar = 5 µm. *** $p < 0.001$, n.s.: non-significant and CC: chromocenters.

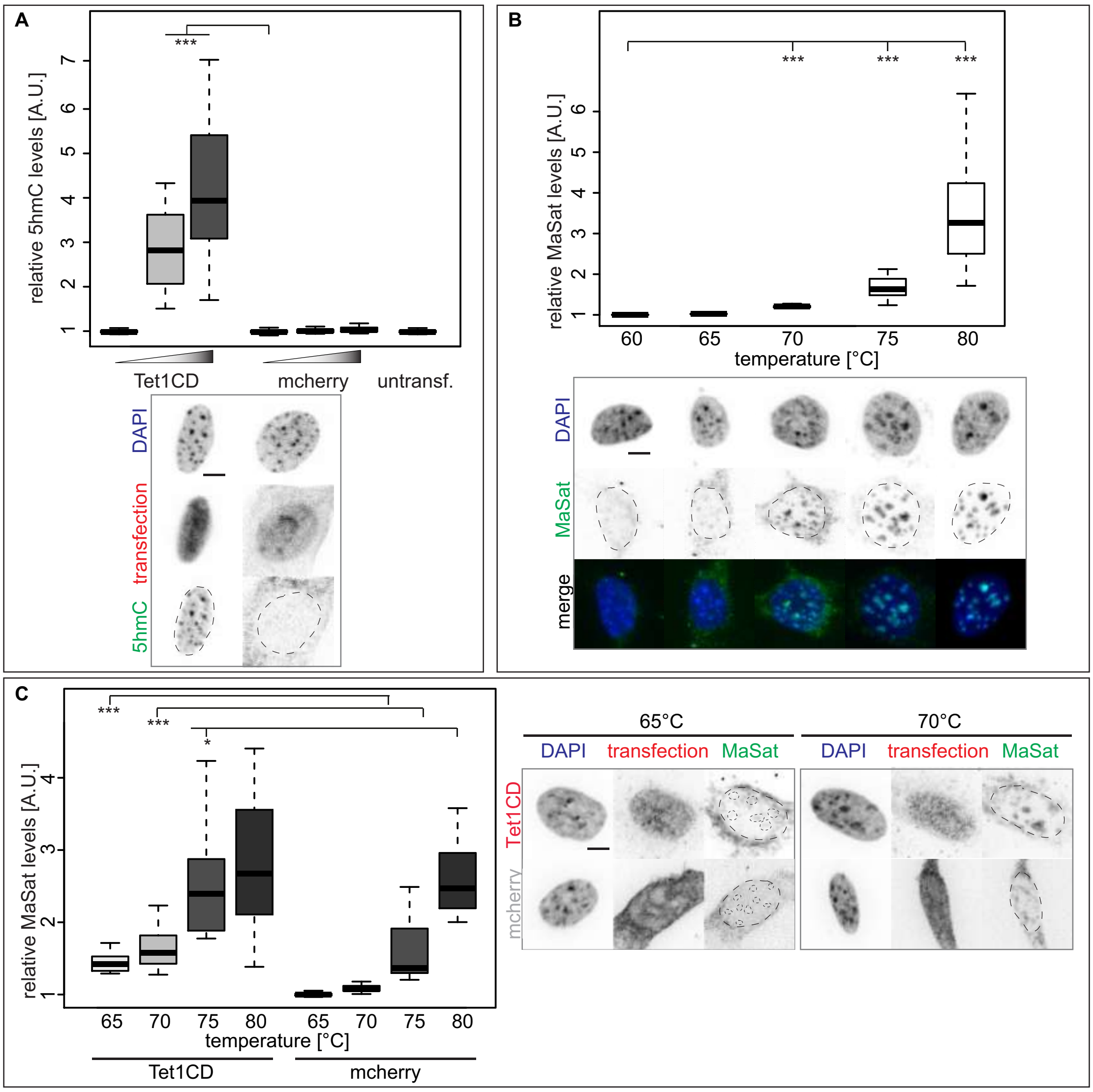

Supplementary Figure 11 – Effect of cytosine modifications on DNA double helix stability in mouse embryonic fibroblasts *in vitro*. (A) Relative 5hmC sum intensities in MEF cells, 24 hours after transfection and representative spinning disk confocal images for every transfection condition are shown. **(B)** Relative major satellite DNA FISH sum intensity signals of MEF cells with denaturing temperatures of 60°C, 65°C, 70°C, 75°C and 80°C and representative spinning disk confocal images for every denaturing temperature are depicted. **(C)** Relative nuclear major satellite DNA FISH sum intensities transfected mouse embryonic fibroblast cells with DNA denaturation at 65°C, 70°C, 75°C and 80°C, respectively are plotted. Representative spinning disk confocal images at 65°C and 70°C are shown. Independent experiments were done in triplicates. Dotted lines represent nuclear and chromocenter contours. All boxes and whiskers are as in Supplementary Figure 10 and *p* and n-values are summarized in Supplementary Table 8. Scale bar = 5 µm. * $p < 0.05$ and *** $p < 0.001$.

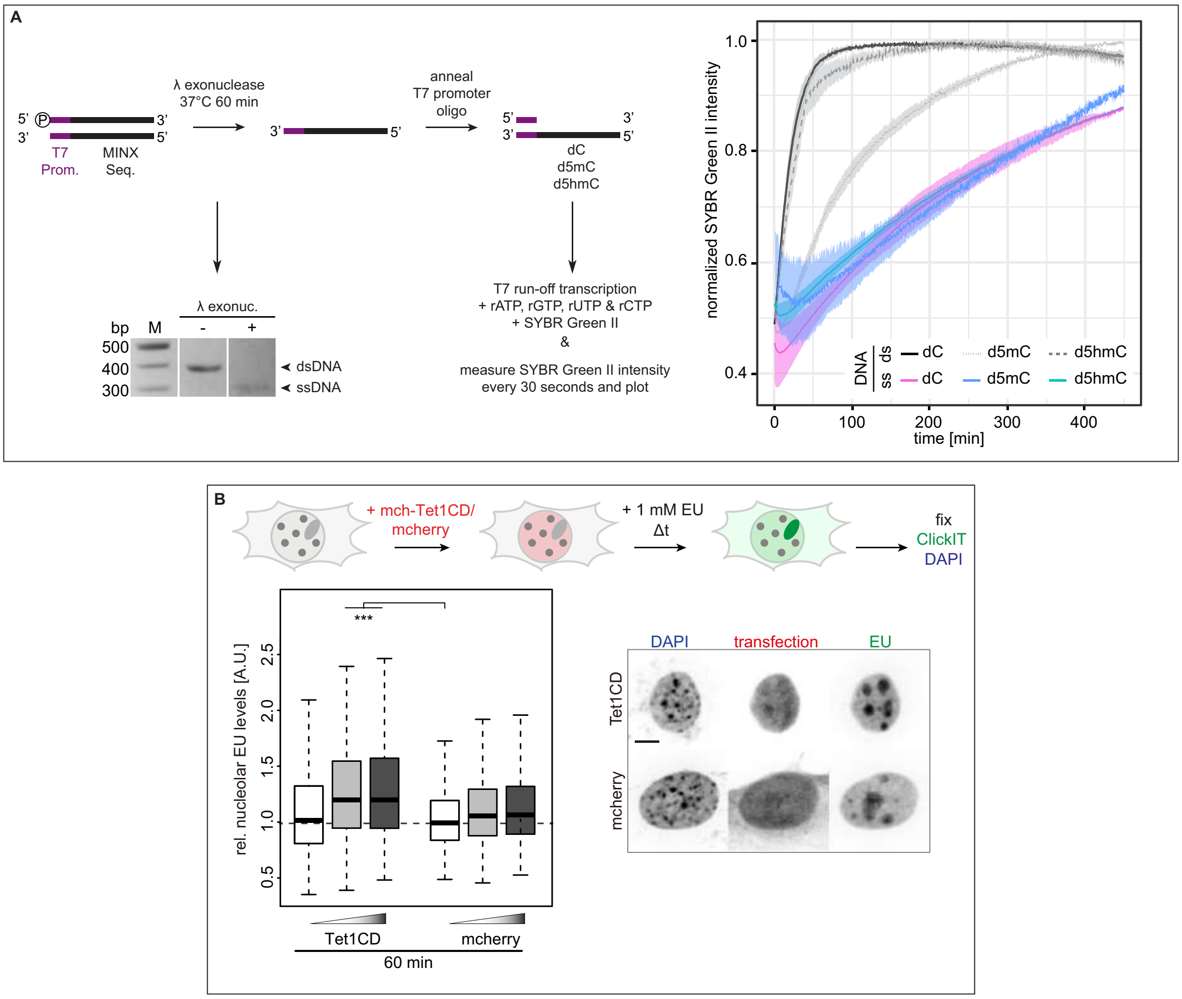

Supplementary Figure 12 – Effect of cytosine modifications on RNA polymerase activity *in vivo*. (A) Experimental setup for *in vitro* T7 run-off transcription on double-stranded (dsDNA) and single-stranded (ssDNA). To generate ssDNA templates, dsDNA PCR amplicons with a 5' phosphorylated forward strand were digested with λ exonuclease. To allow polymerase binding, ssDNA templates contained a dsDNA T7 promoter region. Template DNA contained 100% cytosine (dC), methylcytosine (d5mC) or hydroxymethylcytosine (d5hmC) and T7 promoter sequence was unmodified. Normalized mean SYBR green II intensities (± standard deviation), i.e., the amount of RNA transcripts, over time are shown. **(B)** Schematic representation of the experimental setup for *in vivo* ribonucleotide incorporation analysis. Transfected cells were incubated with 1 mM EU 24 hours post transfection, EU was detected and nucleolar EU sum intensities are plotted. Representative spinning disk confocal images of every transfection condition are depicted. Independent experiments were done in triplicates. All boxes and whiskers are as in Supplementary Figure 10 and p and n-values are summarized in Supplementary Table 8. Scale bar = 5 µm. *** $p < 0.001$.

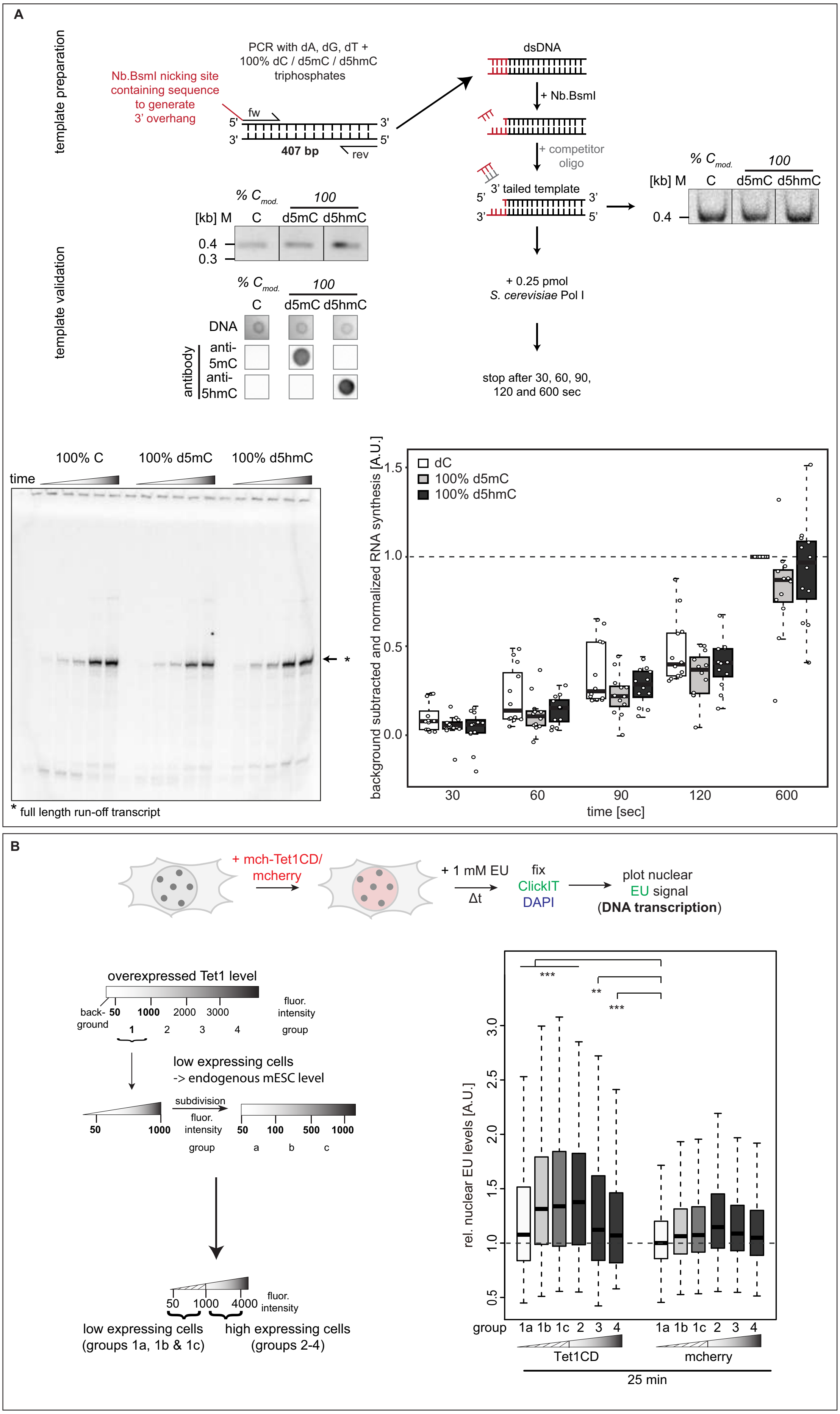
A
template preparation
Nb.BsmI nicking site
containing sequence
to generate
3' overhang
PCR with dA, dG, dT +
100% dC / d5mC / d5hmC
triphosphates
fw
5'
3'
3'
5'
407 bp
rev
dsDNA
+ Nb.BsmI
+ competitor
oligo
3' tailed template
5'
3'
3'
5'
% C mod.
100
[kb] M
C
d5mC d5hmC
0.4
template validation
% C mod.
100
[kb] M
C
d5mC d5hmC
0.4
0.3
% C mod.
100
C
d5mC d5hmC
DNA
antibody
anti-
5mC
anti-
5hmC
+ 0.25 pmol
S. cerevisiae Pol I
stop after 30, 60, 90,
120 and 600 sec
100% C
100% d5mC
100% d5hmC
time
*
* full length run-off transcript
background subtracted and normalized RNA synthesis [A.U.]
dC
100% d5mC
100% d5hmC
1.5
1.0
0.5
0.0
30
60
90
120
600
time [sec]
B
+ mch-Tet1CD/
mcherry
+ 1 mM EU
Δt
fix
ClickIT
DAPI
plot nuclear
EU signal
(DNA transcription)
overexpressed Tet1 level
back-
ground
50
1000
2000
3000
fluor.
intensity
1
2
3
4
group
low expressing cells
-> endogenous mESC level
subdivision
50
1000
fluor.
intensity
group
50
100
500
1000
a
b
c
50
1000
4000
fluor.
intensity
low expressing cells
(groups 1a, 1b & 1c)
high expressing cells
(groups 2-4)
rel. nuclear EU levels [A.U.]
***
**
***
3.0
2.5
2.0
1.5
1.0
0.5
group
1a 1b 1c 2 3 4
1a 1b 1c 2 3 4
Tet1CD
mcherry
25 min

Supplementary Figure 13 – Effect of cytosine modifications on eukaryotic RNA polymerase activity. (A) Experimental setup and biochemical validation of the template generation for *in vitro* polymerization assays. Template DNA contained 100% cytosine (dC) or methylcytosine (d5mC) or hydroxymethylcytosine (d5hmC), respectively (except primer sites). Exemplary agarose gel images and slot blot analysis of dC, d5mC and d5hmC levels are shown. Experimental setup for RNA polymerization assay using purified *S. cerevisiae* Polymerase I (Pol I). DNA templates were digested with the nicking enzyme Nb.BsmI to generate templates with a 3' overhang. Equal DNA amounts after nicking and DNA cleanups are confirmed by native PAGE gel analysis. An exemplary urea-PAGE gel image is shown. Background subtracted and normalized (to dC) full length run-off transcript intensities (400 bp, marked by *) as a proxy for RNA polymerization are shown. **(B)** Schematic representation of the experimental setup for *in vivo* ribonucleotide incorporation analysis and representation of the cell grouping and binning approach applied. Cells were divided into four main groups, depending on their Tet1CD/mcherry expression levels (group 1: low expressing cells and groups 2-4: high expressing cells). Cells with Tet1 expression levels corresponding to group 1 (fluorescence intensity 50-1000, low Tet1 expressing cells) were further subdivided in three subgroups (fluorescent intensities group 1a: 50-100, group 1b: 100-500 and group 1c: 500-1000) during plotting. Cells were incubated with 1 mM EU 24 hours after transfection, EU was detected and nuclear EU sum intensities are plotted. Data from groups 1a-1a are the same as plotted in Figure 4B. Independent experiments were done with two DNA replicates, two different polymerase batches and in 12 replicates (A) or in triplicates (B) and all boxes and whiskers are as in Supplementary Figure 10. *p* and n-values are summarized in Supplementary Table 8. Full gels are shown in Supplementary Figure 7. * $p < 0.05$, ** $p < 0.005$ and *** $p < 0.001$.

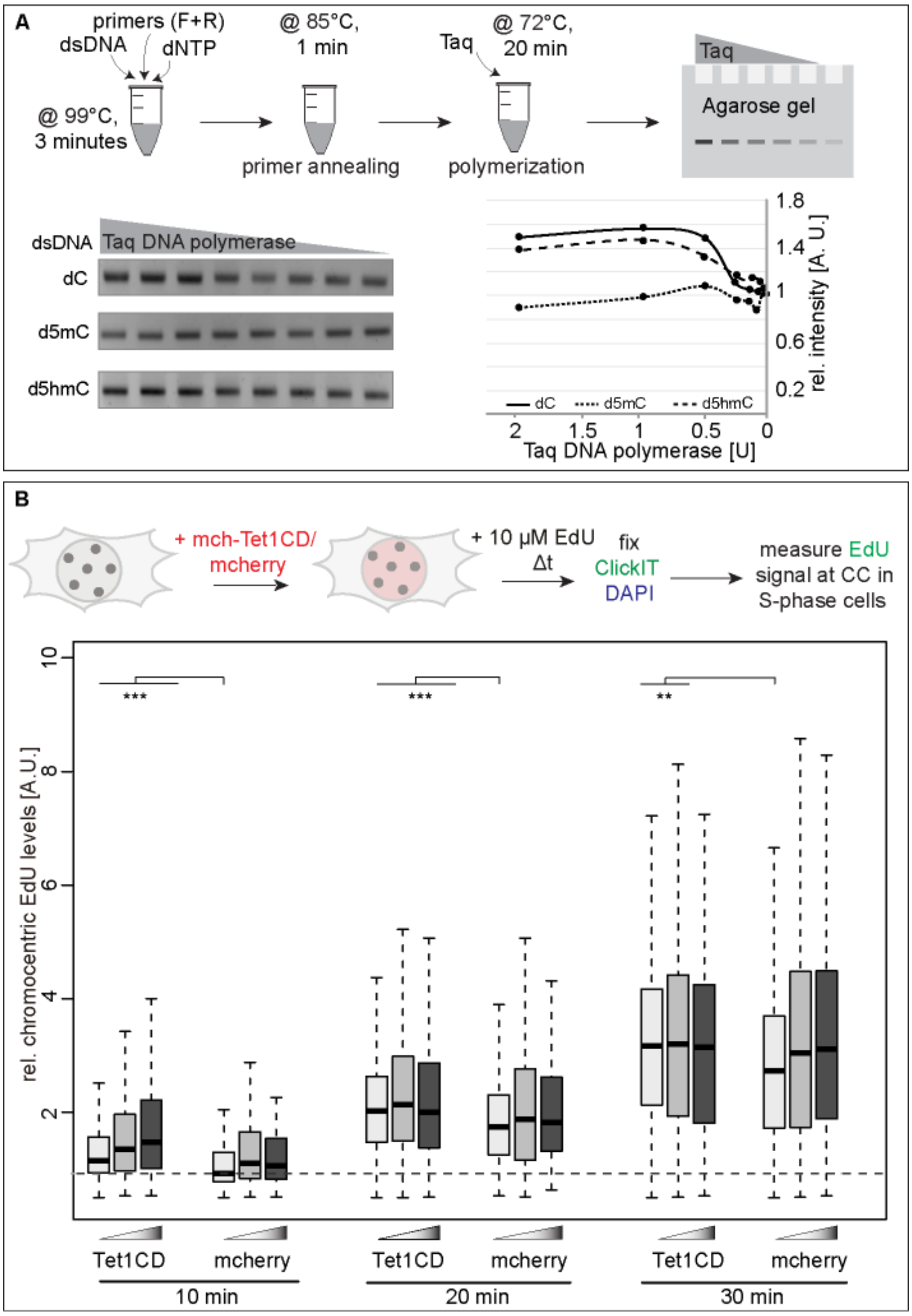

Supplementary Figure 14 – Effect of cytosine modifications on DNA polymerase activity. (A) Experimental setup for *in vitro* Taq polymerization assay. Template DNA contained 100% cytosine (dC), methylcytosine (d5mC) or hydroxymethylcytosine (d5hmC) (except primer sites). Exemplary gel images for Taq polymerase assay and sum band intensity quantification as a proxy for DNA polymerization, normalized to the DNA input (0 U enzyme) are shown. **(B)** Schematic representation of the experimental setup for *in vivo* nucleotide incorporation analysis. Cells were incubated with 10 µM EdU 24 hours after transfection, EdU was detected and nuclear EdU sum intensities of S-phase cells are plotted. Independent experiments were done twice (**A**) or in triplicates (**B**) and all boxes and whiskers are as in Supplementary Figure 10. *p* and n-values are summarized in Supplementary Table 8. Full gels are shown in Supplementary Figure 7. *** $p < 0.001$.

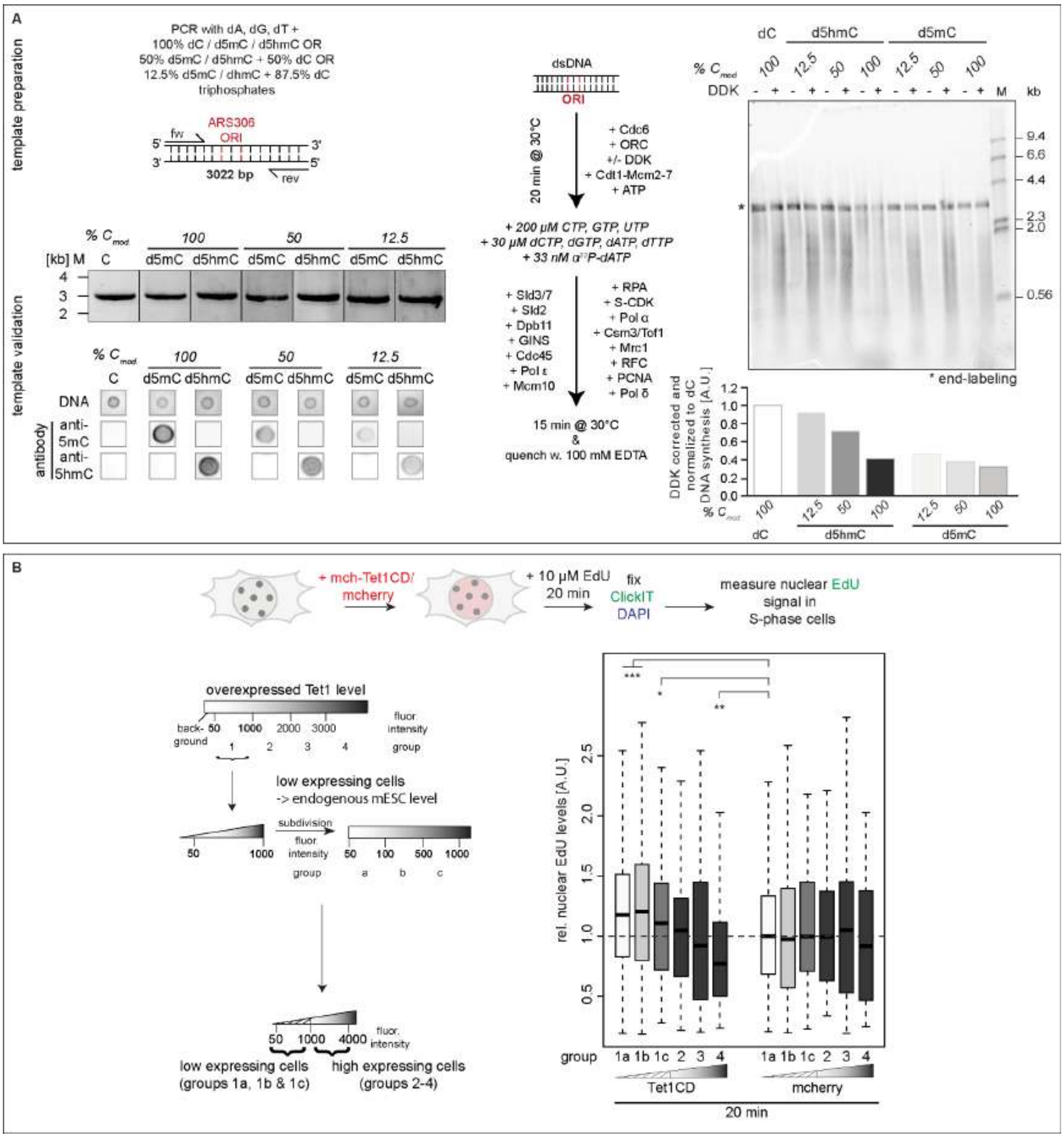

Supplementary Figure 15 – Effect of various amounts of cytosine modifications on eukaryotic DNA polymerase activity. (A) Experimental setup and biochemical validation of the template generation for *in vitro* polymerization assays. Template DNA contained 100% cytosine (dC) or 100%, 50% or 12.5% methylcytosine (d5mC) or hydroxymethylcytosine (d5hmC), respectively (except primer sites). The 3 kb templates also contained a centrally arranged yeast ARS306 origin of replication. Exemplary agarose gel images and slot blot analysis of dC, d5mC and d5hmC levels for every nucleotide composition are shown. Experimental setup for soluble replication assay using purified *S. cerevisiae* replisome components. Exemplary alkaline agarose gel images are shown and DDK subtracted and normalized replicated DNA signal intensities (< 3 kb end-labeling band (labeled with *), normalized to dC) as a proxy for DNA polymerization are shown. **(B)** Schematic representation of the experimental setup for *in vivo* nucleotide incorporation analysis and representation of the cell grouping and binning approach applied. Cells were divided into four main groups, depending on their Tet1CD/mcherry expression levels (group 1: low expressing cells and groups 2-4: high expressing cells). Cells with Tet1 expression levels corresponding to group 1 (fluorescence intensity 50-1000, low Tet1 expressing cells) were further subdivided in three subgroups (fluorescent intensities group 1a: 50-100, group 1b: 100-500 and group 1c: 500-1000) during plotting. Cells were incubated with 10 µM EdU 24 hours after transfection, EdU was detected and nuclear EdU sum intensities of S-phase cells are plotted.

Data from groups 1a-1a are the same as plotted in Figure 5B. Independent experiments were done twice (A) or in triplicates (B) and all boxes and whiskers are as in Supplementary Figure 10. *p* and n-values are summarized in Supplementary Table 8. Full gels are shown in Supplementary Figure 7. * $p < 0.05$, ** $p < 0.005$ and *** $p < 0.001$.

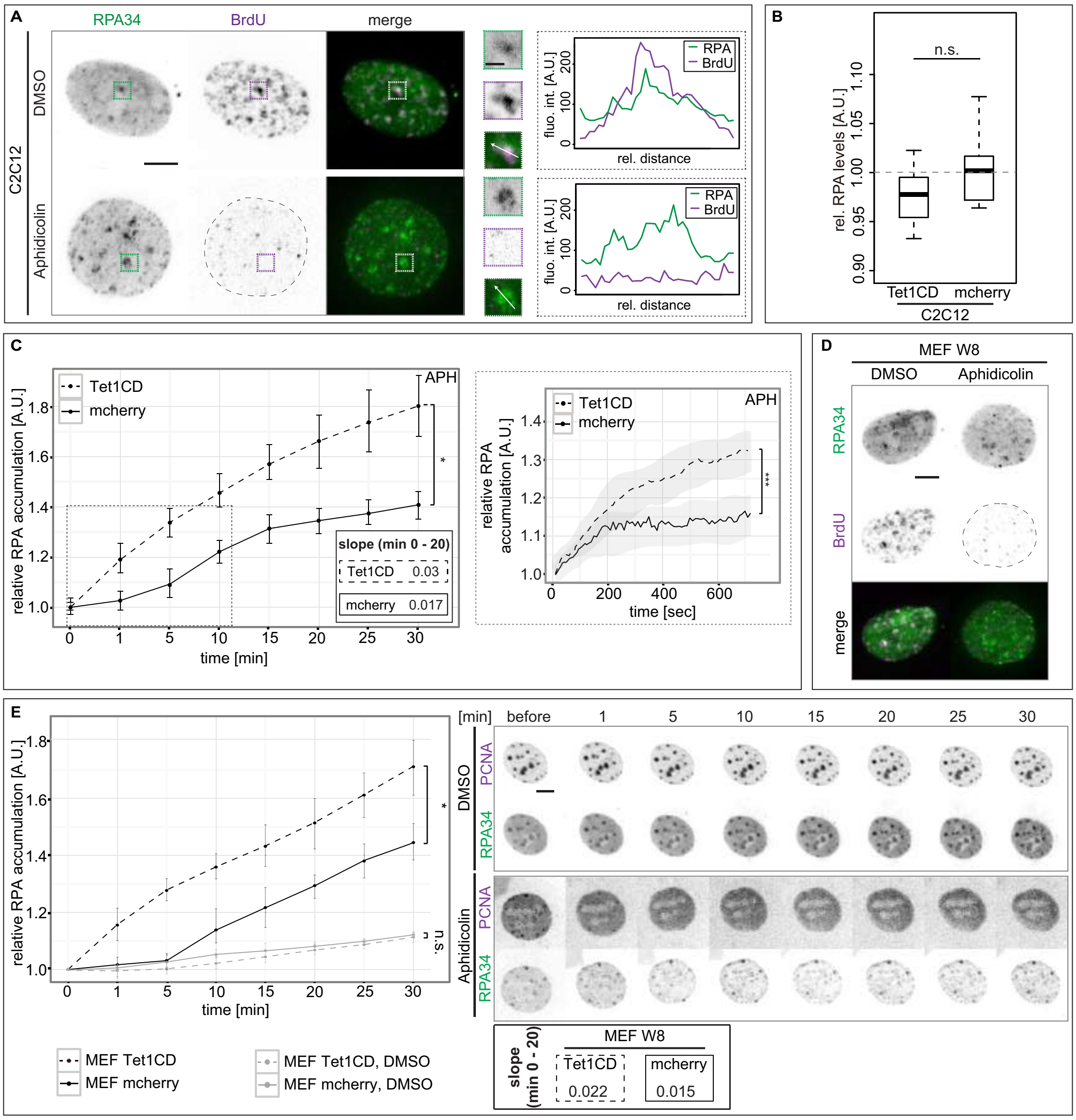

Supplementary Figure 16 – Effect of cytosine modifications on DNA helicase activity *in vivo*. (A) Representative spinning disk confocal images showing the localization of GFP-RPA and the (lack of) BrdU incorporation in late C2C12 S-phase cells after DMSO or aphidicolin treatment. Dashed boxes represent the selected magnified ROIs for line intensity profile analysis (arrows). Line intensity plots of BrdU (magenta) and RPA (green) through a replicating chromocenter in late C2C12 S-phase cells before and 30 minutes after aphidicolin/DMSO addition are shown. **(B)** Relative GFP-RPA sum intensities 24 hours after C2C12 triple transfection, showing no significant difference of RPA levels within the different transfection conditions. **(C)** Graphs showing the normalized average RPA accumulation (c_v ± standard deviation) over 30 minutes at 5 minutes interval imaging (left) and over 12 minutes at 10 seconds interval (right) for aphidicolin treated C2C12 S-phase cells, respectively (as in Figure 6B). Slopes of the RPA accumulation were calculated as in Figure 6B. **(D)** Representative spinning disk confocal images of BrdU incorporation in aphidicolin and DMSO treated MEF W8 cells. **(E)** Analysis, representation and slopes of RPA accumulation for triple transfected MEF W8 cells are as in (**B**). Representative spinning disk confocal images of time-lapse microscopy upon aphidicolin treatment of showing RPA and PCNA localization in aphidicolin and DMSO treated MEF cells. Scale bars = 5 µm (whole nuclei images) and 2.5 µm (magnifications). All boxes and whiskers are as in Supplementary Figure 10 and *p* and n-values are summarized in Supplementary Table 8. * $p < 0.05$ and n.s.: non-significant.

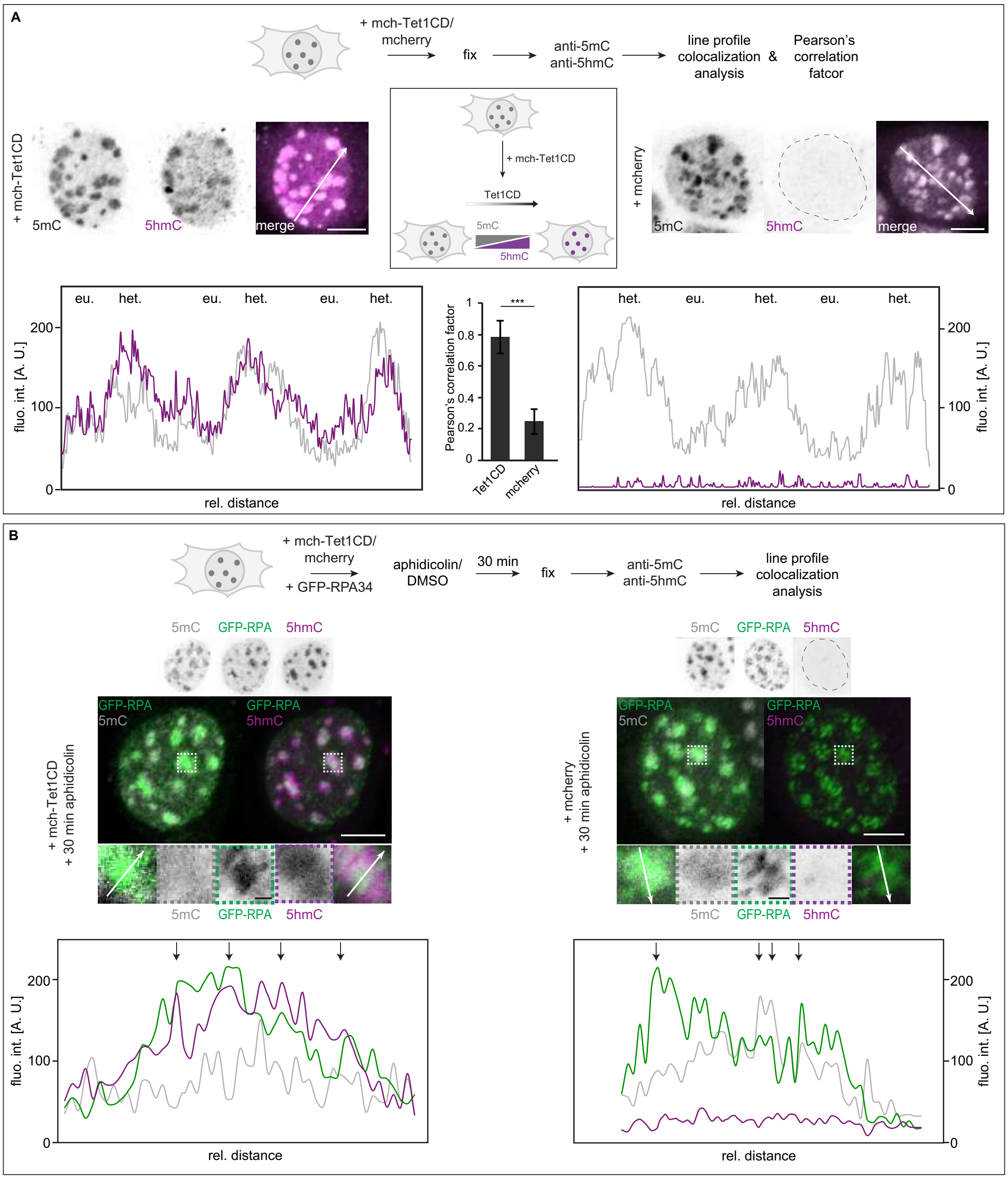

Supplementary Figure 17 – Colocalization analyses of cytosine modifications and DNA replication in mcherry-Tet1CD/mcherry transfected cells. (A) 24 hours after mcherry-Tet1CD/mcherry transfection, cells were fixed and co-stained for 5mC (grey) and 5hmC (magenta). Line profile analyses and Pearson's correlation factors (mean ± StDev) of 5mC and 5hmC are shown. Line profiles are measured along the white arrow depicted in the images. **(B)** 24 hours after mcherry-Tet1CD/mcherry and GFP-RPA double transfection, cells were treated for 30 minutes with aphidicolin or DMSO, fixed and co-stained for 5mC and 5hmC. Line profile analyses of late S-phase for 5mC (grey), 5hmC (magenta) and GFP-RPA (green) are shown for each condition. Line profiles are measured along the white arrow depicted in the images. Black arrows in the plots highlight regions of high GFP-RPA and 5mC and/or 5hmC colocalization. Scale bar = 5 µm (whole nuclei) and 2.5 µm (magnifications). Dotted lines represent nuclei contours. eu.: euchromatin, het.: heterochromatin and *** $p < 0.001$.

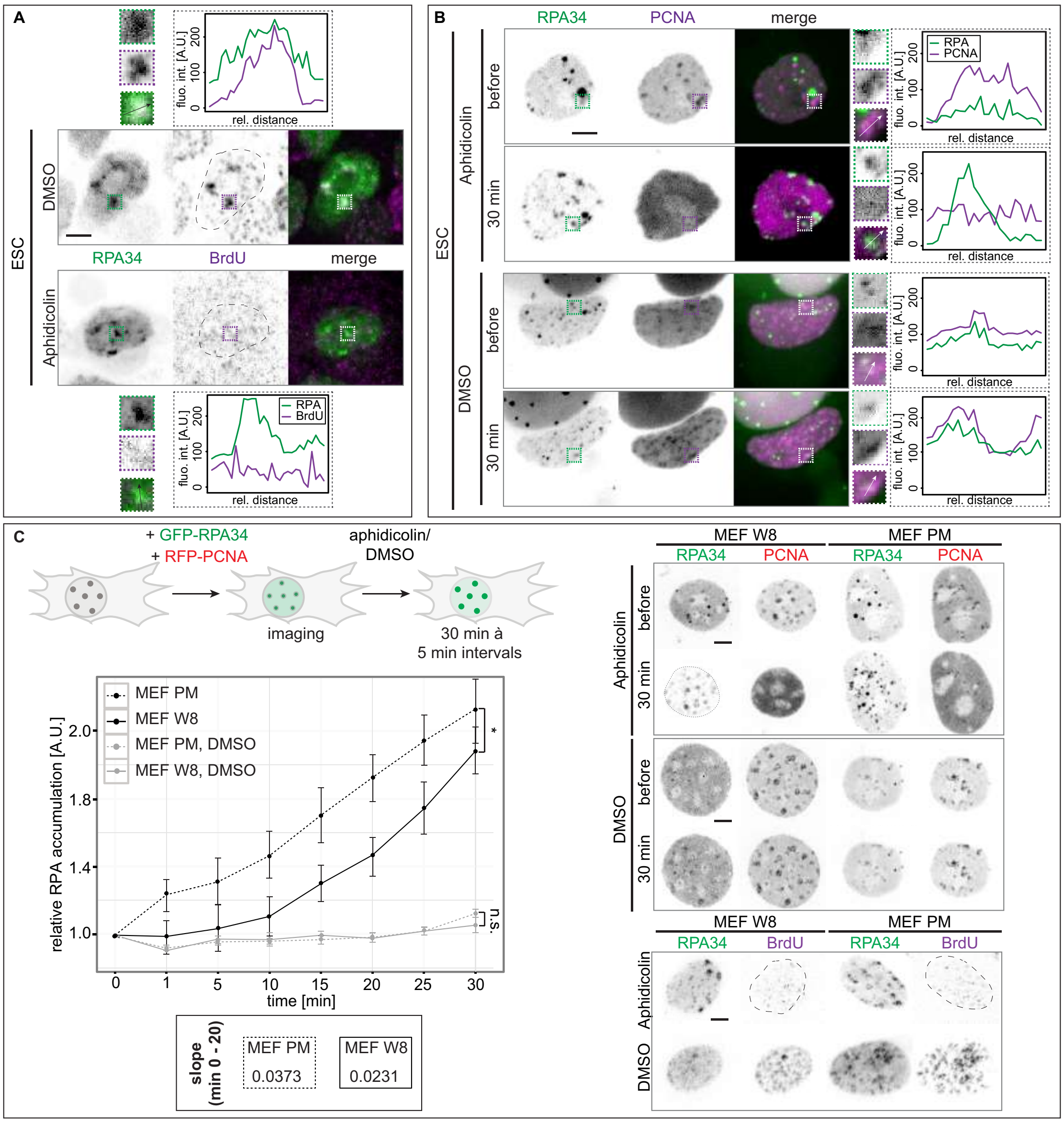

Supplementary Figure 18 – Effect of loss of cytosine methylation on DNA helicase activity *in vivo*. (A) Representative spinning disk confocal images showing the localization of GFP-RPA and the (lack of) BrdU incorporation in stage II J1 ES cells after aphidicolin/DMSO treatment. Dashed boxes represent the selected magnified ROIs for line intensity profile analysis (white arrows). Line intensity plots of BrdU and RPA are as described in Figure 6B. **(B)** Representative spinning disk confocal time-lapse microscopy images of aphidicolin and DMSO treated J1 cells as in Figure 6B. Dashed boxes represent the selected magnified ROIs for line intensity profile analysis (arrows) before and 30 minutes after aphidicolin/DMSO addition. **(C)** MEF W8 and MEF PM cells were co-transfected with GFP-RPA and RFP-PCNA 24 hours before imaging and imaging and analysis of RPA accumulation and slopes of double transfected MEF cells as in Figure 6B. Representative spinning disk confocal images before and after 30 minutes of aphidicolin/DMSO treatment showing RPA localization and BrdU incorporation after 30 minutes of aphidicolin/DMSO treatment are shown. Dashed lines represent nuclear contours. Scale bars = 5 µm. All boxes and whiskers are as in Supplementary Figure 10. *p* and n-values are summarized in Supplementary Table 8. * $p < 0.05$ and n.s.: non-significant.

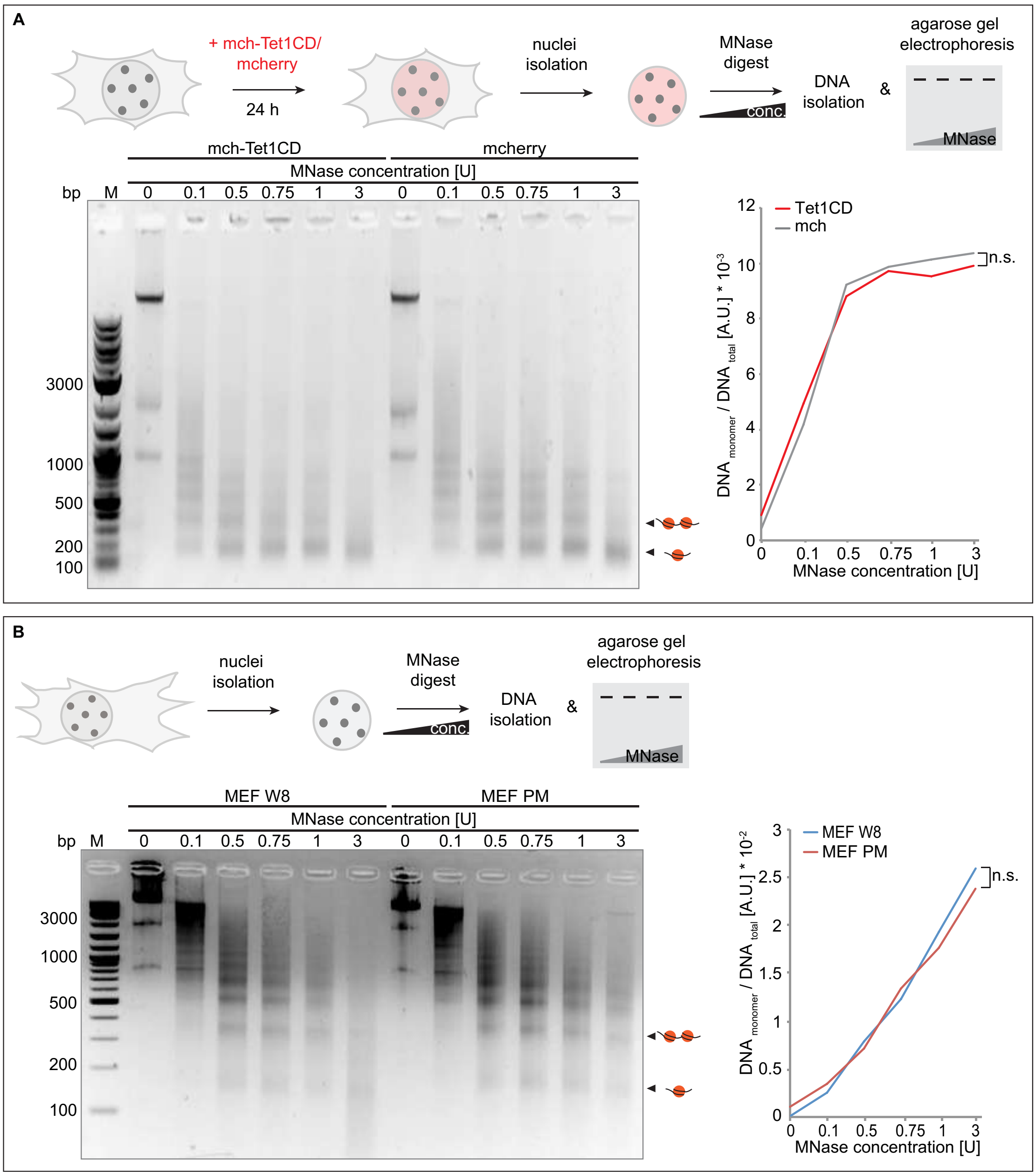

Supplementary Figure 19 – Effect of cytosine modifications on chromatin structure. (A-B) Experimental setup for micrococcal nuclease (MNAse) assay in mch-Tet1CD or mcherry transfected C2C12 myoblast cells (**A**) and for MEF W8 and MEF PM cells (**B**). Agarose gel electrophoretic analysis showing MNase digest of mch-Tet1CD and mcherry transfected cells (**A**) and of MEF W8 and MEF PM cells (**B**) and quantification of sum mononucleosomal DNA band intensities normalized to the DNA input (0 U MNase). Shown is one representative gel, experiments were done with two independently isolated sets of genomic DNA. n.s.: non-significant.

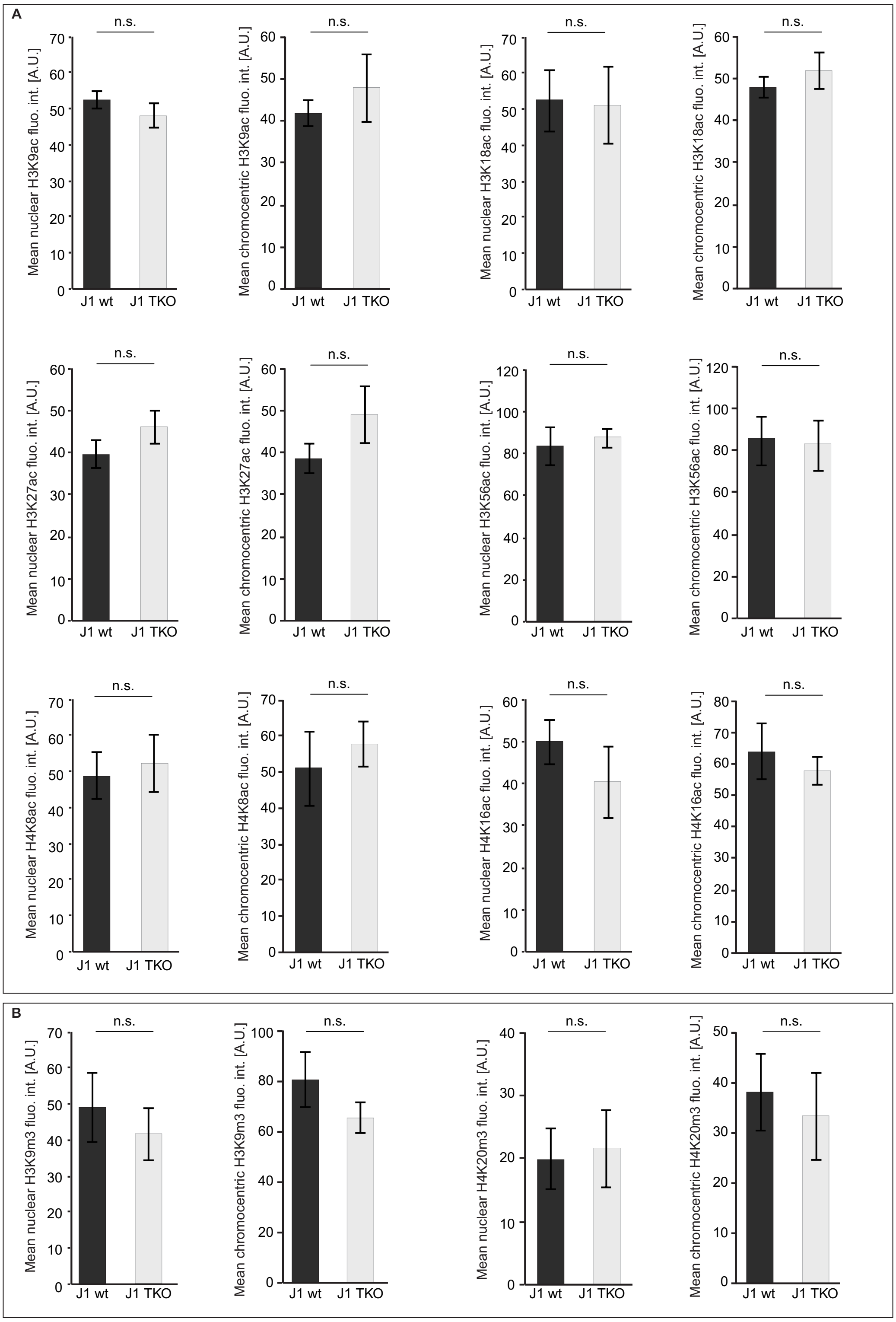

Supplementary Figure 20 – Effect of loss of global DNA methylation on histone modifications in ES cells. (A-B) Mouse J1 and TKO ES cells were pulse labelled with EdU, fixed and EdU was detected in combination with histone modifications. The nuclear and chromocentric levels of histone acetylation (**A**, H3K9ac, H3K18ac, H3K27ac, H3K56ac, H4K8ac and H4K16ac) and methylation (**B**, H3K9m3 and H4K20m3) were measured in non-replicating (EdU negative) cells. Error bars represent standard deviation. Independent experiments were done in duplicates. *p* and n-values are summarized in Supplementary Table 8. n.s.: non-significant.

REFERENCES

1. Weber, P., Rausch, C., Scholl, A. and Cardoso, M.C. (2019) Repli-FISH (Fluorescence in Situ Hybridization): Application of 3D-(Immuno)-FISH for the study of DNA replication timing of genetic repeat elements. *OBM Genetics*, **3**.
2. Zhang, P., Hastert, F.D., Ludwig, A.K., Breitwieser, K., Hofstätter, M. and Cardoso, M.C. (2017) DNA base flipping analytical pipeline. *Biology Methods and Protocols*, **2**, 1-12.
3. Zhang, P., Ludwig, A.K., Hastert, F.D., Rausch, C., Lehmkuhl, A., Hellmann, I., Smets, M., Leonhardt, H. and Cardoso, M.C. (2017) L1 retrotransposition is activated by Ten-eleven-translocation protein 1 and repressed by methyl-CpG binding proteins. *Nucleus*, **8**, 548-562.
4. Merkl, P.E., Pilsl, M., Fremter, T., Schwank, K., Engel, C., Längst, G., Milkereit, P., Griesenbeck, J. and Tschochner, H. (2020) RNA polymerase I (Pol I) passage through nucleosomes depends on Pol I subunits binding its lobe structure. *Journal of Biological Chemistry*, **295**, 4782-4795.
5. Ludwig, A.K., Zhang, P., Hastert, F.D., Meyer, S., Rausch, C., Herce, H.D., Müller, U., Lehmkuhl, A., Hellmann, I., Trummer, C. *et al.* (2017) Binding of MBD proteins to DNA blocks Tet1 function thereby modulating transcriptional noise. *Nucleic Acids Research*, **45**, 2438-2457.
6. Sporbert, A., Domaing, P., Leonhardt, H. and Cardoso, M.C. (2005) PCNA acts as a stationary loading platform for transiently interacting Okazaki fragment maturation proteins. *Nucleic acids research*, **33**, 3521-3528.
7. Sporbert, A., Gahl, A., Ankerhold, R., Leonhardt, H. and Cardoso, M.C. (2002) DNA polymerase clamp shows little turnover at established replication sites but sequential de novo assembly at adjacent origin clusters. *Mol Cell*, **10**, 1355-1365.
8. Zillmann, M., Zapp, M.L. and Berget, S.M. (1988) Gel electrophoretic isolation of splicing complexes containing U1 small nuclear ribonucleoprotein particles. *Mol Cell Biol*, **8**, 814-821.
9. Yaffe, D. and Saxel, O. (1977) Serial passaging and differentiation of myogenic cells isolated from dystrophic mouse muscle. *Nature*, **270**, 725-727.
10. Peters, A.H., O'Carroll, D., Scherthan, H., Mechtler, K., Sauer, S., Schofer, C., Weipoltshammer, K., Pagani, M., Lachner, M., Kohlmaier, A. *et al.* (2001) Loss of the Suv39h histone methyltransferases impairs mammalian heterochromatin and genome stability. *Cell*, **107**, 323-337.
11. Lande-Diner, L., Zhang, J., Ben-Porath, I., Amariglio, N., Keshet, I., Hecht, M., Azuara, V., Fisher, A.G., Rechavi, G. and Cedar, H. (2007) Role of DNA methylation in stable gene repression. *J Biol Chem*, **282**, 12194-12200.
12. Graham, F.L., Smiley, J., Russell, W.C. and Nairn, R. (1977) Characteristics of a human cell line transformed by DNA from human adenovirus type 5. *The Journal of general virology*, **36**, 59-74.
13. Li, E., Bestor, T.H. and Jaenisch, R. (1992) Targeted mutation of the DNA methyltransferase gene results in embryonic lethality. *Cell*, **69**, 915-926.
14. Tsumura, A., Hayakawa, T., Kumaki, Y., Takebayashi, S., Sakaue, M., Matsuoka, C., Shimotohno, K., Ishikawa, F., Li, E., Ueda, H.R. *et al.* (2006) Maintenance of self-renewal ability of mouse embryonic stem cells in the absence of DNA methyltransferases Dnmt1, Dnmt3a and Dnmt3b. *Genes Cells*, **11**, 805-814.
15. Rottach, A., Kremmer, E., Nowak, D., Leonhardt, H. and Cardoso, M.C. (2008) Generation and characterization of a rat monoclonal antibody specific for multiple red fluorescent proteins. *Hybridoma*, **27**, 337-343.
